# A systematic review of trials evaluating success factors of interventions with computerised clinical decision support

**DOI:** 10.1186/s13012-018-0790-1

**Published:** 2018-08-20

**Authors:** Stijn Van de Velde, Annemie Heselmans, Nicolas Delvaux, Linn Brandt, Luis Marco-Ruiz, David Spitaels, Hanne Cloetens, Tiina Kortteisto, Pavel Roshanov, Ilkka Kunnamo, Bert Aertgeerts, Per Olav Vandvik, Signe Flottorp

**Affiliations:** 1 Centre for Informed Health Choices, Division for Health Services, Norwegian Institute of Public Health, Oslo, Norway; 2 Department of Public Health and Primary Care, KU Leuven, Leuven, Belgium; 3 MAGIC non-profit research and innovation programme, Oslo, Norway; 4 Department of Medicine, Innlandet Hospital Trust, Gjøvik, Norway; 5 Institute of Health and Society, University of Oslo, Oslo, Norway; 6 Norwegian Centre for E-health Research, Tromsø, Norway; 7 Flemish College of General Practitioners, Antwerp, Belgium; 8 Department of Internal Medicine, Tampere University Hospital, Tampere, Finland; 9 Department of Medicine, McMaster University, Hamilton, Canada; 10 Duodecim, Scientific Society of Finnish Physicians, Helsinki, Finland

**Keywords:** Clinical computerised decision support systems, Practice guidelines, Guideline adherence, Evidence-based medicine, Implementation, Systematic review

## Abstract

**Background:**

Computerised clinical decision support (CDS) can potentially better inform decisions, and it can help with the management of information overload. It is perceived to be a key component of a learning health care system. Despite its increasing implementation worldwide, it remains uncertain why the effect of CDS varies and which factors make CDS more effective.

**Objective:**

To examine which factors make CDS strategies more effective on a number of outcomes, including adherence to recommended practice, patient outcome measures, economic measures, provider or patient satisfaction, and medical decision quality.

**Methods:**

We identified randomised controlled trials, non-randomised trials, and controlled before-and-after studies that directly compared CDS implementation with a given factor to CDS without that factor by searching CENTRAL, MEDLINE, EMBASE, and CINAHL and checking reference lists of relevant studies. We considered CDS with any objective for any condition in any healthcare setting. We included CDS interventions that were either displayed on screen or provided on paper and that were directed at healthcare professionals or targeted at both professionals and patients. The reviewers screened the potentially relevant studies in duplicate. They extracted data and assessed risk of bias in independent pairs or individually followed by a double check by another reviewer. We summarised results using medians and interquartile ranges and rated our certainty in the evidence using the GRADE system.

**Results:**

We identified 66 head-to-head trials that we synthesised across 14 comparisons of CDS intervention factors. Providing CDS automatically versus on demand led to large improvements in adherence. Displaying CDS on-screen versus on paper led to moderate improvements and making CDS more versus less patient-specific improved adherence modestly. When CDS interventions were combined with professional-oriented strategies, combined with patient-oriented strategies, or combined with staff-oriented strategies, then adherence improved slightly. Providing CDS to patients slightly increased adherence versus CDS aimed at the healthcare provider only. Making CDS advice more explicit and requiring users to respond to the advice made little or no difference. The CDS intervention factors made little or no difference to patient outcomes. The results for economic outcomes and satisfaction outcomes were sparse.

**Conclusion:**

Multiple factors may affect the success of CDS interventions. CDS may be more effective when the advice is provided automatically and displayed on-screen and when the suggestions are more patient-specific. CDS interventions combined with other strategies probably also improves adherence. Providing CDS directly to patients may also positively affect adherence. The certainty of the evidence was low to moderate for all factors.

**Trial registration:**

PROSPERO, CRD42016033738

**Electronic supplementary material:**

The online version of this article (10.1186/s13012-018-0790-1) contains supplementary material, which is available to authorized users.

## Introduction

The amount of knowledge required to make well-informed health choices is moving beyond unassisted human capacity [1]. Computerised clinical decision support (CDS) can potentially better inform decisions, and it can help with the management of information overload [1, 2]. According to the US Institute of Medicine, CDS is a key component of a learning health care system where new knowledge makes its way into practice without undue delays [1]. CDS technology uses patient-specific data to provide relevant medical knowledge at the point of need. Worldwide, the implementation of this quality improvement intervention is increasing. CDS interventions are also becoming larger and more complex [1, 3].

Summaries of the evidence regarding CDS effectiveness estimate modest increases in guideline adherence [4] and modest reductions in morbidity [5]. However, there is considerable variation in the ability of CDS intervention to produce the desired results: some trials report large increases in adherence to recommended practice while others find little or no change, and some reported unintended negative consequences [6–8]. It is unclear how to best implement CDS and achieve better outcomes, costs, and satisfaction with healthcare [9, 10].

Several reviews have examined why some CDS efforts succeed and others fail [11–14]. These reviews suggested that multiple factors (such as providing decision support to both clinicians and patients and giving recommendations rather than only assessments) may correlate with greater CDS success. However, the findings in these reviews were derived by meta-regression analyses (an observational type of analysis) and are therefore more prone to bias than studies with direct comparisons. Further, some of the findings have been inconsistent: some reviews have suggested that CDS that is integrating with electronic charting and order entry systems is associated with greater chance of success while others suggest the opposite [11, 15, 16].

To our knowledge, only one study has systematically reviewed head-to-head trials and provided direct comparisons of factors modifying the success of CDS [12]. The review was limited to 11 trials of both manual- and computer-based decision support, all of which were published before 2004. Many new trials have been published since then, and there is a need for a new review of head-to-head trials.

We conducted this systematic review to examine which factors make CDS strategies more (or less) effective on a number of outcomes, including adherence to recommended practice, patient outcome measures, economic measures, provider or patient satisfaction, and medical decision quality, and based on direct evidence from studies that compare one strategy to another. The review also informs the GUideline Implementation with Decision Support (GUIDES) project where we develop a checklist to help CDS implementation teams increase the success of CDS [17].

## Methods

The author group includes experts with a strong commitment to evidence-based medicine and informed health choices and broad expertise related to the development, implementation, and evaluation of CDS.

### Protocol and registration

We registered the protocol for this systematic review in the PROSPERO database (CRD42016033738) [17].

### Study selection criteria

We included randomised and non-randomised controlled trials and controlled before-and-after studies. We excluded observational studies and studies without a control group (such as uncontrolled before-and-after studies and interrupted time series studies).

We included studies of computer-generated decision support that was either displayed on screen or provided on paper and directed at healthcare professionals or targeted at both professionals and patients in any healthcare setting. We excluded studies in which the population was limited to simulated patients or to the use of CDS by students only.

We considered CDS with any objective (e.g. diagnosis, treatment, test ordering, screening) for any health condition.

We included studies that directly compared an intervention with CDS that featured a factor that could potentially affect intervention success (such as directing CDS at clinicians and/or patients, having to provide reasons for not adhering to advice, the automatic provision of CDS versus on-demand CDS, providing advice linked to evidence-based information, the endorsement of CDS by opinion leaders, the timing or frequency of the decision support, using actionable CDS that makes it easy to adhere to the advice, and providing training in the use of CDS) versus an intervention with CDS that did not feature that factor.

We also included trials with CDS in both arms that evaluate the effect of any adjacent interventions to the CDS (e.g. CDS combined with patient-oriented strategies versus CDS only). We did not predefine inclusion criteria in terms of the success features; instead, we defined the list of success factors by the type of comparisons that we identified. We used the GUIDES framework to categorise the comparisons according to different factors that may affect the success of CDS interventions [18].

To qualify for inclusion in this systematic review, studies had to include an assessment of at least one outcome specified in the main categories of outcomes presented by the Cochrane Effective Practice and Organisation of Care (EPOC) review group, namely patient outcomes, quality of care, utilisation or coverage of services, resource use, healthcare provider outcomes (for example workload or burnout), social outcomes, equity, and adverse effects [19]. We also included studies with outcomes on the satisfaction of healthcare providers and/or patients and on medical decision quality.

We excluded studies in which use of the CDS or compliance with its advice was mandatory. We also excluded papers in which the intervention focused on reminder messages for attendance at upcoming healthcare appointments.

### Information sources and search

We searched for relevant studies in the Cochrane Central Register of Controlled Trials (CENTRAL) through The Cochrane Library (http://mrw.interscience.wiley.com/cochrane/), MEDLINE and EMBASE through the Ovid platform (www.ovid.com), and CINAHL, through EBSCO (the search was conducted on 20 December 2016). To further identify relevant studies, the reference lists of relevant systematic reviews and trials were screened, and we used our own files of relevant studies. We did not apply language or publication period restrictions. The search string (Additional file 1) for this systematic review was based on the search string from related Cochrane reviews and the terms available in a set of 26 relevant studies that we were aware of before starting the search [20, 21].

### Selection of studies

Four reviewers (AH, ND, PR, SV) worked in pairs to select studies from titles and abstracts, to screen potentially relevant full texts, and to exclude studies that did not meet the inclusion criteria. The reviewers resolved disagreements on the selection of studies by discussion. We used Covidence systematic review software throughout this process (https://www.covidence.org).

### Data extraction and risk of bias assessment

We involved seven reviewers (AH, DS, HC, LB, LMR, ND, SV) to extract data from the selected studies and to evaluate the quality of the included studies. Pairs of reviewers independently extracted data and assessed risk of bias for half of the studies. For the remaining studies, a single reviewer extracted the data and appraised the risk of bias, and another reviewer checked the summaries with the original papers. We contacted the authors of the trials published in the last 10 years to obtain further details on missing or unclear data fields.

The reviewers used the EPOC data collection checklist which we modified for the needs of this review and the risk of bias criteria suggested by EPOC [19].

Summary measures only were used in instances in which studies reported both summary measures (e.g. all cause morbidity) and specific measures (subscales for different dimensions on quality of life). We extracted the outcomes for the longest available follow-up interval if the studies reported multiple follow-up intervals. We extracted secondary outcome data that were not subject to statistical testing in the primary study if this comparison was of interest. Some studies made within-group comparisons instead of comparisons across groups. In such situations, we used the available data and compared the results across the relevant trial arms while adjusting for baseline values. Other studies compared outcomes (from different relevant trial arms) with a usual care control group. In such instances, we extracted the data for the comparison groups of interest for our review and compared the results for those groups.

For each study, we made a summary assessment of the risk of bias. If we judged at least one risk of bias domain to be high risk, we categorised the study as having high risk of bias. If we judged one or more domains to be an unclear risk and no domains were high risk, we categorised the study as having an unclear risk of bias. If all the risk of bias domains were low risk or if it was unlikely that any bias could have had a serious impact, we judged the study to have a low risk of bias [19]. We used the GRADE approach to assess the certainty of evidence for each comparison/outcome [22].

### Data synthesis

Given the heterogeneity of the identified studies, we did not use standard meta-analysis techniques. An additional issue is that the data was derived from both cluster and non-cluster randomised trials. If clustering is not taken into account when conducting the statistical analysis of cluster RCTs, unit of analysis errors occur leading to misleading low *p* values and narrow confidence intervals [23]. Cluster RCTs were the dominant design of the included studies. We therefore planned an alternative strategy in the protocol based on EPOC methods for results for which the use of a meta-analysis is not appropriate [19]. This alternative method, which uses medians and interquartile ranges to present summary measures, was first developed by Grimshaw et al. in a review of guideline dissemination and implementation strategies and has been used in multiple Cochrane EPOC reviews [21, 24–29].

In this review, we report outcomes in the following categories: dichotomous process measures, continuous process measures, dichotomous patient measures, and continuous patient measures. We also report on economic and satisfaction measures and measures of the use of CDS resources.

#### Primary analyses

The primary analyses of this review focus on dichotomous process measures that reflect adherence to recommended practice and on dichotomous patient outcome measures that reflect changes in clinical outcomes. We selected these measures for the primary analyses because behaviour change is a key goal of CDS, and the interpretation of the magnitude of change for dichotomous outcomes is sufficiently consistent to allow comparisons across interventions. Dichotomous process measures were also the most frequently evaluated outcome measure. Better informed medical decisions are also a key goal of CDS, but these were rarely evaluated.

We only included studies that provided data on baseline outcomes in the primary analyses. We adjusted risk differences (RD) for baseline outcome values [26, 27]. Baseline outcome differences occur frequently in cluster randomised trials and unadjusted effect measures can bias the analysis. We calculated the adjusted RD as (Intervention %_post_ − Comparison %_post_) − (Intervention %_pre_ − Comparison %_pre_). A RD of, for example, 12% represents an absolute improvement of 12 percentage points in adherence to the recommended practice (or for patient outcomes when these are dichotomous).

The primary analyses were also limited to data from the primary outcome that the study authors had defined in their articles. For studies in which the authors had not defined a primary outcome, we defined the primary outcome as the one that had been used for the sample size calculation [25]. If the primary outcome was not clear, we calculated the median effect size across all the outcomes per outcome category.

#### Secondary analyses

In the secondary analysis, we explored the consistency of the primary analyses with the results of the unadjusted differences (if no baseline outcome data was provided) and with the results of the other outcome measures that were not included in the primary analyses [29]. For continuous outcomes that provided baseline outcome data, we calculated the adjusted change relative to the comparison group as {(Intervention mean_post_ − Comparison mean_post_) − (Intervention mean_pre_ − Comparison mean_pre_)} / Comparison mean_post_. We did not report medians and interquartile ranges as summary estimates for continuous measures because the magnitude of relative change for a continuous outcome measure depends on the scale being used when the mean difference is not standardised.

#### Subgroup analysis

For the comparison ‘CDS combined with patient-oriented strategies versus CDS only’, we explored variation in summary estimates based on the type of targeted behaviour. We compared the summary estimates for CDS targeted at prevention and screening, versus CDS targeted at treatment of acute or chronic diseases. We made this comparison, because reminders for vaccinations could potentially be more effective [30]. We used the Mann-Whitney two-sample test to compare the effects for the two subgroups of studies.

### Reporting

We described the results for dichotomous process measures as follows: ‘small’ for RD values of < 5%, ‘modest’ for RD values from 5 to < 10%, ‘moderate’ for RD values from 10 to < 20%, and ‘large’ for RD values ≥ 20% [24].

In some studies, an improvement corresponded to an increase in the measure (higher values indicated improvement). In others, an improvement corresponded to a decrease in the measure (lower values indicated improvement). We therefore standardised the direction of effect, so that higher values represented an improvement.

Some studies compared groups in which the intervention differed in relation to more than one CDS factor (e.g. CDS provided at a different point in time and delivered through a different channel). In such situations, the study was not included in the analysis but the findings are reported as an additional note to the comparison.

### Differences between the protocol and review

Our decision to use the two-step approach, focusing initially on the primary and then on the secondary analysis, was taken after publication of the protocol but before starting on the data synthesis. We decided to perform the subgroup analysis after the data synthesis was completed.

## Results

### Study selection

**Fig. 1:**
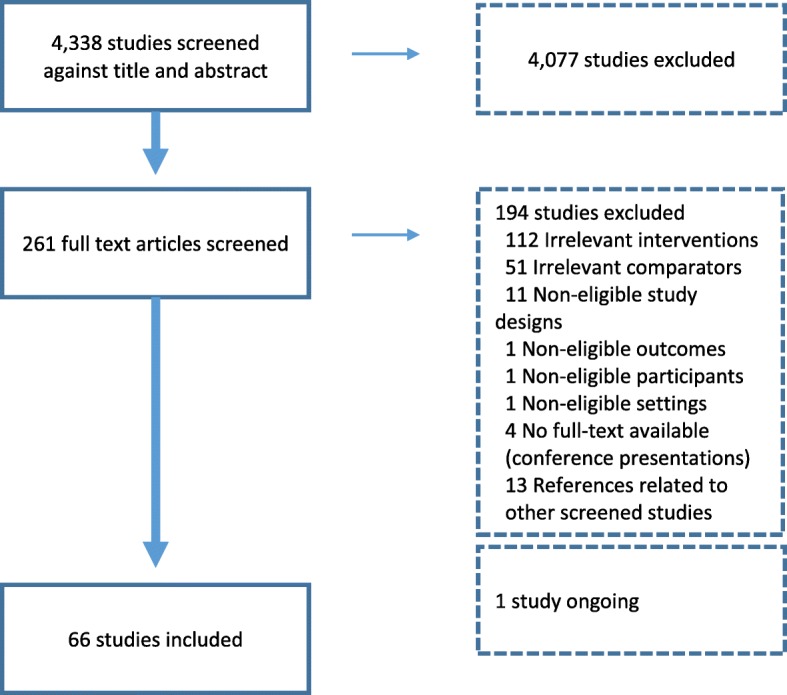
Study selection flowchart

We screened 4338 studies and included 66 in the review. Figure 1 provides further details about the selection process.

### Study characteristics

Most studies (53/66; 80%) had clustered designs. Fifty-three studies (80%) were conducted in the USA, five in Canada, and five in the Netherlands. The three remaining studies were conducted in Israel, New Zealand, and Switzerland.

Forty-one studies (62%) targeted only the healthcare provider, and 25 studies (38%) targeted both the healthcare provider and the patient. Fifty-eight studies (88%) were conducted in clinical outpatient settings, six studies were in inpatient settings (9%), and two studies (3%) were based in both. The purpose of the CDS was to support decision-making in preventive care in 22 studies (33%), acute conditions in 4 studies (6%), chronic conditions in 22 studies (33%), and management of miscellaneous conditions (e.g. CDS related to prevention and/or acute conditions and/or chronic conditions) in 18 studies (27%).

The CDS was delivered on-screen to the healthcare professionals in 41 studies (62%) and on paper in 20 studies (30%). In three studies, the CDS was delivered using both on-screen and paper methods; in two studies, it was unclear how the CDS has been provided. Twelve studies (18%) were published before 2000; 30 studies (46%) between 2000 and 2010; and 24 studies (36%) between 2010 and 2016.

Fifty-five studies (83%) assessed dichotomous process measures and 15 studies (23%) evaluated dichotomous patient measures. Fourteen studies (21%) reported continuous process outcomes; and continuous patient outcomes were reported in 16 studies (24%). Fourteen studies measured economic outcomes (21%), three studies evaluated patient satisfaction, and two studies reported provider satisfaction. Dichotomous process outcomes measured typically the proportion adherence with guidelines. Continuous process outcomes included for example the number of tests ordered, mean drug doses, rates of use of medications to avoid, or a continuity of care score. Dichotomous patient outcome measures included for example proportion of patients with clinical improvement, with abnormal test results or with various types of morbidity. Examples of continuous patient outcomes included mean blood pressure, quality of life score, or number of hospitalisations or emergency department visits.

### Risk of bias within studies

**Fig. 2:**
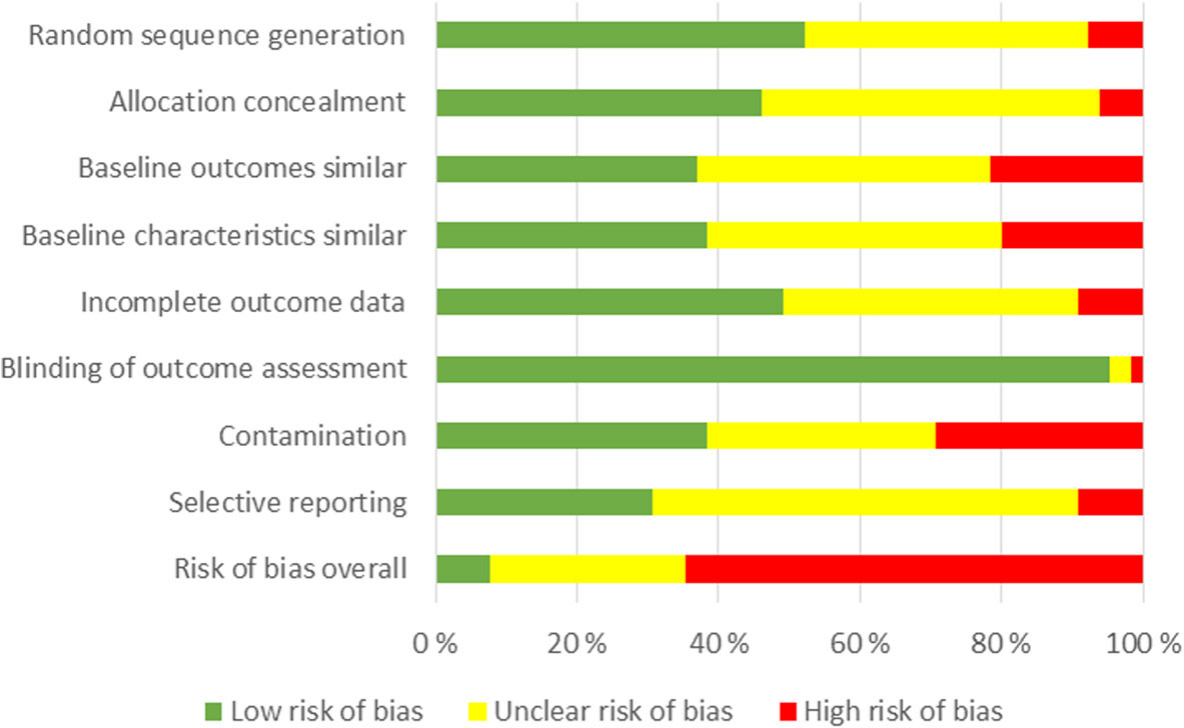
Risk of bias assessment

Most studies (43/66; 65%) were at high risk of bias. Most frequently, the causes of a high risk of bias included contamination between comparison arms and differences in baseline characteristics and baseline outcomes (Fig. 2). Five (8%) were at low risk of bias and risk of bias was unclear in 18 studies (27%). Four studies (6%) received commercial funding.

### Results of individual studies

Data for each individual trial is available in Additional files 2 and 3.

### Synthesis of results—effect of CDS intervention factors across studies

Table 1 provides an overview of the results of the primary and secondary analyses for adherence to recommended practice and for patient outcomes. The data is limited to dichotomous outcomes. Detailed description and summary of finding tables for all the comparisons are available in Additional file 4.

**Table 1:** Overview of the main results

Factor	Outcome	Number of studies*	Absolute improvement (RD)^Ɨ^	Certainty of evidence (GRADE)^ǂ^
More versus less evidence-based CDS advice	Adherence	–	–	⊕⊕○○
		*2*	*5.0%* *Range of ORs: 3.5 (CI 1.1–11.5) to 1.0 (CI 0.3–2.8)*	Low
More versus less patient-specific CDS (by using additional patient data)	Adherence	1	6.2%	⊕⊕⊕○
		*3*	*3.0%, IQR 1.2 to 5.9*	Moderate
	Patient outcomes	–	–	⊕⊕○○
		*3*	*8%, IQR 0 to 8.9*	Low
More versus less explicit CDS advice (by providing recommendations or not)	Adherence	1	−0.4%	⊕⊕⊕○
		*1*	*1.0%*	Moderate
	Patient outcomes	–	–	⊕⊕⊕○
		*1*	*− 0.7%*	Moderate
More versus less explicit CDS advice (by presenting specific patient data or not)	Adherence	–	–	⊕⊕○○
		*1*	*− 4.0%*	Low
CDS that does (versus does not) require users to respond to the advice	Adherence	1	0.1%	⊕⊕○○
		*3*	*− 2.0%, IQR − 2.9 to 8.0*	Low
CDS provided automatically by the system versus on demand by the user	Adherence	–	–	⊕⊕○○
		*3*	*22.2%, IQR 9.4 to 28.0*	Low
CDS displayed on screen versus delivered on paper	Adherence	1	15.6%	⊕⊕○○
		*1*	*Range of ORs 0.3 (CI 0.1–1.0) to 0.7 (CI 0.2–2.1)*	Low
CDS combined with other professional-oriented strategies versus CDS only	Adherence	3	4.8%, IQR *−* 3.9 to 10.8	⊕⊕○○
		*1*	*6.2%*	Low
	Patient outcomes	–	–	⊕⊕○○
		*2*	*− 0.5, IQR* − *5 to 4*	Low
CDS combined with patient-oriented strategies versus CDS only	Adherence	10	3.1%, IQR − 2.0 to 5.0	⊕⊕○○
		*5*	*2.8%, IQR 1.5 to 6.5*	Moderate
	Patient outcomes	1	− 5%	⊕⊕○○
		*1*	*18.2%*	Low
CDS aimed at the patient versus CDS aimed at the healthcare provider	Adherence	3	5.1%, IQR − 5.3 to 13.4	⊕⊕○○
		*2*	*4.2%, IQR 0 to 8.3*	Low
	Patient outcomes	–	–	⊕⊕○○
		*2*	− *2.4%, IQR* − *8.1 to 3.3*	Low
CDS for physician and another provider type versus CDS for physician only	Adherence	1	4.1%, IQR 3.4 to 7.2	⊕⊕⊕○
		*2*	*5.2%, IQR 4.4 to 6*	Moderate
	Patient outcomes	–	–	⊕⊕○○
		*2*	*1.4% (IQR + 1 to + 1.7)*	Low

*The upper row presents the results for the primary analyses (normal print) and the lower row presents the secondary analyses data (*in italics*). The primary analyses only included studies with risk differences that were adjusted for baseline differences; the secondary analyses present results for studies where it was not possible to adjust for baseline differences

^Ɨ^The results of the studies are presented as absolute improvement (risk difference). We present the median and interquartile range (IQR) if multiple studies were available for an analysis. Odds ratios (OR) and 95% confidence intervals (CI) are presented if no risk data was available

^ǂ^GRADE Working Group grades of evidence: High certainty ⊕⊕⊕⊕: Further research is very unlikely to change our confidence in the estimate of effect. Moderate certainty ⊕⊕⊕⃝: Further research is likely to have an important impact on our confidence in the estimate of effect and may change the estimate. Low certainty ⊕⊕⃝⃝: Further research is very likely to have an important impact on our confidence in the estimate of effect and is likely to change the estimate. Very low certainty ⊕⃝⃝⃝: We are very uncertain about the estimate

We synthesised the trials across 14 factors based on the comparisons of aspects related to the CDS intervention. Five comparisons related to the content of the CDS, five comparisons related to the CDS system, and four comparisons related to the implementation of the CDS interventions. Three comparisons are not included in Table 1. The comparison ‘CDS provided before versus during the patient visit’ was not included because the evidence was only indirect due to the simultaneous comparison of multiple aspects related to the CDS interventions. Two comparisons are not included in Table 1 because the nature of the CDS factors was very specific (namely, comparisons in relation to the usability of the CDS system and comparisons in relation to the amount of CDS).

#### Adherence

Evidence for the effect of CDS factors on adherence ranged from no effect to large positive increases (Table 1). For example, CDS that required users to respond to advice (versus no requirement to respond) showed no effect (RD 0.1% in the primary analysis, low certainty evidence) and CDS provided automatically by the system (versus on demand) showed the largest effect (RD 22.2% in the secondary analysis, low certainty evidence). Overall, the results of the primary analyses and secondary analyses were similar. In three instances, no data were available for the primary analysis.

Displaying CDS on-screen may lead to moderate improvements in adherence (RD 15.6%, low certainty evidence), but these findings are inconsistent with the secondary analysis which showed no change. This factor included two studies, where one study targeted healthcare provider behaviour and the other study focussed on shared decision-making. Making CDS more patient-specific probably improves adherence modestly (RD 6.2% in the primary analysis, moderate certainty evidence).

Using CDS interventions combined with professional-oriented strategies (RD 4.8% in the primary analysis, low certainty evidence), combined with patient-oriented strategies such as patient education (RD 3.1% in the primary analysis, moderate certainty evidence), or combined with staff-oriented strategies (RD 4.1% in the primary analysis, moderate certainty evidence) probably improves adherence slightly. Professional-oriented strategies included for example the use of opinion leaders or educational sessions; patient-oriented strategies included for example patient education or counselling. Examples of staff-oriented strategies are the support of a case manager or the provision of CDS to different healthcare provider roles. Additional file 4 provides further details.

Providing CDS to patients may slightly increase adherence versus CDS aimed at the healthcare provider (RD 5.1% in the primary analysis, low certainty evidence). Noteworthy is the negative first quartile value, which suggests that compliance might also deteriorate when CDS is targeted directly at patients. It is uncertain from our subgroup analysis, whether the targeted behaviour (prevention/screening versus treatment) was associated with changes in adherence.

Making CDS advice more explicit and requiring users to respond to the advice may make little or no difference to adherence (low certainty evidence).

#### Patient outcomes

The amount of evidence is limited for patient outcomes, and no data were available for some of the comparisons. Overall, the different CDS intervention factors may make little or no difference to patient outcomes (low certainty evidence). Only the factor ‘more versus less patient-specific CDS’ showed modest improvement effects (RD 8% in the secondary analysis, low certainty evidence). The trials included for this factor provided CDS suggestions that were more specific to the patient situation by collecting and using additional patient data (e.g. risk factors, patient concerns). For the factor CDS to patients versus CDS aimed at the healthcare provider, we point at the findings from one study that found a 14.7% relative increase in the emergency department encounters when computer generated information was directed at the patient versus directed at a healthcare provider [31].

#### Other outcomes

The results for economic outcomes and satisfaction outcomes were sparse and could not be combined per factor. The three studies that measured patient satisfaction found no meaningful differences. Within the comparisons related to the usability of the CDS system, one study found more conducted searches, less time spent seeking information, and a higher impact of the information seeking with topic specific infobuttons [32]. For the factor more versus less evidence-based information, one study evaluated patient empowerment as an outcome, but this study found little or no change in scores [33]. Access to the decision support adjusted for the number of decision support opportunities was higher (RD 11%) when CDS was provided automatically by the system (versus on demand) [34]. Additional file 4 provides further details per factor.

## Discussion

### Summary of evidence

We synthesised 66 trials across 14 factors based on the comparisons of aspects related to the CDS intervention. The CDS intervention factors resulted in small to large improvements in adherence to recommended practice, but we found little or no difference to patient outcomes. A lack of sensitivity to measure small but relevant clinical outcomes can potentially explain this observation [35, 36]. Factors with larger interquartile ranges suggest variability with potentially larger effects when the strategy is well-designed and congruent with the local context.

The findings that CDS provided to patients improved adherence are consistent with the findings of previous meta-regression analyses [6, 11]. Lobach et al. [6] and the subgroup analysis by Shojania et al. [20] also found that CDS which is combined with professional-oriented strategies resulted in better adherence. This factor was not associated with CDS success in a review by Roshanov [11]. Evidence about the automatic provision of decision support is consistent with the findings of two meta-regression analysis by Lobach et al. and by Fillmore et al., but contradicts the findings of Roshanov et al. and the subgroup analysis by Shojania et al. [11, 15, 6, 20]. Lobach et al., Roshanov et al., and Arditi et al. found that requesting providers to confirm agreement or to provide reasons for not adhering to the advice was a factor that was associated with CDS success [6, 11, 30]. These findings were not consistent with the evidence in our review which showed little or no difference in adherence. Lobach et al. also reported that the provision of a recommendation (and not just an assessment) was more effective. This finding was not supported in the subgroup analysis by Arditi et al. and neither supported by the evidence in our review [30]. Arditi et al. and Lobach et al. also found better outcomes with decision support that are evidence-based or supported by references. Roshanov did not identify this as a success factor. More patient-specific advice was not an effect modifier in Shojania et al., while more patient-specific advice resulted in higher adherence in our review [20].

A potential reason for these differences is that the meta-regression and subgroup analyses and our review have included different studies. The meta-regression and subgroup analyses in these studies did not specifically compare CDS with and without a given factor. Instead, the analyses evaluated if studies including a factor more often were associated with success compared to studies without such a factor. Further, the studies by Arditi and by Shojania focussed on a subset of CDS trials that either delivered the advice on paper or on screen [30, 20]. The differences may also be due to bias by confounding, which is an important limitation of meta-regression analyses [37]. The majority of the trials in the meta-regression analyses included combinations of different factors that may be correlated.

### Strengths and limitations

Our systematic review identified a substantial number of head-to-head comparisons of CDS intervention factors. Our approach of using the RD for adherence, rather than relative effect sizes (e.g. relative risk), may make it easier for readers to interpret the magnitude of the changes in adherence and in patient outcomes that we report. Whenever possible, the data are corrected for baseline differences.

We have rated the certainty of the evidence using the GRADE system, and the review is more explicit than other reviews on the certainty that can be placed in the findings. Many of the studies included in this review had a high risk of bias or methods that were insufficiently clear to allow for an assessment of the risk of bias. Ideally, the evidence from all these studies should have been extracted and appraised independently by two people. But the size of this task and the limited resources available meant that only half of the studies were processed in this way. For the other studies, one author double-checked the data extraction and risk of bias assessment with the original paper as a quality control mechanism.

Given the broad inclusion criteria, the studies in this review have a wide contextual, clinical, and programmatic diversity for nearly every comparison. In our assessment of the certainty of evidence, we used the GRADE approach which takes inconsistency in the study effects into account [38]. In situations with widely differing effect estimates or in situations with only one study included in the comparison/outcome, we downgraded certainty in the evidence. While different contextual factors might affect the treatment effects, we have not tried to investigate the reasons for inconsistency within this review given the small amount of studies available per comparison. Except for the factor ‘CDS combined with patient-oriented strategies versus CDS only’, we explored variation in summary estimates based on the type of targeted behaviour.

We described the results as ‘small’, ‘modest’, ‘moderate’, and ‘large’ like this was done in key systematic reviews about implementation strategies [24]. While the terms modest and moderate can convey the sense of being not important, it might be unrealistic to expect magic bullets. Therefore, we should not discard the modest or moderate improvements that some features can bring on population level and for individual patients [39].

Our reporting of the median RD across the included studies does not take the precision of the study effect into account. We did not report confidence intervals of the reported effect sizes, and we do not know if these intervals overlapped with ‘no effect’. However, it was not possible to use conventional meta-analysis methods and the median effect approach that we implemented has been used in many Cochrane reviews. Interrupted time series also form a robust study design but these require a different type of analysis. In a future review, we could include interrupted time series and analyse them separately.

The effectiveness of CDS may be affected by many other factors that are not assessed in this review. Some of these have been studied in contexts other than CDS. Brandt et al. compared the presentation of guideline recommendations in a digitally structured format with standard formats and showed that optimised guideline presentation formats can potentially lead to higher adherence [40]. Other factors do not lend themselves to evaluations within a trial and therefore process evaluations or syntheses of qualitative research may help to answer questions on stakeholders’ perceptions and experiences regarding the use of CDS. The GUIDES project has synthesised the information from the best current evidence, and we hope that the development of the CDS implementation checklist will help teams to increase the successful use of CDS [17].

### Further research

Despite the large number of head-to-head trials, further trials with direct comparisons for a range of CDS intervention factors are needed to draw firm conclusions on how to improve the effectiveness of CDS. To reduce baseline differences between trial arms and prevent contamination across trial arms, studies should use appropriate randomisation whenever possible. Consideration of unit of analysis errors is important for cluster RCTs [23]. Economic evaluations and measurements of satisfaction should also be incorporated in trials. A seldom reported measure related to provider satisfaction would be whether the CDS is time-saving. Studies where CDS users could make an informed choice on which outcomes they want to improve, and CDS that assists in achieving those outcomes are warranted.

## Conclusions

Stakeholders need to be informed about how best to employ CDS in ways that improve (1) healthcare, (2) health outcomes, (3) cost management, and (4) patient and provider satisfaction [9, 10]. To do this, it is important that stakeholders understand how to enable effective CDS. The findings of this review suggest that multiple factors may affect the success of CDS interventions. CDS may be more effective when the advice is provided automatically and displayed on-screen and when the suggestions are more patient-specific. CDS interventions combined with professional-oriented strategies, combined with patient-oriented strategies, or combined with staff-oriented strategies probably also improves adherence. Providing CDS directly to patients may also positively affect adherence. The effects of the different factors may vary with how the intervention is set up and carried out.

The certainty of the evidence was low to moderate for all the factors. This review provides useful insights about how to increase the effectiveness of CDS, but it is important to be aware of the uncertainty of these results.

## Additional files

Additional file 1: Search string. (DOCX 12 kb)

Additional file 2: Study characteristics. (DOCX 178 kb)

Additional file 3: Study results. (DOCX 95 kb)

Additional file 4: Synthesis of results per factor. (DOCX 240 kb)

## Abbreviations

CDS: Computerised decision support
CI: Confidence interval
EPOC: Effective Practice and Organisation of Care
GUIDES: GUideline Implementation with Decision Support
IQR: Interquartile range
OR: Odds ratio
RD: Risk difference
