# Supplementary material for: A systematic review of trials evaluating success factors of interventions with computerised clinical decision support
Source: Implement Sci. 2018 Aug 20;13:114. doi: 10.1186/s13012-018-0790-1 (PMC6102833; doi:10.1186/s13012-018-0790-1)
Supplement: Supplementary file 1 — Search string. (DOCX 12 kb) [file 13012_2018_790_MOESM1_ESM.docx]

# Additional file 1

# Search string in Medline (Ovid)

1. exp "Appointments and Schedules"/

2. "Forms and Records Control"/

3. Medical Records Systems, Computerized/

4. exp Decision Making, Computer-Assisted/

5. exp Artificial Intelligence/

6. Decision Support Systems, Clinical/

7. Reminder Systems/

8. feedback/

9. Decision Making/ and exp Computers/

10. 1 or 2 or 3 or 4 or 5 or 6 or 7 or 8 or 9

11. (remind$ or sticker$ or (decision adj support) or alert$ or (flowsheet$ or flow sheet$ or flowchart$ or flow chart$) or (order adj1 (form$ or sheet$)) or ((request or encounter) adj1 form$) or checklist$ or ((tag or tagged or sticker or annot$) adj5 (note$ or record$ or sheet$ or chart$ or form$)) or (computer$ adj4 feedback) or (computer$ adj4 suggestion$) or (computer$ adj4 message$) or (computer$ adj4 order) or (electronic adj4 feedback) or (electronic adj4 suggestion$) or (electronic adj4 message$) or (information adj4 feedback) or prompt$).tw.

12. 10 and 11

13. (computer$ adj2 reminder$).tw.

14. 12 or 13

15. randomized controlled trial.pt.

16. controlled clinical trial.pt.

17. randomized controlled trials/

18. random allocation/

19. double blind method/

20. single blind method/

21. clinical trial.pt.

22. exp Clinical Trial/

23. (clinical adj trial?).tw.

24. ((singl$ or doubl$ or trebl$ or tripl$) adj25 (blind$ or mask$)).tw.

25. (random$ or placebo?).tw.

26. 15 or 16 or 17 or 18 or 19 or 20 or 21 or 22 or 23 or 24 or 25

27. animal/

28. human/

29. 27 not (27 and 28)

30. 26 not 29

31. 14 and 30
