# Supplementary material for: A systematic review of trials evaluating success factors of interventions with computerised clinical decision support
Source: Implement Sci. 2018 Aug 20;13:114. doi: 10.1186/s13012-018-0790-1 (PMC6102833; doi:10.1186/s13012-018-0790-1)
Supplement: Supplementary file 2 — Study characteristics. (DOCX 178 kb) [file 13012_2018_790_MOESM2_ESM.docx]

# Additional file 2

# Characteristics of the included studies

Arts 2017

| Methods | Cluster RCT, unit of allocation: practice  Summary risk of bias: unclear risk | |
| --- | --- | --- |
| Participants | Patients in the included study arms: patients with atrial fibrillation ; female: 50%; mean age: 73; n= 522 analysed  Professionals: 14 practices with on average 2.1 general practitioners per practice received the included interventions  Setting: outpatient, the Netherlands | |
| Interventions | Arm 1: CDS that required override reasons, directed at the healthcare provider and delivered on screen  Arm 2: standard CDS | |
| Outcomes | Dichotomous process measures: % adherence to the CDS advice  Follow up period: 9 months | |
| Notes | Data for control group not used; data for the intervention arms were not reported in the paper, but were extracted from the supplemental data set | |
| ***Risk of bias*** |  |  |
| **Bias** | **Authors’ judgement** | **Support for judgement** |
| Random sequence generation | Low risk | Random number generator |
| Allocation concealment | Unclear risk | Concealment of allocation is not clear |
| Similar outcomes at baseline | Unclear risk | Baseline outcome data per intervention arm are not clear |
| Similar characteristics at baseline | Unclear risk | Baseline characteristics per intervention arm are not clear |
| Incomplete outcome data | Low risk | No loss to follow up at practice level |
| Blinding of outcome assessment | Low risk | Objective outcomes |
| Adequate protection against contamination | Low risk | Allocation of practices |
| Selective reporting | Low risk | Protocol registered, outcome data per intervention arm not reported, but data set was published as a supplement |
| Other bias | Low risk |  |

Bates 1998

| Methods | Cluster RCT, unit of allocation: care unit  Summary risk of bias: high risk | |
| --- | --- | --- |
| Participants | Patients in the included study arms: patients admitted at an intensive or general care unit; female: 54.3%; mean age: 53.2; n= 4220 patient admissions with 24539 patient days analysed  Professionals: healthcare providers from 2 intensive care units and 6 general care units  Setting: inpatient, United States | |
| Interventions | Arm 1: standard CDS directed at the healthcare provider and delivered on screen + multicomponent team intervention Arm 2: standard CDS | |
| Outcomes | Continuous process measures: rate of nonintercepted serious medication errors per 100 patient days  Follow up period: 9 months | |
| Notes |  | |
| ***Risk of bias*** |  |  |
| **Bias** | **Authors’ judgement** | **Support for judgement** |
| Random sequence generation | Low risk | Random number generator |
| Allocation concealment | Low risk | Allocation by practice unit |
| Similar outcomes at baseline | Unclear risk | Not clear |
| Similar characteristics at baseline | Unclear risk | Not clear |
| Incomplete outcome data | High risk | The study was conducted at 2 hospitals, but only data from one study are included because of inadequate data collection in the other hospital |
| Blinding of outcome assessment | Low risk | Blinded outcome assessment |
| Adequate protection against contamination | High risk | Cross-over is reported |
| Selective reporting | Unclear risk | No protocol identified |
| Other bias | Low risk |  |

Becker 1990

| Methods | RCT , unit of allocation: patients  Summary risk of bias: High risk | |
| --- | --- | --- |
| Participants | Patients in the included study arms: patients with preventive care needs; female: 70.1%; mean age: 51.4; n= 371 analysed  Professionals: number of physicians not clear  Setting: outpatient, United States | |
| Interventions | Arm 1: CDS directed at physicians (on paper) and at patients (letter)  Arm 2: CDS directed at physicians only | |
| Outcomes | Dichotomous process measures: % compliance with preventive care guidelines  Follow up period: ≥ 4 months | |
| Notes |  | |
| ***Risk of bias*** |  |  |
| **Bias** | **Authors’ judgement** | **Support for judgement** |
| Random sequence generation | Unclear risk | Method not clear |
| Allocation concealment | Unclear risk | Not clear |
| Similar outcomes at baseline | Low risk | Not clear for the outcome, but no important differences for number of preventive care needs (proxy) |
| Similar characteristics at baseline | Low risk | No important differences |
| Incomplete outcome data | High risk | Important loss to follow up, it is not clear if the reasons are balanced |
| Blinding of outcome assessment | Low risk | Objective outcomes |
| Adequate protection against contamination | High risk | Physicians treated patients from multiple arms |
| Selective reporting | Unclear risk | No protocol identified |
| Other bias | Low risk |  |

Bloomfield 2005

| Methods | Cluster RCT, unit of allocation: healthcare provider  Summary risk of bias: unclear risk | |
| --- | --- | --- |
| Participants | Patients in the included study arms: patients with ischemic heart disease; female: x%; mean age: x; n= 1349 patients (including one additional trial arm)  Professionals: 92 primary care physicians at 5 intervention sites received the randomised interventions  Setting: outpatient, United States | |
| Interventions | Arm 1: CDS (progress notes) directed at the healthcare provider and delivered on screen some days before the visit Arm 2: CDS (reminders) directed at the healthcare provider and delivered on screen during the visit | |
| Outcomes | Dichotomous process measures: % patients with prescription of lipid lowering drugs  Follow up period: 12 months | |
| Notes | We did not extract data for the trial arms because the patient directed information was not computer generated. | |
| ***Risk of bias*** |  |  |
| **Bias** | **Authors’ judgement** | **Support for judgement** |
| Random sequence generation | Low risk | Balanced block randomisation scheme |
| Allocation concealment | Unclear risk | Not clear |
| Similar outcomes at baseline | Low risk | Patient population limited to those without current prescriptions of lipid lowering drugs |
| Similar characteristics at baseline | Unclear risk | Not clear |
| Incomplete outcome data | Unclear risk | 21 out of 92 physicians did not complete the study, it is not clear if this was balanced |
| Blinding of outcome assessment | Low risk | Objective outcomes |
| Adequate protection against contamination | Unclear risk | Physicians collaborated in the same intervention sites, it is unclear if this lead to contamination |
| Selective reporting | Unclear risk | No protocol identified |
| Other bias | Low risk |  |

Bosworth 2011

| Methods | RCT, unit of allocation: patients  Summary risk of bias: high risk | |
| --- | --- | --- |
| Participants | Patients in the included study arms: hypertension patients with inadequate blood pressure control; female: 10.5%; mean age: 63.5; n= 296 analysed in the included arms  Professionals: 3 primary care clinics with 28 internal medicine faculty physicians and 10 midlevel providers received the intervention  Setting: outpatient, United States | |
| Interventions | Arm 1: CDS directed at the healthcare provider and provided on screen supplemented with a tailored health behaviour programme directed at patients  Arm 2: standard CDS | |
| Outcomes | Dichotomous patient measures: % with blood pressure in control (primary outcome)  Continuous patient measures: mean systolic blood pressure, mean diastolic blood pressure  Follow up period: 18 months | |
| Notes | Data from other trial arms not extracted; some data extracted from a graphic; data on mean diastolic blood pressure are not available per trial arm | |
| ***Risk of bias*** |  |  |
| **Bias** | **Authors’ judgement** | **Support for judgement** |
| Random sequence generation | Unclear risk | Randomisation method is not clear |
| Allocation concealment | Unclear risk | consecutively numbered envelopes, but process to monitor preservation of concealment is unclear |
| Similar outcomes at baseline | High risk | Important differences for the primary outcome |
| Similar characteristics at baseline | High risk | Important differences |
| Incomplete outcome data | Low risk | Minimal loss to follow up |
| Blinding of outcome assessment | Low risk | Objective outcomes |
| Adequate protection against contamination | Unclear risk | Not clear |
| Selective reporting | Low risk | Protocol registered and no selective reporting identified |
| Other bias | Low risk |  |

Burack 1996

| Methods | RCT, unit of allocation: patient  Summary risk of bias: high risk | |
| --- | --- | --- |
| Participants | Patients in the included study arms: patients due for breast cancer screening; female: 100%; mean age: not clear; n= 1627 analysed in all the arms  Professionals: 20 physicians from 2 sites  Setting: outpatient, United States | |
| Interventions | Arm 1: CDS directed at the patient and delivered by letter  Arm 2: CDS directed at both the healthcare provider and the patient  Arm 3: CDS directed at the healthcare provider and delivered on paper | |
| Outcomes | Dichotomous process measures: % women with mammography (primary outcome), % women with visit to a physician  Continuous process measures: time to visit to a physician  Follow up period: 12 months | |
| Notes | Data are reported and extracted separately for two sites; Data on control group not extracted | |
| ***Risk of bias*** |  |  |
| **Bias** | **Authors’ judgement** | **Support for judgement** |
| Random sequence generation | Unclear risk | Randomisation method is not clear |
| Allocation concealment | Unclear risk | Not clear |
| Similar outcomes at baseline | Low risk | No important differences |
| Similar characteristics at baseline | Low risk | No important differences |
| Incomplete outcome data | Unclear risk | Not clear |
| Blinding of outcome assessment | Low risk | Objective outcomes |
| Adequate protection against contamination | High risk | Allocation per patient and healthcare providers met patients from multiple arms |
| Selective reporting | Unclear risk | No protocol identified |
| Other bias | Low risk |  |

Burack 1998

| Methods | RCT, unit of allocation: patient  Summary risk of bias: high risk | |
| --- | --- | --- |
| Participants | Patients in the included study arms: women due for cervical cancer screening; female: 100%; mean age: range 18-39; n= 3848 analysed in all the arms  Professionals: 20 primary care physicians from 3 sites received the interventions  Setting: outpatient, United States | |
| Interventions | Arm 1: CDS directed at the patient and delivered by letter  Arm 2: CDS directed at both the healthcare provider and the patient  Arm 3: CDS directed at the healthcare provider and delivered on paper | |
| Outcomes | Dichotomous process measures: % patients with pap smear test (primary outcome), % patients with visits  Continuous process measures:  Economic measures: % patients with visits  Follow up period: 12 months | |
| Notes | Data on control group not extracted | |
| ***Risk of bias*** |  |  |
| **Bias** | **Authors’ judgement** | **Support for judgement** |
| Random sequence generation | Unclear risk | Randomisation method not described |
| Allocation concealment | Unclear risk | Not clear |
| Similar outcomes at baseline | Unclear risk | Not clear |
| Similar characteristics at baseline | Unclear risk | The baseline data per arm is not clear |
| Incomplete outcome data | Unclear risk | Not clear |
| Blinding of outcome assessment | Low risk | Objective outcomes |
| Adequate protection against contamination | High risk | Allocation by patient and healthcare providers met patients from multiple arms |
| Selective reporting | Unclear risk | No protocol identified |
| Other bias | Low risk |  |

Carroll 2013

| Methods | RCT, unit of allocation: patient  Summary risk of bias: high risk | |
| --- | --- | --- |
| Participants | Patients in the included study arms: mothers at risk of depression; female: 100%; mean age: not reported; n= 3520 analysed  Professionals: 48 paediatricians received the interventions  Setting: outpatient, United States | |
| Interventions | Arm 1: Completion of screening forms by patients, followed by CDS message (directed to the healthcare provider and delivered on paper) tailored to the patient’s risk of depression, combined with hand-outs for the patient provided by the physician  Arm 2: The same intervention as in arm 1 without patient hand-outs  Arm 3: Standard CDS message for the healthcare provider without screening forms for patients | |
| Outcomes | Dichotomous process measures: % mothers with suspected maternal depression and therefore referred (primary outcome)  Dichotomous patient measures: % mothers screened positive for depressed mood; % mothers screened positive for anhedonia  Follow up period: 20 months | |
| Notes |  | |
| ***Risk of bias*** |  |  |
| **Bias** | **Authors’ judgement** | **Support for judgement** |
| Random sequence generation | Low risk | Computer generated random numbers |
| Allocation concealment | Low risk | Allocation by a computer |
| Similar outcomes at baseline | Unclear risk | Not clear |
| Similar characteristics at baseline | Unclear risk | Insufficient data provided |
| Incomplete outcome data | Unclear risk | The amount of loss to follow-up due to not recording the outcome data is not clear |
| Blinding of outcome assessment | Low risk | Objective outcomes |
| Adequate protection against contamination | High risk | Allocation by patient and the healthcare providers were exposed to all the interventions with evidence of contamination |
| Selective reporting | Low risk | Protocol was registered and no selective reporting identified |
| Other bias | Low risk |  |

Chambers 1991

| Methods | Cluster RCT, unit of allocation: provider  Summary risk of bias: high risk | |
| --- | --- | --- |
| Participants | Patients in the included study arms: elderly patients or patients at risk targeted for influenza vaccination; female: 74%; mean age: not reported; n= 447 analysed in the included arms  Professionals: 12 fulltime faculty and 18 residents received the interventions  Setting: outpatient, United States | |
| Interventions | Arm 1: CDS message is generated for all the eligible patients, directed to the healthcare provider and delivered on paper Arm 2: CDS message is generated for half of the eligible patients | |
| Outcomes | Dichotomous process measures: % patients with influenza vaccination  Follow up period: 2 months | |
| Notes | Source of funding is not clear; data for the control group not extracted | |
| ***Risk of bias*** |  |  |
| **Bias** | **Authors’ judgement** | **Support for judgement** |
| Random sequence generation | Low risk | Computerised randomisation programme |
| Allocation concealment | Low risk | Allocation by provider at the start of the trial |
| Similar outcomes at baseline | Unclear risk | Not clear |
| Similar characteristics at baseline | High risk | Important differences present |
| Incomplete outcome data | Low risk | Loss to follow-up is minimal |
| Blinding of outcome assessment | Low risk | Objective outcome |
| Adequate protection against contamination | Low risk |  |
| Selective reporting | Unclear risk | No protocol identified |
| Other bias | Low risk |  |

Christakis 2004

| Methods | RCT, unit of allocation: Patient  Summary risk of bias: high risk | |
| --- | --- | --- |
| Participants | Patients in the included study arms: pediatric patients with a low continuity of care index; female: 53%; mean age: 8.5; n= 309 analysed in the included arms  Professionals: 2 pediatricians, 2 nurse practitioners and residents  Setting: outpatient, United States | |
| Interventions | Arm 1: CDS directed at both the scheduler and the healthcare provider  Arm 2: CDS directed at the scheduler; delivered on screen  Arm 3: CDS directed at the healthcare provider | |
| Outcomes | Continuous process measures: Continuity of care score  Follow up period: 9 months | |
| Notes | We judged that the comparison of arm 2 versus arm 3 was not relevant for our review | |
| ***Risk of bias*** |  |  |
| **Bias** | **Authors’ judgement** | **Support for judgement** |
| Random sequence generation | Low risk | Computer randomisation |
| Allocation concealment | Low risk | Randomisation at the start of the study by on site computer |
| Similar outcomes at baseline | Low risk | No important differences |
| Similar characteristics at baseline | Low risk | No important differences |
| Incomplete outcome data | Unclear risk | Important loss to follow up and not clear if the reasons are balanced |
| Blinding of outcome assessment | Low risk | Objective outcome |
| Adequate protection against contamination | High risk | Allocation by patients and the professionals treated patients from different arms |
| Selective reporting | Unclear risk | No protocol identified |
| Other bias | Low risk |  |

Del Fiol 2008

| Methods | Cluster RCT , unit of allocation: healthcare provider  Summary risk of bias: unclear risk | |
| --- | --- | --- |
| Participants | Patients in the included study arms: no data reported  Professionals: 104 healthcare providers received the interventions, 80 providers in the included study arms at follow-up  Setting: outpatient and inpatient; United States | |
| Interventions | Arm 1: infobutton links for the healthcare provider that direct to specific content topics (available on screen)  Arm 2: infobutton links that direct to general overview content | |
| Outcomes | Dichotomous process measures: % of infobutton searches where the needed information was found  Continuous process measures: score for the impact of the information seeking; number of infobutton searches, amount of time spent seeking information  Follow up period: 7 months | |
| Notes |  | |
| ***Risk of bias*** |  |  |
| **Bias** | **Authors’ judgement** | **Support for judgement** |
| Random sequence generation | Low risk | A computerised random number generator was used (additional information provided by the authors) |
| Allocation concealment | Unclear risk | Not clear |
| Similar outcomes at baseline | Low risk | Participants were paired on two baseline outcome measures |
| Similar characteristics at baseline | Unclear risk | Not clear |
| Incomplete outcome data | Unclear risk | Important loss to follow-up, and it is not clear if the reasons were balanced |
| Blinding of outcome assessment | Low risk | Outcome assessors were blinded, time outcomes were extracted from a computer log (additional information provided by the authors) |
| Adequate protection against contamination | Low risk | Contamination is unlikely |
| Selective reporting | Unclear risk | No protocol identified |
| Other bias | Low risk |  |

Denig 2014

| Methods | Cluster RCT, unit of allocation: practice and patient  Summary risk of bias: high risk | |
| --- | --- | --- |
| Participants | Patients in the included study arms: diabetes patients; female: 42%; mean age: 61.8; n= 225 analysed  Professionals: 25 general practitioners and practice nurses received the interventions (including control group)  Setting: outpatient, the Netherlands | |
| Interventions | Arm 1: Long patient-directed CDS information showing treatment effects for 5 outcomes and delivered on screen  Arm 2: Long patient-directed CDS information showing treatment effects for 5 outcomes and delivered on paper  Arm 3: Short patient-directed CDS information showing treatment effects for 1 outcome and delivered on screen  Arm 4: Short patient-directed CDS information showing treatment effects for 1 outcome and delivered on paper  The healthcare professional was the intermediate to provide and discuss the decision support with the patient. | |
| Outcomes | Dichotomous process measures: % patients with intensified glucose treatment; % patients with intensified blood pressure treatment; % patients with intensified lipid treatment; % patients with RAS inhibitors prescribed  Continuous patient measures: Score on diabetes empowerment scale (primary outcome)  Follow up period: 6 months | |
| Notes | Data recalculated from the odds | |
| ***Risk of bias*** |  |  |
| **Bias** | **Authors’ judgement** | **Support for judgement** |
| Random sequence generation | Low risk | computer generated random allocation sequence |
| Allocation concealment | Low risk | concealed allocation |
| Similar outcomes at baseline | High risk | The baseline outcomes are not reported per intervention arm |
| Similar characteristics at baseline | Unclear risk | The baseline characteristics are not reported per intervention arm |
| Incomplete outcome data | Low risk | Loss to follow-up is limited and balanced across groups |
| Blinding of outcome assessment | High risk | The primary outcome is subjective and self-assessed by the patients |
| Adequate protection against contamination | High risk | Physicians treated patients from both the short and the long CDS information arms |
| Selective reporting | High risk | Protocol was published and some planned secondary outcomes were not reported |
| Other bias | Low risk |  |

Derose 2005

| Methods | RCT, unit of allocation: patient  Summary risk of bias: high risk | |
| --- | --- | --- |
| Participants | Patients: patients with a high cardiovascular risk; female: 39.2 to 54.5%; mean age: 64.9 to 68.2; n= 8861 analysed  Professionals: 1089 primary care and specialty physicians received the interventions  Setting: outpatient, United States | |
| Interventions | Arm 1: CDS presenting patient data, treatment data and two new study specific recommendations; the CDS is provided on paper on the morning of a scheduled consultation and directed at the healthcare provider  Arm 2: the same CDS with recommendations that are not related to the study | |
| Outcomes | Dichotomous process measures: % with dispensed prescriptions of an ACEI or ARB and a statin within two weeks after a visit  Follow up period: 1-1.5 months | |
| Notes | Commercial funding | |
| ***Risk of bias*** |  |  |
| **Bias** | **Authors’ judgement** | **Support for judgement** |
| Random sequence generation | Low risk | Computer-based randomisation process |
| Allocation concealment | Low risk | Randomisation by a computer |
| Similar outcomes at baseline | Unclear risk | Not clear |
| Similar characteristics at baseline | Low risk | No important differences |
| Incomplete outcome data | Unclear risk | Not clear |
| Blinding of outcome assessment | Low risk | Objective outcomes |
| Adequate protection against contamination | High risk | Healthcare professionals treated patients from both arms |
| Selective reporting | Unclear risk | No protocol identified |
| Other bias | Low risk |  |

Dexter 2004

| Methods | Cluster RCT, unit of allocation: Physician teams  Summary risk of bias: high risk | |
| --- | --- | --- |
| Participants | Patients in the included study arms: patients discharged from the hospital that are eligible for vaccination; female: 52%; mean age: 53; n= 1314 analysed  Professionals: 8 teams including 212 physicians received the interventions  Setting: inpatient, United States | |
| Interventions | Arm 1: CDS (standing orders) directed at nurses and delivered on screen  Arm 2: CDS (reminders) directed at physicians and delivered on screen | |
| Outcomes | Dichotomous process measures: % patients with pneumococcal vaccinations administered, % patients with influenza vaccinations administered  Follow up period: 14 months | |
| Notes | The outcomes on patients with vaccinations ordered spontaneously were not used because the data comes from a patient population that is not included in the trial | |
| ***Risk of bias*** |  |  |
| **Bias** | **Authors’ judgement** | **Support for judgement** |
| Random sequence generation | Low risk | Randomisation by coin flip |
| Allocation concealment | Low risk | Person doing the randomisation was blinded |
| Similar outcomes at baseline | Low risk | All the patients required vaccination |
| Similar characteristics at baseline | Unclear risk | Authors report that there were no important differences, but no data are presented |
| Incomplete outcome data | Unclear risk | Not clear if there was loss to follow up for the primary outcome |
| Blinding of outcome assessment | Low risk | Objective outcomes |
| Adequate protection against contamination | High risk | Important cross over of physicians |
| Selective reporting | Unclear risk | No protocol identified, adverse reactions were collected but not reported |
| Other bias | Low risk |  |

Dickinson 1981

| Methods | Cluster RCT, unit of allocation: practice  Summary risk of bias: high risk | |
| --- | --- | --- |
| Participants | Patients in the included study arms: patients with hypertension; female: 68.5%; mean age: 49.5; n= 139 analysed in the included arms  Professionals: 4 clinical teams with 37 residents and 4 faculty physicians received the interventions  Setting: outpatient, United States | |
| Interventions | Arm 1: CDS (monthly computer feedback) directed at the healthcare professional and delivered on paper combined with physician education  Arm 2: standard CDS | |
| Outcomes | Dichotomous patient measures: % patients with controlled diastolic BP, % patients improved  Continuous patient measures: mean diastolic blood pressure, mean systolic blood pressure  Economic measures: mean appointments per patient  Follow-up period: 7 months | |
| Notes | Data from other trial arms were not extracted; data on physician knowledge not extracted | |
| ***Risk of bias*** |  |  |
| **Bias** | **Authors’ judgement** | **Support for judgement** |
| Random sequence generation | Unclear risk | Randomisation method not clear |
| Allocation concealment | Unclear risk | Allocation concealment not clear |
| Similar outcomes at baseline | High risk | Important differences |
| Similar characteristics at baseline | High risk | Important differences |
| Incomplete outcome data | Low risk | No loss to follow up |
| Blinding of outcome assessment | Low risk | Objective outcomes |
| Adequate protection against contamination | Low risk | Allocation per practice |
| Selective reporting | Unclear risk | No protocol identified |
| Other bias | Low risk |  |

Duke 2013

| Methods | Cluster RCT, unit of allocation: healthcare provider  Summary risk of bias: low risk | |
| --- | --- | --- |
| Participants | Patients in the included study arms: focus on drug-drug interactions associated with hyperkalemia; no data reported on patient numbers; 2140 CDS messages triggered  Professionals: 203 physicians received the interventions  Setting: outpatient, United States | |
| Interventions | Arm 1: CDS (drug-drug interactions) supplemented with specific patient laboratory data; directed at the healthcare professionals and delivered on screen  Arm 2: standard CDS | |
| Outcomes | Dichotomous process measures: % adherence with CDS messages  Follow up period: 6 months | |
| Notes | Data on adverse events (hyperkalemia and hospital admissions) were not available per comparison group and not extracted | |
| ***Risk of bias*** |  |  |
| **Bias** | **Authors’ judgement** | **Support for judgement** |
| Random sequence generation | Low risk | Random number generator |
| Allocation concealment | Low risk | Allocation by professional and done at the start of the study |
| Similar outcomes at baseline | Unclear risk | Not clear |
| Similar characteristics at baseline | Unclear risk | Not clear |
| Incomplete outcome data | Low risk | Data automatically captured |
| Blinding of outcome assessment | Low risk | Objective outcomes |
| Adequate protection against contamination | Unclear risk | Not clear |
| Selective reporting | Low risk |  |
| Other bias | Low risk |  |

El-Kareh 2011

| Methods | Cluster non randomised controlled trial, unit of allocation: practice  Summary risk of bias: high risk | |
| --- | --- | --- |
| Participants | Patients in the included study arms: patients with due preventive procedures; female: % not clear; mean age: not clear; n= ≥7351 analysed  Professionals: 8 primary care practices  Setting: outpatient, United States | |
| Interventions | Arm 1: CDS (on screen) directed at the healthcare provider that is linked to order entry modules  Arm 2: standard CDS | |
| Outcomes | Dichotomous process measures: % of overdue tests performed following CDS  Follow up period: 6 months | |
| Notes |  | |
| ***Risk of bias*** |  |  |
| **Bias** | **Authors’ judgement** | **Support for judgement** |
| Random sequence generation | High risk | Non randomised controlled trial |
| Allocation concealment | High risk | Non randomised controlled trial |
| Similar outcomes at baseline | High risk | Important differences present |
| Similar characteristics at baseline | Unclear risk | Not clear |
| Incomplete outcome data | Low risk | Data coming from an electronic central data repository |
| Blinding of outcome assessment | Low risk | Objective outcomes |
| Adequate protection against contamination | Low risk | Allocation by practice |
| Selective reporting | Unclear risk | No protocol identified |
| Other bias | Low risk |  |

Feldman 2005

| Methods | Cluster RCT, unit of allocation: provider  Summary risk of bias: high risk | |
| --- | --- | --- |
| Participants | Patients in the included study arms: patients admitted to a home care agency with a primary diagnosis of heart failure; female: 65.1%; mean age: 72.1; n= 401 included in the analysis  Professionals: 388 nurses received the interventions  Setting: outpatient, United States | |
| Interventions | Arm 1: CDS combined with a multicomponent strategy (CDS directed at the healthcare professional and delivered on screen)  Arm 2: Standard CDS | |
| Outcomes | Dichotomous patient measures: % with any hospitalisation, % with any emergency department visits, % with any outpatient doctor visit  Continuous patient measures: number of home care related visits, number of inpatient nights, number of emergency department visits, number of outpatient doctor visits, score on cardiomyopathy scale, score on depression scale, score on health related quality of life scale  Economic measures: Home care-related costs, Overall costs, Cost to produce a 5% improvement on cardiomyopathy scale  Follow up period: 1,5 months | |
| Notes | Data on control group not extracted; data on self-management behaviours not extracted | |
| ***Risk of bias*** |  |  |
| **Bias** | **Authors’ judgement** | **Support for judgement** |
| Random sequence generation | Low risk | Computerised randomisation system |
| Allocation concealment | Low risk | Computerised randomisation system |
| Similar outcomes at baseline | Unclear risk | Not clear |
| Similar characteristics at baseline | High risk | Important differences |
| Incomplete outcome data | Unclear risk | Important loss to follow up, but balanced across the groups with reasons known |
| Blinding of outcome assessment | Low risk | Blinded assessment |
| Adequate protection against contamination | Unclear risk | Frequent interactions between healthcare providers from different arms |
| Selective reporting | Unclear risk | No protocol identified |
| Other bias | Low risk |  |

Feldstein 2006a

| Methods | RCT, unit of allocation: patient  Summary risk of bias: high risk | |
| --- | --- | --- |
| Participants | Patients in the included study arms: women at risk of having osteoporosis; female: 100%; mean age: 73; n= 311 analysed  Professionals: 159 primary care providers from 15 primary care clinics participated in the trial  Setting: outpatient, United States | |
| Interventions | Arm 1: CDS directed at the healthcare provider (on screen) and letter directed at the patient (on paper)  Arm 2: CDS directed at the healthcare provider | |
| Outcomes | Dichotomous process measures: % patients that received a pharmacological treatment or bone mineral density measurement (primary outcome)  Continuous patient measure: calcium intake mg/day  Follow up period: 6 months | |
| Notes | Study with commercial funding; data from the control group was not used | |
| ***Risk of bias*** |  |  |
| **Bias** | **Authors’ judgement** | **Support for judgement** |
| Random sequence generation | Low risk | Random number generator |
| Allocation concealment | Low risk | Allocation by study statistician at the start of the study |
| Similar outcomes at baseline | Low risk | No patients had a bone scan or osteoporosis medication, no important differences for calcium intake |
| Similar characteristics at baseline | Low risk | No important differences |
| Incomplete outcome data | Low risk | Limited loss to follow up |
| Blinding of outcome assessment | Low risk | Objective outcomes |
| Adequate protection against contamination | High risk | Allocation was by patient and healthcare providers treated patients that belonged to both groups |
| Selective reporting | Unclear risk | No protocol identified |
| Other bias | Low risk |  |

Feldstein 2006b

| Methods | Cluster RCT, unit of allocation: Institution  Summary risk of bias: high risk | |
| --- | --- | --- |
| Participants | Patients in the included study arms: patients using warfarin; female: 52%; mean age: 70; n= 9910 analysed  Professionals: clinicians from 15 primary care clinics  Setting: outpatient, United States | |
| Interventions | Arm 1: CDS directed at healthcare providers and delivered on screen, combined with academic detailing  Arm 2: standard CDS | |
| Outcomes | Continuous process measures: Number of co-prescriptions of warfarin-interacting medications/10000 warfarin users/ month  Follow up period: 21 months | |
| Notes |  | |
| ***Risk of bias*** |  |  |
| **Bias** | **Authors’ judgement** | **Support for judgement** |
| Random sequence generation | Low risk | Random number generator |
| Allocation concealment | Low risk | Allocation by a study statistician |
| Similar outcomes at baseline | High risk | Important differences |
| Similar characteristics at baseline | Low risk | No important differences |
| Incomplete outcome data | Low risk | Limited loss to follow up |
| Blinding of outcome assessment | Low risk | Objective outcomes and blinded assessment |
| Adequate protection against contamination | Low risk | Allocation by institution |
| Selective reporting | Unclear risk | No protocol identified |
| Other bias | Low risk |  |

Feldstein 2006c

| Methods | Cluster RCT, unit of allocation: clinic  Summary risk of bias: low risk | |
| --- | --- | --- |
| Participants | Patients in the included study arms: patients with due laboratory monitoring after medication prescription; female: 53%; mean age: 59.1; n= 120 analysed  Professionals: 7 primary care clinics with 88 healthcare professionals received the interventions for the included arms  Setting: outpatient, United States | |
| Interventions | Arm 1: CDS directed at the patient and delivered by telephone  Arm 2: CDS directed at the healthcare provider and delivered on screen | |
| Outcomes | Dichotomous process measures: % patients with completed baseline laboratory monitoring (primary outcome)  Dichotomous patient measures: % of patients with abnormal test results  Follow up period: 25 days | |
| Notes | Data for the other trial arm not extracted | |
| ***Risk of bias*** |  |  |
| **Bias** | **Authors’ judgement** | **Support for judgement** |
| Random sequence generation | Low risk | Computerised random number generator |
| Allocation concealment | Low risk | Clinics randomised at start |
| Similar outcomes at baseline | Low risk | No important differences |
| Similar characteristics at baseline | Low risk | No important differences |
| Incomplete outcome data | Low risk | No loss to follow-up |
| Blinding of outcome assessment | Low risk | Blinded assessment |
| Adequate protection against contamination | Low risk | Allocation by clinic |
| Selective reporting | Low risk | Protocol registered and no selective reporting identified |
| Other bias | Low risk |  |

Folks 2011

| Methods | Cluster RCT, unit of allocation: practice  Summary risk of bias: high risk | |
| --- | --- | --- |
| Participants | Patients in the included study arms: patients with a once daily dosing of aspirin (or clopidogrel) or/and a diagnosis of cardiovascular disease; female: % not clear; mean age: not clear; n= 3409 analysed for the included arms  Professionals: primary care physicians from 5 practices  Setting: outpatient, United States | |
| Interventions | Arm 1: CDS directed at the healthcare provider (delivered on screen) combined with academic detailing, supplemented with CDS directed at patient (delivered on paper)  Arm 2: CDS directed at the healthcare provider combined with academic detailing | |
| Outcomes | Dichotomous process measures: % of patients without cardiovascular disease that receive aspirin  Follow up period: 12 months | |
| Notes | Source of funding is not clear; Data on control group and academic detailing only group are not extracted | |
| ***Risk of bias*** |  |  |
| **Bias** | **Authors’ judgement** | **Support for judgement** |
| Random sequence generation | Unclear risk | Not clear |
| Allocation concealment | Unclear risk | Not clear |
| Similar outcomes at baseline | High risk | Important differences |
| Similar characteristics at baseline | Unclear risk | Not clear |
| Incomplete outcome data | Unclear risk | Not clear |
| Blinding of outcome assessment | Low risk | Objective outcomes |
| Adequate protection against contamination | Low risk | Allocation by practice |
| Selective reporting | Low risk | Protocol registered and no selective reporting identified |
| Other bias | Low risk |  |

Forrest 2013

| Methods | Cluster RCT, unit of allocation: practice  Summary risk of bias: high risk | |
| --- | --- | --- |
| Participants | Patients in the included study arms: children with otitis media; female: 46.7%; mean age: not clear; n= 139305 visits by 55779 children in all the study arms  Professionals: 122 paediatricians from 16 practices received the interventions included in the analysis  Setting: outpatient, United States | |
| Interventions | Arm 1: CDS combined with monthly performance feedback; directed at the healthcare provider and delivered on screen Arm 2: standard CDS | |
| Outcomes | Dichotomous process measures: % patients receiving comprehensive care; % patients with pain treated; % patients with amoxicillin as first line therapy; % patients with high dose amoxicillin | |
| Notes | Data on other trial arms not extracted; Data on comparison arm 1 vs arm 2 is not reported in the paper | |
| ***Risk of bias*** |  |  |
| **Bias** | **Authors’ judgement** | **Support for judgement** |
| Random sequence generation | Unclear risk | Randomisation method not clear |
| Allocation concealment | Unclear risk | Not clear |
| Similar outcomes at baseline | High risk | Important differences |
| Similar characteristics at baseline | Low risk | No important differences |
| Incomplete outcome data | Low risk | Limited and balanced loss to follow up |
| Blinding of outcome assessment | Low risk | Objective outcomes |
| Adequate protection against contamination | Low risk | Allocation on practice level |
| Selective reporting | High risk | Of 13 outcomes reported at baseline, only 4 outcomes were reported at follow-up; Protocol was registered, but description of outcomes was vague |
| Other bias | Low risk |  |

Fortuna 2009

| Methods | Cluster RCT, unit of allocation: practice  Summary risk of bias: low risk | |
| --- | --- | --- |
| Participants | Patients in the included study arms: patients being prescribed hypnotic medications for insomnia; female: % not clear; mean age: not clear; number of patients not clear  Professionals: 195 internal medicine clinicians working at 9 sites received the interventions  Setting: outpatient, United States | |
| Interventions | Arm 1: CDS directed at healthcare provider and delivered on screen combined with educational sessions for healthcare providers  Arm 2: standard CDS | |
| Outcomes | Dichotomous process measures: % prescriptions for heavily marketed hypnotics (primary outcome), % prescriptions changed in response to CDS  Follow up period: 12 months | |
| Notes | Data on control arm not extracted, some data extracted from a graphic | |
| ***Risk of bias*** |  |  |
| **Bias** | **Authors’ judgement** | **Support for judgement** |
| Random sequence generation | Low risk | Random number generator |
| Allocation concealment | Low risk | Allocation by a statistician |
| Similar outcomes at baseline | Low risk | No important differences |
| Similar characteristics at baseline | Unclear risk | No information available on patient characteristics |
| Incomplete outcome data | Low risk | No loss to follow up |
| Blinding of outcome assessment | Low risk | Objective outcomes |
| Adequate protection against contamination | Low risk | Allocation by practice |
| Selective reporting | Low risk | Protocol registered and no selective reporting identified |
| Other bias |  |  |

Fricton 2011

| Methods | Cluster RCT, unit of allocation: practice  Summary risk of bias: unclear risk | |
| --- | --- | --- |
| Participants | Patients in the included study arms: patients with at least diabetes mellitus; congestive heart failure; chronic obstructive pulmonary disease or xerostomia; female: % not clear; mean age: not clear  Professionals: 64 dentists and hygienists received the interventions in the analysed arms  Setting: outpatient, United States | |
| Interventions | Arm 1: CDS directed at both patients (by e-mail or letter) and healthcare providers (delivered on screen)  Arm 2: CDS directed at healthcare providers | |
| Outcomes | Dichotomous process measures: % guideline website users, % guideline website visits for patients with the targeted medical condition  Continuous process measures: guideline website visits per provider  Follow up period: 12 months | |
| Notes |  | |
| ***Risk of bias*** |  |  |
| **Bias** | **Authors’ judgement** | **Support for judgement** |
| Random sequence generation | Low risk | Computer generated randomisation |
| Allocation concealment | Low risk | Randomisation by computer |
| Similar outcomes at baseline | Low risk | No important difference |
| Similar characteristics at baseline | Unclear risk | Not clear |
| Incomplete outcome data | Low risk | Limited loss to follow up |
| Blinding of outcome assessment | Low risk | Objective outcomes |
| Adequate protection against contamination | Unclear risk | Limited cross-over of professionals and patients |
| Selective reporting | Unclear risk | No protocol identified, additional process and patient outcomes are mentioned for publication in another report |
| Other bias | Low risk |  |

Heiman 2004

| Methods | Cluster RCT, unit of allocation: Clinics  Summary risk of bias: high risk | |
| --- | --- | --- |
| Participants | Patients in the included study arms: patients over 70 years or over 50 years with chronic illness; female: 65%; mean age: 72.6; n= 745 analysed  Professionals: five primary care clinics including 45 physicians that belong to 8 clusters  Setting: inpatient and outpatient, United States | |
| Interventions | Arm 1: CDS directed at the healthcare provider and provided on screen combined with patient directed information (letter)  Arm 2: CDS for healthcare provider | |
| Outcomes | Dichotomous process measures: % patients with a completed advance directive (primary outcome)  Follow up period: 6 months | |
| Notes | Data for the control group are not extracted | |
| ***Risk of bias*** |  |  |
| **Bias** | **Authors’ judgement** | **Support for judgement** |
| Random sequence generation | Low risk | Random number generator |
| Allocation concealment | Low risk | Allocation by a blinded programmer |
| Similar outcomes at baseline | Low risk | None of the patients had a completed advance directive |
| Similar characteristics at baseline | High risk | Important differences |
| Incomplete outcome data | Unclear risk | Not clear |
| Blinding of outcome assessment | Low risk | Objective outcomes |
| Adequate protection against contamination | Unclear risk | In families it is possible that participants were allocated to different arms |
| Selective reporting | Unclear risk | No protocol found |
| Other bias | Low risk |  |

Hendrix 2015

| Methods | Cluster RCT, unit of allocation: institution  Summary risk of bias: unclear risk | |
| --- | --- | --- |
| Participants | Patients in the included study arms: miscellaneous paediatric conditions; number of patients not reported; the CDS was triggered 2237 times  Professionals: Paediatricians, numbers not reported  Setting: outpatient, United States | |
| Interventions | Arm 1: colour-highlighting of CDS for half of the messages; directed at the healthcare provider and provided on paper  Arm2: highlighting for the other half of the CDS messages | |
| Outcomes | Dichotomous process measures: Response rate (any type of response) per CDS message  Follow up period: 3 months | |
| Notes | Data on response to high priority CDS are not considered, because this was active in both study arms and the comparison was made with the year before the study | |
| ***Risk of bias*** |  |  |
| **Bias** | **Authors’ judgement** | **Support for judgement** |
| Random sequence generation | Low risk | Randomisation by coin flip |
| Allocation concealment | Unclear risk | Not clear |
| Similar outcomes at baseline | Unclear risk | Not clear |
| Similar characteristics at baseline | Unclear risk | Not clear |
| Incomplete outcome data | Low risk | Computer-based outcome |
| Blinding of outcome assessment | Low risk | Objective computer-based outcome |
| Adequate protection against contamination | Low risk | Allocation by institution |
| Selective reporting | Low risk | Protocol registered and no selective reporting |
| Other bias | Low risk |  |

Kenealy 2005

| Methods | Cluster RCT, unit of allocation: provider  Summary risk of bias: unclear risk | |
| --- | --- | --- |
| Participants | Patients in the included study arms: patients eligible for diabetes screening; female: % not clear; mean age: not clear; n= 5628 analysed in all the arms  Professionals: 56 general practitioners received the included interventions  Setting: outpatient, New Zealand | |
| Interventions | Arm 1: CDS advice (on screen) directed at the healthcare provider combined with a patient completed screening form  Arm 2: CDS advice for healthcare provider | |
| Outcomes | Dichotomous process measures: % patients screened for diabetes  Follow up period: 2 months | |
| Notes | Data for other trial arms not used | |
| ***Risk of bias*** |  |  |
| **Bias** | **Authors’ judgement** | **Support for judgement** |
| Random sequence generation | Low risk | Random number generator |
| Allocation concealment | Low risk | Concealed allocation with sealed envelopes |
| Similar outcomes at baseline | Low risk | None of the patients had been screened in the last 3 years |
| Similar characteristics at baseline | Low risk | No important differences |
| Incomplete outcome data | Unclear risk | Data from two providers were excluded from the analysis, it is not clear how this affected the results |
| Blinding of outcome assessment | Low risk | Objective outcomes |
| Adequate protection against contamination | Unclear risk | Allocation of providers who may interact with providers from other arms that work within the same practice |
| Selective reporting | Unclear risk | No protocol identified |
| Other bias | Low risk |  |

Litzelman 1993

| Methods | Cluster RCT, unit of allocation: clinical practice session  Summary risk of bias: unclear risk | |
| --- | --- | --- |
| Participants | Patients in the included study arms: patients patients requiring fecal occult blood testing, mammography and cervical Papanicolaou testing.; female: 81%; mean age: 58; n= 5407 patients included in the analysis  Professionals: 176 primary care physicians  Setting: outpatient, United States | |
| Interventions | Arm 1: CDS, and reasons when overriding the advice were required; directed at the healthcare provider and delivered on paper  Arm 2: standard CDS | |
| Outcomes | Dichotomous process measures: % compliance with CDS Follow up period: 6 months | |
| Notes |  | |
| ***Risk of bias*** |  |  |
| **Bias** | **Authors’ judgement** | **Support for judgement** |
| Random sequence generation | Unclear risk | Not clear |
| Allocation concealment | Unclear risk | Not clear |
| Similar outcomes at baseline | Unclear risk | Not clear |
| Similar characteristics at baseline | Low risk | No important differences |
| Incomplete outcome data | Unclear risk | Not clear |
| Blinding of outcome assessment | Low risk | Objective outcomes |
| Adequate protection against contamination | Unclear risk | Not clear |
| Selective reporting | Unclear risk | No protocol identified |
| Other bias | Low risk |  |

Lobach 2013

| Methods | Cluster RCT, unit of allocation: family  Summary risk of bias: high risk | |
| --- | --- | --- |
| Participants | Patients in the included study arms: patients with miscellaneous conditions; female: 57%; mean age: not clear; n= 10098 analysed in the intervention arms (clinical and economic outcomes) and 169 analysed for quality of life outcomes  Professionals: care managers and clinic administrators received the interventions  Setting: outpatient, United States | |
| Interventions | Arm 1: CDS directed at patient whenever relevant delivered on paper  Arm 2: CDS directed at care manager on weekly basis delivered on screen  Arm 3: CDS directed at clinic administrator on quarterly basis delivered on paper | |
| Outcomes | Continuous process measures: missed appointments  Dichotomous patient measures: % rating excellent or very good on general health status scale  Continuous patient measures: emergency department encounters (primary outcome), outpatient encounters, hospitalisations, general health status (EQ-5D)  Economic measures: medical costs, emergency department encounters (primary outcome), outpatient encounters, care manager contacts, hospitalisations  Satisfaction measures: provider ratings, healthcare ratings  Follow up period: 9 months | |
| Notes |  | |
| ***Risk of bias*** |  |  |
| **Bias** | **Authors’ judgement** | **Support for judgement** |
| Random sequence generation | Low risk | Computer generated (additional information provided by the authors) |
| Allocation concealment | Low risk | Allocation by a computer (additional information provided by the authors) |
| Similar outcomes at baseline | Unclear risk | No baseline outcomes recorded (additional information provided by the authors) |
| Similar characteristics at baseline | Low risk | No important differences |
| Incomplete outcome data | Low risk | No missing data |
| Blinding of outcome assessment | Low risk | Primary outcome was objective |
| Adequate protection against contamination | High risk | Care managers and clinic administrators could also have contacts with patients from other arms |
| Selective reporting | High risk | Protocol registered, multiple planned outcomes were not reported, different primary outcome |
| Other bias | Low risk |  |

Loo 2011

| Methods | Cluster RCT, unit of allocation: provider  Summary risk of bias: high risk | |
| --- | --- | --- |
| Participants | Patients in the included study arms: elderly patients with due preventive procedures; female: 58.7%; mean age: 75; n= 2730 analysed in the included arms  Professionals: 34 primary care physicians received the interventions  Setting: outpatient, United States | |
| Interventions | Arm 1: CDS directed at the healthcare provider and provided on screen combined with a patient panel manager  Arm 2: standard CDS | |
| Outcomes | Dichotomous process measures: % patients with healthcare proxy designation, % patients with bone density screening, % patients with peumococcal vaccination, % patients with influenza vaccination  Follow up period: 12 months | |
| Notes | Type of funding not clear, Data from control group not extracted | |
| ***Risk of bias*** |  |  |
| **Bias** | **Authors’ judgement** | **Support for judgement** |
| Random sequence generation | Unclear risk | Randomisation method not clear |
| Allocation concealment | Unclear risk | Not clear |
| Similar outcomes at baseline | High risk | Important differences present |
| Similar characteristics at baseline | High risk | Important differences present |
| Incomplete outcome data | Unclear risk | Not clear |
| Blinding of outcome assessment | Low risk | Objective outcomes |
| Adequate protection against contamination | Unclear risk | Allocation of providers that worked within the same office |
| Selective reporting | Low risk | Protocol registered and no selective reporting identified |
| Other bias | Low risk |  |

Manns 2012

| Methods | Cluster RCT, unit of allocation: practice  Summary risk of bias: low risk | |
| --- | --- | --- |
| Participants | Patients in the included study arms: elderly chronic kidney disease patients with diabetes or proteinuria for the primary outcome and all chronic kidney disease patients > 18 years for other outcomes; female: 55,2% (primary outcome) and 63.8% (overall); mean age: 78.1 (primary outcome) and 72.2 (overall); n= 5444 analysed for the primary outcome and 22 092 overall  Professionals: 420 primary care physicians received the interventions and 354 primary care physicians at follow up  Setting: outpatient, Canada | |
| Interventions | Arm 1: a CDS laboratory message with specific diagnostic and management recommendations; directed at the healthcare provider (delivery method unclear)  Arm 2: a standard CDS laboratory message | |
| Outcomes | Dichotomous process measures: % with ACEi or ARB use among elderly chronic kidney disease patients with diabetes or proteinuria within 1 year of the ﬁrst CDS message (primary outcome); % patients with prescription of cholesterol-lowering medication; % patients with prescription of new antihypertensive medication from another class; % patients with consultation with a nephrologist; % patients with urinary albumin measurement; % patients with lipid measurement; % patients with A1C measurement  Dichotomous patient measures: % patients with composite clinical outcome (death, end-stage renal disease, doubling of serum creatinine, or hospitalization for myocardial infarction, heart failure, or stroke)  Follow up period: median 25 months | |
| Notes | Partial commercial funding | |
| ***Risk of bias*** |  |  |
| **Bias** | **Authors’ judgement** | **Support for judgement** |
| Random sequence generation | Low risk | computer-generated random numbers list |
| Allocation concealment | Low risk | Randomisation was concealed |
| Similar outcomes at baseline | Low risk | No important differences for primary outcome, not clear for secondary outcomes |
| Similar characteristics at baseline | Low risk | No important differences |
| Incomplete outcome data | Low risk | Minimal loss to follow-up |
| Blinding of outcome assessment | Low risk | Objective outcomes |
| Adequate protection against contamination | Low risk | Practices were randomised |
| Selective reporting | Low risk | Protocol was registered and no selective outcome reporting identified |
| Other bias | Low risk |  |

McAlister 2009

| Methods | Cluster RCT, unit of allocation: practice  Summary risk of bias: unclear risk | |
| --- | --- | --- |
| Participants | Patients in the included study arms persons with a coronary heart disease; female: 23.2%; mean age: 63,7; n= 354 analysed  Professionals: 252 primary care practices (including control group) received the interventions  Setting: outpatient, Canada | |
| Interventions | Arm 1: CDS delivered by fax and directed at physician and signed by an opinion leader  Arm 2: standard CDS | |
| Outcomes | Dichotomous process measures: % with initiation of statin or increase of dose (primary outcome), % taking a statin, % taking a nonstatin lipid-lowering drug, % taking any antiplatelet agent, % taking an ACE inhibitor or ARB, % taking a β-blocker, % taking triple therapy  Continuous process measures: standardised mean statin dose  Dichotomous patient measures: % with fasting LDL levels ≤ 2.0 mmol/L, % patients stopped smoking, % deaths  Economic measures: % with emergency department visits, % with hospitalisations  Follow up period: 6 months | |
| Notes | Data from a control group was not considered; Partial commercial funding | |
| ***Risk of bias*** |  |  |
| **Bias** | **Authors’ judgement** | **Support for judgement** |
| Random sequence generation | Low risk | computer-generated central randomisation system |
| Allocation concealment | Low risk | concealment of the randomisation list |
| Similar outcomes at baseline | Low risk | No important differences in statin use and dosage |
| Similar characteristics at baseline | Unclear risk | The amount of men is 10% lower in arm 1 |
| Incomplete outcome data | Low risk | Loss to follow-up is minimal and balanced across groups |
| Blinding of outcome assessment | Low risk | Investigators and outcome assessors were blinded |
| Adequate protection against contamination | Low risk | Allocation by practice |
| Selective reporting | Low risk | Protocol published and no selective reporting identified |
| Other bias | Low risk |  |

McDonald 1980

| Methods | Cluster RCT, unit of allocation: healthcare professional  Summary risk of bias: high risk | |
| --- | --- | --- |
| Participants | Patients in the included study arms: miscellaneous conditions; number of patients not reported; the CDS was triggered for 2533 conditions  Professionals: 9 internal medicine interns, 17 internal medicine residents, 5 nurse practitioners  Setting: outpatient, United States | |
| Interventions | Arm 1: CDS supplemented with bibliographic citations with the full text available in print upon request; the CDS is directed at the healthcare provider and provided on paper  Arm 2: standard CDS | |
| Outcomes | Dichotomous process measures: % compliance with the CDS  Follow up period: 15 weeks | |
| Notes | Control group data was not considered; results per specific type of CDS advice (order a test, record a finding, change a treatment, etc.) were not reported per arm | |
| ***Risk of bias*** |  |  |
| **Bias** | **Authors’ judgement** | **Support for judgement** |
| Random sequence generation | Unclear risk | Randomisation method is not clear |
| Allocation concealment | Unclear risk | Not clear |
| Similar outcomes at baseline | Unclear risk | Not clear |
| Similar characteristics at baseline | Unclear risk | Not clear |
| Incomplete outcome data | Unclear risk | Not clear |
| Blinding of outcome assessment | Low risk | Objective outcomes |
| Adequate protection against contamination | High risk | Cross-over design where every healthcare provider participated in every arm |
| Selective reporting | Unclear risk | No protocol identified |
| Other bias | Low risk |  |

McDonald 2005

| Methods | Cluster RCT, unit of allocation: provider  Summary risk of bias: high risk | |
| --- | --- | --- |
| Participants | Patients in the included study arms: patients with cancer pain; female: 67.2%; mean age: 63.3; n= 439 analysed in the included arms  Professionals: 218 nurses received the included interventions  Setting: outpatient, United States | |
| Interventions | Arm 1: CDS directed at the healthcare provider and delivered on screen combined with a multicomponent intervention  Arm 2: standard CDS | |
| Outcomes | Dichotomous process measures: % patients receiving recommended nurse assessments, % patients receiving recommended nurse instruction, % use of alternative treatments  Dichotomous patient measures: % patients with hospitalisation, % patients with emergency department visits, % patients indicating high quality of life, % patients with severe pain, % patients with severe insomnia, % patients with severe constipation  Continuous patient measures: level of pain at its worst, level of pain on average, score on pain interference scale  Economic measures: home care related costs, overall costs, home care related costs of a 10% reduction in pain and in probability of hospitalisation  Follow up period: 1,5 months | |
| Notes | Data for control group not extracted; data for patient related barriers to pain management not extracted; data for patient perceptions on inadequate medication management not extracted | |
| ***Risk of bias*** |  |  |
| **Bias** | **Authors’ judgement** | **Support for judgement** |
| Random sequence generation | Unclear risk | Randomisation procedure not described |
| Allocation concealment | Unclear risk | Not clear |
| Similar outcomes at baseline | Unclear risk | Not clear |
| Similar characteristics at baseline | High risk | Important differences present |
| Incomplete outcome data | Unclear risk | Important loss to follow up, but reasons are balanced |
| Blinding of outcome assessment | Low risk | Objective outcomes and blinded assessment |
| Adequate protection against contamination | Unclear risk | Not clear if interactions between nurses could have created contamination |
| Selective reporting | Unclear risk | No protocol identified |
| Other bias | Low risk |  |

McPhee 1989

| Methods | Cluster RCT , unit of allocation: provider  Summary risk of bias: unclear risk | |
| --- | --- | --- |
| Participants | Patients in the included study arms: patients due for cancer screening; female: 100%; mean age: not clear; n= 1936 analysed overall (including two other trial arms)  Professionals: 21 residents received the included interventions  Setting: outpatient, United States | |
| Interventions | Arm 1: CDS directed at the healthcare provider combined with CDS directed at the patient and delivered on paper  Arm 2: CDS directed at the healthcare provider and delivered on paper | |
| Outcomes | Dichotomous process measures: % patients with completed mammography, % patients with completed breast examination  Follow up period: 9 months | |
| Notes | Data from other trial arms not used | |
| ***Risk of bias*** |  |  |
| **Bias** | **Authors’ judgement** | **Support for judgement** |
| Random sequence generation | Unclear risk | Randomisation procedure is not clear |
| Allocation concealment | Unclear risk | Not clear |
| Similar outcomes at baseline | Unclear risk | No data are available for the two specific arms |
| Similar characteristics at baseline | Unclear risk | Not clear |
| Incomplete outcome data | Low risk | The authors analysed a random sample of eligible patients |
| Blinding of outcome assessment | Low risk | Objective outcomes |
| Adequate protection against contamination | Low risk |  |
| Selective reporting | Unclear risk | No protocol identified |
| Other bias | Low risk |  |

Meeker 2016

| Methods | Cluster RCT, unit of allocation: practice  Summary risk of bias: high risk | |
| --- | --- | --- |
| Participants | Patients in the included study arms: patients with acute respiratory infections; female: 67%; mean age: 47; n= 4519 qualifying patient visits in arm 1 and 2and n=4623 qualifying patient visits in arm 2 and 3  Professionals: 47 primary care practices, including 248 physicians received the intervention  Setting: outpatient, United States | |
| Interventions | Arm 1: CDS (directed at the healthcare professional and delivered on screen) supplemented with documentation of override reasons and monthly performance feedback  Arm 2: CDS supplemented with documentation of override reasons  Arm 3: standard CDS | |
| Outcomes | Dichotomous process measures: % of inappropriate antibiotic prescriptions  Follow up period: 18 months | |
| Notes | Data for other comparisons not extracted | |
| ***Risk of bias*** |  |  |
| **Bias** | **Authors’ judgement** | **Support for judgement** |
| Random sequence generation | Low risk | Computerised randomisation programme |
| Allocation concealment | Low risk | Concealed allocation |
| Similar outcomes at baseline | High risk | Important baseline differences |
| Similar characteristics at baseline | High risk | Important baseline differences |
| Incomplete outcome data | Low risk | Limited loss to follow-up that is balanced |
| Blinding of outcome assessment | Low risk | Data extracted from the electronic record |
| Adequate protection against contamination | Low risk | Allocation by practice |
| Selective reporting | Low risk | Protocol registered and no selective reporting identified |
| Other bias | Low risk |  |

Murray 2004

| Methods | Cluster RCT, unit of allocation: practice sessions, pharmacists and patients  Summary risk of bias: high risk (patient reported outcomes), unclear risk (other outcomes) | |
| --- | --- | --- |
| Participants | Patients in the included study arms: patients with hypertension; female: 79.3%; mean age: 54.7; n= 356 (for patient reported outcomes), 541 (for other outcomes) analysed  Professionals: general practitioners and pharmacists  Setting: outpatient, United States | |
| Interventions | Arm 1: CDS directed at physician (on screen) and pharmacist (on paper)  Arm 2: CDS directed at pharmacist  Arm 3: CDS directed at physician | |
| Outcomes | Dichotomous process measures: % compliance with CDS advice  Continuous patient measures: generic health-related quality of life (primary outcome), number of emergency department visits, number of hospitalisations, systolic blood pressure, diastolic blood pressure  Economic measures: total health care charges  Follow up period: 12 months | |
| Notes | Data from control group not used | |
| ***Risk of bias*** |  |  |
| **Bias** | **Authors’ judgement** | **Support for judgement** |
| Random sequence generation | Unclear risk | Randomisation method not clear |
| Allocation concealment | Unclear risk | Not clear |
| Similar outcomes at baseline | Unclear risk | Not clear |
| Similar characteristics at baseline | Low risk |  |
| Incomplete outcome data | High risk (patient reported outcomes)  Low risk (other outcomes) | Important loss to follow up for the patient reported outcomes with some imbalance |
| Blinding of outcome assessment | Low risk | Patient reported outcomes were assessed by blinded outcome assessors, the other outcomes were objective |
| Adequate protection against contamination | Low risk |  |
| Selective reporting | Unclear risk | No protocol identified, outcomes on patient satisfaction are not reported |
| Other bias | Low risk |  |

Nendaz 2010

| Methods | Controlled before and after study, unit of allocation: Institution and patient  Summary risk of bias: high risk | |
| --- | --- | --- |
| Participants | Patients in the included study arms: acutely ill patients; female: 48%; mean age: 68; n= 290 analysed before and n=204 analysed after  Professionals: clinicians from 9 medical services in 8 hospitals  Setting: Inpatient, Switzerland | |
| Interventions | Arm 1: CDS provided automatically, directed at healthcare providers and delivered on screen in the electronic chart  Arm 2: CDS available on demand through a personal digital assistant and requiring input of patient data | |
| Outcomes | Dichotomous process measures: % patients with adequate prescription decisions  Follow up period: data collection before the intervention and after a 4 months intervention period for those patients admitted on one specific day | |
| Notes | Data from one trial arm not used, extra information provided by the authors | |
| ***Risk of bias*** |  |  |
| **Bias** | **Authors’ judgement** | **Support for judgement** |
| Random sequence generation | High risk | Non randomised study |
| Allocation concealment | High risk |  |
| Similar outcomes at baseline | High risk | Important differences present |
| Similar characteristics at baseline | Unclear risk | Not reported per arm |
| Incomplete outcome data | Unclear risk |  |
| Blinding of outcome assessment | Low risk | Objective outcomes |
| Adequate protection against contamination | Low risk | Allocation by institution |
| Selective reporting | Unclear risk | No protocol identified |
| Other bias | Low risk |  |

Ornstein 1991

| Methods | Cluster RCT, unit of allocation: practice group  Summary risk of bias: high risk | |
| --- | --- | --- |
| Participants | Patients in the included study arms patients with due preventive procedures: x; female: 60.7%; mean age: 40.2; n= 5 821 randomised for the included arms  Professionals: 39 family physicians received the included interventions.  Setting: outpatient, United States | |
| Interventions | Arm 1: CDS directed at healthcare provider (delivered on paper) and at patient (delivered on paper)  Arm 2: CDS directed at patient  Arm 3: CDS directed at healthcare provider | |
| Outcomes | Dichotomous process measures: % patients with completed mammogram, % patients with completed pap smear, % patients with completed fecal occult blood test, % patients with completed cholesterol test, % patients with completed tetanus vaccinations  Follow up period: 12 months | |
| Notes | Data on control group not used | |
| ***Risk of bias*** |  |  |
| **Bias** | **Authors’ judgement** | **Support for judgement** |
| Random sequence generation | Unclear risk | Randomisation method not clear |
| Allocation concealment | Unclear risk | Not clear |
| Similar outcomes at baseline | High risk | Important differences for three outcomes |
| Similar characteristics at baseline | High risk | Important differences |
| Incomplete outcome data | Unclear risk | It is unclear if the loss to follow up is balanced |
| Blinding of outcome assessment | Low risk | Objective outcomes |
| Adequate protection against contamination | Low risk | Allocation of practice groups |
| Selective reporting | Unclear risk | No protocol identified |
| Other bias | Low risk |  |

Persell 2008

| Methods | Cluster RCT, unit of allocation: healthcare provider  Summary risk of bias: high risk | |
| --- | --- | --- |
| Participants | Patients in the included study arms: patients with diabetes; female: 62.8%; mean age: 57.9; n= 242 analysed  Professionals: 19 internal medicine physicians from one practice received the interventions  Setting: outpatient, United States | |
| Interventions | Arm 1: CDS directed at healthcare providers (on screen in EMR and by email) and directed at patients (letter and telephone)  Arm 2: CDS directed at healthcare providers (on screen in EMR only) | |
| Outcomes | Dichotomous process measures: % patients with regular aspirin use  Follow up period: 6 months | |
| Notes |  | |
| ***Risk of bias*** |  |  |
| **Bias** | **Authors’ judgement** | **Support for judgement** |
| Random sequence generation | Low risk | Random number generator |
| Allocation concealment | Low risk | Allocation by a blinded programmer |
| Similar outcomes at baseline | Low risk | No important differences |
| Similar characteristics at baseline | High risk | Important differences for gender and race |
| Incomplete outcome data | Unclear risk | Loss to follow up for 28% of patients which was balanced over the groups |
| Blinding of outcome assessment | Low risk | The research assistant that conducted the patient interviews was blinded (additional information provided by the authors) |
| Adequate protection against contamination | Unclear risk | Providers from both arms worked together in one practice |
| Selective reporting | Low risk | Protocol registered |
| Other bias | Low risk |  |

Rimer 1999

| Methods | RCT, unit of allocation: patient  Summary risk of bias: high risk | |
| --- | --- | --- |
| Participants | Patients in the included study arms: patients due for cancer screening; female: 100%; mean age: 51; n= 627 analysed  Professionals: clinicians working in a community clinic  Setting: outpatient, United States | |
| Interventions | Arm 1: CDS directed at the healthcare provider and delivered on paper combined with CDS directed at the patient and tailored telephone counselling  Arm 2: CDS directed at the healthcare provider | |
| Outcomes | Dichotomous process measures: % patients compliant with screening  Follow up period: 16 months | |
| Notes | Data for another trial arm not extracted | |
| ***Risk of bias*** |  |  |
| **Bias** | **Authors’ judgement** | **Support for judgement** |
| Random sequence generation | Unclear risk | Randomisation method not clear |
| Allocation concealment | Unclear risk | Not clear |
| Similar outcomes at baseline | Unclear risk | Not clear |
| Similar characteristics at baseline | Unclear risk | No data available per group |
| Incomplete outcome data | High risk | Large loss to follow up (>25%) |
| Blinding of outcome assessment | Low risk | Objective outcomes |
| Adequate protection against contamination | High risk | Randomisation of patients who could interact |
| Selective reporting | Unclear risk | No protocol identified |
| Other bias | High risk | Patients were asked if they had had screening tests, this was not extracted from EMR. Patients from the patient directed groups might recall this better than patients from the other group. |

Robbins 2012

| Methods | RCT, unit of allocation: patient  Summary risk of bias: high risk | |
| --- | --- | --- |
| Participants | Patients in the included study arms: patients with HIV; female: 21.6%; age: 74.8% > 40 year  Professionals: 33 healthcare providers received the intervention (11 attending physicians, 20 infectious disease fellows, 2 nurse practitioners)  Setting: outpatient, United States | |
| Interventions | Arm 1: interactive CDS with semi-automated actions and delivered on screen through different channels, directed at the healthcare provider  Arm 2: static CDS without semi-automated actions and delivered through one channel | |
| Outcomes | Continuous process measures: event rate of patients without follow-up appointments over 6 months, time to next scheduled appointment after CDS  Dichotomous patient measures: event rate of grade 3 or 4 toxicity per 100 patient years, event rate of confirmed virological failure  Continuous patient measures: mean CD4 cell count increase (primary outcome)  Follow up period: 12 months | |
| Notes | We did not extract data on user attitudes since satisfaction was not explicitly measured | |
| ***Risk of bias*** |  |  |
| **Bias** | **Authors’ judgement** | **Support for judgement** |
| Random sequence generation | Low risk | Randomisation by computer |
| Allocation concealment | Low risk | Automated randomisation |
| Similar outcomes at baseline | Low risk | No important differences for primary outcome |
| Similar characteristics at baseline | Low risk | No important differences |
| Incomplete outcome data | High risk | Imbalance in loss to follow up |
| Blinding of outcome assessment | Low risk | Objective outcomes |
| Adequate protection against contamination | High risk | Healthcare providers received CDS from both arms |
| Selective reporting | High risk | Protocol was registered, one planned secondary outcome (time to repeat laboratory testing) was not reported |
| Other bias | Low risk |  |

Rosenberg 2008

| Methods | Controlled before and after study, unit of allocation: institution  Summary risk of bias: high risk | |
| --- | --- | --- |
| Participants | Patients in the included study arms: patients with miscellaneous conditions and needs related to vaccination, screening, diagnosis medication; female: 51,8%; mean age: 33.5; n= 77304 CDS alerts/per 1000 members analysed at follow up with a total membership of 1003442  Professionals: physicians working for 32 large employers  Setting: outpatient, United States | |
| Interventions | Arm 1: CDS directed at healthcare providers (delivered by telephone, fax or mail) and patients (delivered by mail)  Arm 2: CDS directed at healthcare providers | |
| Outcomes | Dichotomous process measures: % compliance with the CDS advice  Follow up period: 12 months | |
| Notes |  | |
| ***Risk of bias*** |  |  |
| **Bias** | **Authors’ judgement** | **Support for judgement** |
| Random sequence generation | High risk | No randomisation |
| Allocation concealment | High risk | No concealment of allocation |
| Similar outcomes at baseline | High risk | Important baseline differences per alert type |
| Similar characteristics at baseline | Unclear risk | The amount of baseline data on patient characteristics is limited |
| Incomplete outcome data | Low risk | Outcomes were based on electronically available data |
| Blinding of outcome assessment | Low risk | Objective outcomes |
| Adequate protection against contamination | Low risk | Allocation by institution |
| Selective reporting | Unclear risk | No protocol identified |
| Other bias | Low risk |  |

Rosenbloom 2005

| Methods | Cluster RCT, unit of allocation: practice  Summary risk of bias: high risk | |
| --- | --- | --- |
| Participants | Patients in the included study arms: patients with miscellaneous conditions; no data on patients reported; n= 418739 CDS opportunities analysed  Professionals: 147 physicians received the interventions  Setting: inpatient, United States | |
| Interventions | Arm 1: CDS initiated automatically through direct display for healthcare providers  Arm 2: on screen CDS that is available on demand for healthcare providers | |
| Outcomes | Dichotomous process measures: % responses to CDS opportunities  Economic measures: Expenditure per order-entry session  Follow up period: 11.5 months | |
| Notes |  | |
| ***Risk of bias*** |  |  |
| **Bias** | **Authors’ judgement** | **Support for judgement** |
| Random sequence generation | Unclear risk | Not clear |
| Allocation concealment | Low risk | Allocation by a study statistician |
| Similar outcomes at baseline | Unclear risk | Not clear |
| Similar characteristics at baseline | Unclear risk | Not clear |
| Incomplete outcome data | Low risk | Automatic data collection |
| Blinding of outcome assessment | Low risk | Objective outcomes |
| Adequate protection against contamination | High risk | Cross-over of some participants and participants were re-randomised in year 2 |
| Selective reporting | Unclear risk | No protocol identified |
| Other bias | Low risk |  |

Rosser 1991

| Methods | Cluster RCT, unit of allocation: patient or family  Summary risk of bias: high risk | |
| --- | --- | --- |
| Participants | Patients in the included study arms: patients 15 years and older that have due preventive procedures; female: 57%; mean age: not reported; n= 4480 analysed  Professionals: 4 teams (including a staff physician, a nurse and three to five residents) received the interventions (including the randomised control group)  Setting: outpatient, Canada | |
| Interventions | Arm 1 (two intervention arms combined): CDS information on due preventive procedures delivered to patients by letter or telephone  Arm 2: CDS information on due preventive procedures; delivered on paper to healthcare professionals before any visit | |
| Outcomes | Dichotomous process measures: % patients with due preventive procedures performed  Economic measures: cost per preventive procedure gained  Follow up period: 12 months | |
| Notes | Economic measures data taken from related papers; Control group data not considered; Data combined for patient reminder groups | |
| ***Risk of bias*** |  |  |
| **Bias** | **Authors’ judgement** | **Support for judgement** |
| Random sequence generation | Low risk | Use of a randomisation computer programme |
| Allocation concealment | Unclear risk | Not clear |
| Similar outcomes at baseline | Unclear risk | Not clear |
| Similar characteristics at baseline | Low risk | No important differences |
| Incomplete outcome data | High risk | Only half of the patients assigned to the physician reminder group, visited the practice during the trial |
| Blinding of outcome assessment | Low risk | Objective outcomes |
| Adequate protection against contamination | High risk | Physicians treated patients from multiple arms |
| Selective reporting | Unclear risk | No protocol identified |
| Other bias | Low risk |  |

Roumie 2006

| Methods | Cluster RCT, unit of allocation: provider  Summary risk of bias: high risk | |
| --- | --- | --- |
| Participants | Patients in the included study arms: patients with uncontrolled hypertension; female: 3.8%; mean age: 65; n= 720 analysed  Professionals: 128 clinicians received the included interventions  Setting: outpatient, United States | |
| Interventions | Arm 1: CDS directed at the healthcare provider and delivered on screen combined with patient directed information  Arm 2: CDS directed at the healthcare provider | |
| Outcomes | Dichotomous process measures: % patients with any changes in antihypertensive drugs  Dichotomous patient measures: % patients with systolic blood pressure ≤140 (primary outcome), % patients with diastolic blood pressure < 90, % patients with hospitalisations, % patients with deaths  Continuous patient measures: systolic blood pressure, patient medication adherence  Follow up period: 6 months | |
| Notes | Data from control group not used | |
| ***Risk of bias*** |  |  |
| **Bias** | **Authors’ judgement** | **Support for judgement** |
| Random sequence generation | Low risk | Computer generated randomisation sequence |
| Allocation concealment | Low risk | Assignment by a researcher who was blinded |
| Similar outcomes at baseline | Low risk | No important differences |
| Similar characteristics at baseline | Low risk |  |
| Incomplete outcome data | High risk | Important loss to follow up which is not balanced across the arms |
| Blinding of outcome assessment | Low risk | Objective outcomes |
| Adequate protection against contamination | Unclear risk | Some contamination occurred when patients were reassigned to another provider, but the magnitude is not clear |
| Selective reporting | Low risk | Protocol registered and no selective reporting identified |
| Other bias | Low risk |  |

Scheepers-Hoeks 2013

| Methods | RCT, unit of allocation: patient  Summary risk of bias: high risk | |
| --- | --- | --- |
| Participants | Patients in the included study arms: patients requiring intensive care unit treatment; female: 45.6%; mean age: 65.8; n= 384 patients with alerts in the analysis  Professionals: no data reported  Setting: inpatient, the Netherlands | |
| Interventions | Arm 1: on screen CDS for individual patients that is delivered automatically to the physician  Arm 2: on screen CDS for individual patients that is available on demand and directed at the physician  Arm 3: on screen list of CDS per day that is directed at the pharmacist  Arm 4: on screen list of CDS per day that is directed at the physician | |
| Outcomes | Dichotomous process measures: % of CDS followed within 24h  Other: Score on provider satisfaction  Follow up period: not clear | |
| Notes | Source of funding is not clear | |
| ***Risk of bias*** |  |  |
| **Bias** | **Authors’ judgement** | **Support for judgement** |
| Random sequence generation | Low risk | Computerised randomisation system |
| Allocation concealment | Low risk | Randomisation by a computer |
| Similar outcomes at baseline | Unclear risk | Not clear |
| Similar characteristics at baseline | High risk | Important differences on amount of generated CDS per group |
| Incomplete outcome data | Low risk | No loss to follow up |
| Blinding of outcome assessment | Unclear risk | Not clear |
| Adequate protection against contamination | High risk | Physicians treated patients from different arms |
| Selective reporting | Unclear risk | No protocol identified |
| Other bias | Low risk |  |

Schwarz 2012

| Methods | Cluster RCT, unit of allocation: healthcare professional  Summary risk of bias: unclear risk | |
| --- | --- | --- |
| Participants | Patients in the included study arms: women aged 18–50 years with no evidence of sterilization, menopause or infertility; female: 100%; mean age: 34; n= 35111 patient encounters analysed  Professionals: 42 general practitioners received the interventions  Setting: outpatient, United States | |
| Interventions | Arm 1: CDS message tailored to women’s likelihood of pregnancy and combined with an order set; directed at the healthcare provider and delivered on screen  Arm 2: Standard CDS message on potentially teratogenic medications for women who may become pregnant | |
| Outcomes | Dichotomous process measures: % encounters with documented provision of family planning services when potential teratogens prescribed (primary outcome); % Encounters with a teratogenic prescription  Other: Score on provider satisfaction  Follow up period: 9 month intervention period with an additional 9 month follow up | |
| Notes |  | |
| ***Risk of bias*** |  |  |
| **Bias** | **Authors’ judgement** | **Support for judgement** |
| Random sequence generation | Unclear risk |  |
| Allocation concealment | Unclear risk |  |
| Similar outcomes at baseline | Low risk | No important differences |
| Similar characteristics at baseline | Low risk | No important differences |
| Incomplete outcome data | Low risk | Minimal loss to follow-up |
| Blinding of outcome assessment | Low risk | Objective outcomes |
| Adequate protection against contamination | Unclear risk | Impact of interactions between providers is not clear |
| Selective reporting | Low risk | Protocol registered and no selective reporting identified |
| Other bias | Low risk | Data on the provision of family planning services was obtained through a computerised search string of patient records, but this data was not documented in a standardised way, which might affect the estimates. |

Sequist 2009

| Methods | Cluster RCT, unit of allocation: providers and patients  Summary risk of bias: unclear risk | |
| --- | --- | --- |
| Participants | Patients in the included study arms: patients eligible for colon cancer screening; female: 60%; mean age: not clear; n= 10912 analysed in the included arms  Professionals: 110 primary care physicians received the interventions  Setting: outpatient, United States | |
| Interventions | Arm 1: CDS directed at healthcare providers (on screen) and patients (on paper)  Arm 2: CDS directed at healthcare providers | |
| Outcomes | Dichotomous process measures: % patients screened (primary outcome)  Dichotomous patient measures: % patients with pathological findings  Follow up period: 15 months | |
| Notes | Data for control group and one trial arm not used | |
| ***Risk of bias*** |  |  |
| **Bias** | **Authors’ judgement** | **Support for judgement** |
| Random sequence generation | Unclear risk | Randomisation method not clear |
| Allocation concealment | Unclear risk | Not clear for randomisation of patients |
| Similar outcomes at baseline | Low risk | Screening rates were equal by design |
| Similar characteristics at baseline | Low risk | No important differences |
| Incomplete outcome data | Low risk | No loss to follow up |
| Blinding of outcome assessment | Low risk | Objective outcomes |
| Adequate protection against contamination | Unclear risk | Physicians had patients from different arms. Physicians from different arms could work in the same centre with colleagues from other arms |
| Selective reporting | Low risk | Protocol registered and no selective reporting identified |
| Other bias | Low risk |  |

Simon 2000

| Methods | RCT, unit of allocation: patient  Summary risk of bias: high risk | |
| --- | --- | --- |
| Participants | Patients in the included study arms: patients with a new prescription for antidepressants to treat depression; female: 72%; mean age: 46,4; n= 395 analysed  Professionals: 5 primary care practices with staff of 8-10 doctors, 3-5 registered nurses and 8-10 licensed practical nurses or medical assistants that received the interventions (including the control group)  Setting: outpatient, United States | |
| Interventions | Arm 1: patient specific treatment data and recommendations (provided at two points in time, directed at the healthcare professionals, delivery method not clear) supplemented with care managers that obtained extra data from the patient that was used for the CDS  Arm 2: standard CDS | |
| Outcomes | Dichotomous process measures: % taking antidepressants in low dose in 6 months, % taking antidepressants in moderate dose in 6 months  Dichotomous patient measures: % with a 50% decrease in depression score, % with a major depression at 6 months  Continuous patient measures: depression scale scores, number of outpatient visits after antidepressant prescription  Economic measures: depression treatment costs (primary outcome), health services costs, Time in treatment costs, number of outpatient visits after antidepressant prescription  Follow up period: 6 months | |
| Notes | Control group data was not considered; Some data extracted from a graphic; depression treatment costs was considered as the primary outcome since this variable was used for the sample size calculation | |
| ***Risk of bias*** |  |  |
| **Bias** | **Authors’ judgement** | **Support for judgement** |
| Random sequence generation | Low risk | Use of computer generated random numbers |
| Allocation concealment | Unclear risk | Not clear |
| Similar outcomes at baseline | Unclear risk | Not clear |
| Similar characteristics at baseline | Low risk | No important differences |
| Incomplete outcome data | Unclear risk | Limited loss to follow up, no important differences between arms |
| Blinding of outcome assessment | Low risk | Blinded outcome assessors |
| Adequate protection against contamination | High risk | Physicians treated patients from both arms |
| Selective reporting | Unclear risk | No protocol identified |
| Other bias | Low risk | No other risk of bias identified |

Simon 2001

| Methods | RCT, unit of allocation: patient  Summary risk of bias: unclear risk | |
| --- | --- | --- |
| Participants | Patients in the included study arms: patients due for breast cancer screening; female: 100%; mean age: not clear; n= 1717 analysed  Professionals: general practitioners from 2 clinics  Setting: outpatient, United States | |
| Interventions | Arm 1: CDS for healthcare provider delivered on paper combined with CDS for patient delivered on paper (referral to physician)  Arm 2: CDS for healthcare provider delivered on paper combined with CDS for patient delivered on paper (referral to direct care)  Arm 3: CDS for healthcare provider | |
| Outcomes | Dichotomous process measures: % patients with mammogram performed  Follow up period: 12 months | |
| Notes | Data are reported and extracted separately for two sites | |
| ***Risk of bias*** |  |  |
| **Bias** | **Authors’ judgement** | **Support for judgement** |
| Random sequence generation | Unclear risk | Randomisation method is not clear |
| Allocation concealment | Unclear risk | Not clear |
| Similar outcomes at baseline | Unclear risk | Not clear |
| Similar characteristics at baseline | Unclear risk | No data available per arm |
| Incomplete outcome data | Unclear risk | Not clear |
| Blinding of outcome assessment | Low risk | Objective outcomes |
| Adequate protection against contamination | Unclear risk | Allocation at patient level and interaction between patients is possible |
| Selective reporting | Unclear risk | No protocol identified |
| Other bias | Low risk |  |

Simon 2006

| Methods | Cluster RCT, unit of allocation: practice  Summary risk of bias: low risk | |
| --- | --- | --- |
| Participants | Patients in the included study arms: elderly patients with medication prescriptions; female: 62.2-65.8%; mean age: 73.6-74.3; number of patients analysed not clear  Professionals: 239 healthcare providers (physicians, nurses practitioners, physician assistants) from 15 practices received the interventions  Setting: outpatient, United States | |
| Interventions | Arm 1: CDS directed at the healthcare provider and delivered on screen combined with academic detailing in group  Arm 2: standard CDS | |
| Outcomes | Continuous process measures: Quarterly rates of use of target medications to avoid per 10000 patients  Follow up period: 15 months | |
| Notes |  | |
| ***Risk of bias*** |  |  |
| **Bias** | **Authors’ judgement** | **Support for judgement** |
| Random sequence generation | Unclear risk | Randomisation method not specified |
| Allocation concealment | Low risk | Allocation by practice |
| Similar outcomes at baseline | Low risk | No important differences |
| Similar characteristics at baseline | Low risk | No important differences |
| Incomplete outcome data | Low risk | Data extracted from automated claims system |
| Blinding of outcome assessment | Low risk | Objective outcomes and blinded assessment |
| Adequate protection against contamination | Low risk | Allocation by practice |
| Selective reporting | Unclear risk | No protocol identified |
| Other bias | Low risk |  |

Skinner 2015

| Methods | Cluster RCT , unit of allocation: provider  Summary risk of bias: high risk | |
| --- | --- | --- |
| Participants | Patients in the included study arms: patients potentially eligible for colon cancer screening; female: 64.7%; mean age: 58.3; n= 651 analysed  Professionals: Physicians from 1 family practice and 2 general internal medicine received the interventions  Setting: outpatient, United States | |
| Interventions | Arm 1: CDS message tailored to risk factors and including patient’s concerns; directed at the healthcare provider and directed at the patient and provided on paper  Arm 2: standard CDS message; directed at the healthcare provider and delivered on-screen and directed at the patient and provided on paper | |
| Outcomes | Dichotomous process measures: % patients with risk-appropriate colorectal cancer testing (primary outcome), % with any colorectal cancer testing  Follow up period: 12 months | |
| Notes | Data for the no-contact control group not extracted given that this group was not part of the randomisation process | |
| ***Risk of bias*** |  |  |
| **Bias** | **Authors’ judgement** | **Support for judgement** |
| Random sequence generation | Unclear risk |  |
| Allocation concealment | Low risk | Randomisation by professional at the start of the study |
| Similar outcomes at baseline | Low risk | Eligible patients had no relevant screening tests in the previous 10 years |
| Similar characteristics at baseline | High risk | Important differences present |
| Incomplete outcome data | Low risk | Large loss to follow-up but mainly due to exclusion of patients that were not eligible for screening |
| Blinding of outcome assessment | Low risk | Objective outcomes |
| Adequate protection against contamination | Unclear risk | The healthcare providers collaborated in the same practices, it is unclear if this created contamination |
| Selective reporting | Low risk | Protocol registered and no selective reporting identified |
| Other bias | Low risk |  |

Strom 2010

| Methods | Cluster RCT, unit of allocation: provider  Summary risk of bias: high risk | |
| --- | --- | --- |
| Participants | Patients in the included study arms: patients with concurrent orders for warfarin and NSAIDs; female: not clear; mean age: not clear; n= 528 patients included in the analysis  Professionals: 1963 resident physicians and nurse practitioners received the intervention  Setting: inpatient, United States | |
| Interventions | Arm 1: CDS and a response to the advice is required; directed at the healthcare provider and delivered on screen Arm 2: standard CDS | |
| Outcomes | Dichotomous process measures: % desired ordering responses after CDS  Follow up period: 15 months | |
| Notes |  | |
| ***Risk of bias*** |  |  |
| **Bias** | **Authors’ judgement** | **Support for judgement** |
| Random sequence generation | Unclear risk | Not clear |
| Allocation concealment | Unclear risk | Not clear |
| Similar outcomes at baseline | Unclear risk | Not clear |
| Similar characteristics at baseline | Unclear risk | Not clear |
| Incomplete outcome data | Low risk | Data automatically stored |
| Blinding of outcome assessment | Low risk | Objective outcomes |
| Adequate protection against contamination | High risk | Medication orders were discussed in teams, including participants from different trial arms |
| Selective reporting | Low risk | Protocol registered and no selective reporting identified |
| Other bias | Low risk |  |

Subramanian 2004

| Methods | Cluster RCT, unit of allocation: provider  Summary risk of bias: unclear risk | |
| --- | --- | --- |
| Participants | Patients in the included study arms: patients with heart failure and left ventricular systolic dysfunction; female: 2%; mean age: 69; n= 720 analysed  Professionals: 91 primary care physicians from 4 practices received the interventions  Setting: outpatient, United States | |
| Interventions | Arm 1: CDS directed at the healthcare provider and delivered on paper combined with a strategy to collect additional patient data  Arm 2: standard CDS | |
| Outcomes | Dichotomous process measures: % CDS suggestions adhered to  Dichotomous patient measures: % patients with improvement in NYHA class  Continuous patient measures: Generic health-related quality of life, all cause hospitalisations, outpatient visits  Economic measures: all cause hospitalisations, outpatient visits  Satisfaction measures: Patient satisfaction with physician, patient satisfaction with most recent visit  Follow up period: 12 months | |
| Notes |  | |
| ***Risk of bias*** |  |  |
| **Bias** | **Authors’ judgement** | **Support for judgement** |
| Random sequence generation | Low risk | Randomisation by coin flip |
| Allocation concealment | Low risk | Allocation of professionals at the start of study |
| Similar outcomes at baseline | Unclear risk | Only data available for one outcome |
| Similar characteristics at baseline | Low risk | No important differences |
| Incomplete outcome data | Unclear risk | Not clear |
| Blinding of outcome assessment | Low risk | Outcomes were partially objective, patient reported or collected by blinded outcome assessors |
| Adequate protection against contamination | Unclear risk | It is not known how much cross over there was from patients |
| Selective reporting | Unclear risk | No protocol identified |
| Other bias | Low risk |  |

Tamblyn 2008

| Methods | Cluster RCT, unit of allocation: practice  Summary risk of bias: high risk | |
| --- | --- | --- |
| Participants | Patients in the included study arms: patients with miscellaneous conditions; female: 61.2%; mean age: 67.1; n= 3449 patients included in the analysis  Professionals: 28 general practitioners or family physicians received the intervention  Setting: outpatient, Canada | |
| Interventions | Arm 1: CDS initiated automatically; delivered on screen and directed at the healthcare provider  Arm 2: CDS available on demand | |
| Outcomes | Dichotomous process measures: % of prescribing problems seen, % of prescribing problems revised  Continuous process measures: prevalence of prescription problems (primary outcome)  Follow up period: 6 months | |
| Notes |  | |
| ***Risk of bias*** |  |  |
| **Bias** | **Authors’ judgement** | **Support for judgement** |
| Random sequence generation | Unclear risk | Not clear |
| Allocation concealment | Unclear risk | Not clear |
| Similar outcomes at baseline | High risk | Important differences |
| Similar characteristics at baseline | High risk | Mean number of prescription problems/patient is higher in arm 2 |
| Incomplete outcome data | Unclear risk | Not clear |
| Blinding of outcome assessment | Low risk | Objective outcomes |
| Adequate protection against contamination | Low risk | Allocation by practice |
| Selective reporting | Unclear risk | No protocol identified |
| Other bias | Low risk |  |

Tamblyn 2012

| Methods | Cluster RCT, unit of allocation: healthcare professional  Summary risk of bias: high risk | |
| --- | --- | --- |
| Participants | Patients in the included study arms: patients >65 with a psychotropic drug prescription; female: 67.1%; mean age: 75.2; n= 5628 analysed  Professionals: 81 primary care physicians received the interventions  Setting: outpatient, Canada | |
| Interventions | Arm 1: CDS directed at the healthcare professional and delivered on screen supplemented with a risk of injury score when prescribing psychotropic medication  Am 2: standard CDS | |
| Outcomes | Continuous process measures: number of psychotropic drugs; psychotropic drug dose  Continuous patient measures: Risk of injury score (primary outcome)  Follow up period: mean 459 days | |
| Notes |  | |
| ***Risk of bias*** |  |  |
| **Bias** | **Authors’ judgement** | **Support for judgement** |
| Random sequence generation | Low risk | Random number table |
| Allocation concealment | Low risk | Randomised at the start by a statistician |
| Similar outcomes at baseline | Low risk | No important differences |
| Similar characteristics at baseline | Low risk | No important differences |
| Incomplete outcome data | Low risk | No loss to follow-up |
| Blinding of outcome assessment | Low risk | Objective outcome measures |
| Adequate protection against contamination | Unclear risk | Not clear if interactions between providers could have created contamination |
| Selective reporting | High risk | Protocol identified and deviations found |
| Other bias | Low risk |  |

Tierney 2005

| Methods | Cluster RCT, unit of allocation: practice sessions and pharmacists  Summary risk of bias: high risk | |
| --- | --- | --- |
| Participants | Patients in the included study arms: patients with asthma and chronic obstructive pulmonary disease; female: 72%; mean age: 51; n= 537 analysed  Professionals: 133 physicians received the included interventions  Setting: outpatient, United States | |
| Interventions | Arm 1: CDS directed at physician (delivered on screen) and pharmacist (delivered on paper)  Arm 2: CDS directed at pharmacist  Arm 3: CDS directed at physician | |
| Outcomes | Dichotomous process measures: % of CDS suggestions adhered to (primary outcome)  Dichotomous patient measures: % patients adherent with medication  Continuous patient measures: quality of life, number of all cause hospitalisations, number of emergency department visits  Economic measures: direct health care charges, number of all cause hospitalisations, number of emergency department visits  Satisfaction outcomes: patient satisfaction with physician, patient satisfaction with pharmacist  Follow up period: 12 months | |
| Notes | Data from control group not used | |
| ***Risk of bias*** |  |  |
| **Bias** | **Authors’ judgement** | **Support for judgement** |
| Random sequence generation | Unclear risk | A blinded statistician corrected the random allocation (by coin flip and by computer) |
| Allocation concealment | Unclear risk | Not clear if the randomisation was concealed |
| Similar outcomes at baseline | Unclear risk | Not clear |
| Similar characteristics at baseline | Low risk | No important differences |
| Incomplete outcome data | Low risk  (unclear risk for patient reported outcomes) | Limited and balanced loss to follow up for primary outcome and some of the secondary outcomes; larger but balanced loss to follow up for patient reported outcomes. The amount of loss is balanced, but the reasons are not clear. |
| Blinding of outcome assessment | Low risk  (unclear risk for patient reported outcomes) | The primary outcome and some of the secondary outcomes are objective; patient reported outcomes are subjective |
| Adequate protection against contamination | Unclear risk | Not clear |
| Selective reporting | Unclear risk | No protocol identified |
| Other bias | Low risk |  |

Turner 1989

| Methods | Cluster non randomised controlled trial, unit of allocation: clinic team  Summary risk of bias: high risk | |
| --- | --- | --- |
| Participants | Patients in the included study arms: participants with multiple chronic diseases; female: 72.1%; mean age: 60.4; n= 167 randomised  Professionals: 23 residents from 5 teams received the included interventions  Setting: outpatient, United States | |
| Interventions | Arm 1: CDS directed at the healthcare provider and delivered on paper, supplemented with patient questionnaires to screen the status of preventive care, and patient hand-outs on recommended care  Arm 2: standard CDS directed at the healthcare provider | |
| Outcomes | Dichotomous process measures: % patients with completed mammogram, % patients with completed breast exam, % patients with completed pap smear, % patients with % guaiac test, % patients with completed rectal exam, % patients with completed tetanus vaccinations  Follow up period: 5 months | |
| Notes | Source of funding is not clear; data for one trial arm not used | |
| ***Risk of bias*** |  |  |
| **Bias** | **Authors’ judgement** | **Support for judgement** |
| Random sequence generation | High risk | No randomisation |
| Allocation concealment | High risk | No randomisation |
| Similar outcomes at baseline | High risk | Important difference for mammograms, limited differences for other outcomes |
| Similar characteristics at baseline | Low risk | No important differences |
| Incomplete outcome data | Unclear risk | A sample of charts was audited |
| Blinding of outcome assessment | Low risk | Objective outcomes |
| Adequate protection against contamination | Low risk | Allocation by clinic teams |
| Selective reporting | Unclear risk | No protocol identified, detailed results for one outcome not provided |
| Other bias | Low risk |  |

Utidjian 2015

| Methods | Cluster RCT, unit of allocation: practice  Summary risk of bias: unclear risk | |
| --- | --- | --- |
| Participants | Patients in the included study arms: premature infants ; female: % not clear; age: 0-23 months; n= 146 analysed in the intervention season  Professionals: 20 primary care practices  Setting: outpatient, United States | |
| Interventions | Arm 1: CDS directed at nurses (delivered on screen) for one clinical target, supplemented with CDS directed at physicians for multiple clinical targets  Arm 2: CDS directed at nurses for one clinical target | |
| Outcomes | Dichotomous process measures: % eligible patients receiving Pavilizumab  Follow up period: 6 months | |
| Notes |  | |
| ***Risk of bias*** |  |  |
| **Bias** | **Authors’ judgement** | **Support for judgement** |
| Random sequence generation | Unclear risk | Randomisation method not clear |
| Allocation concealment | Unclear risk | Not clear |
| Similar outcomes at baseline | Low risk | No important differences |
| Similar characteristics at baseline | Unclear risk | Some differences present that might be important |
| Incomplete outcome data | Unclear risk | Not clear |
| Blinding of outcome assessment | Low risk | Objective outcomes |
| Adequate protection against contamination | Low risk | Allocation by practice |
| Selective reporting | Unclear risk | No protocol identified |
| Other bias | Low risk |  |

van Wijk 2001

| Methods | Cluster RCT, unit of allocation: practice  Summary risk of bias: high risk | |
| --- | --- | --- |
| Participants | Patients in the included study arms: patients requiring laboratory test ordering for miscellaneous conditions; female: 48.4%; mean age: 36.2; n= 25410 order forms completed during study  Professionals: general practitioners from 44 practices were included in the analysis  Setting: outpatient, the Netherlands | |
| Interventions | Arm 1: restricted list of lab tests relevant for a specific indication; directed at the healthcare provider (on screen)  Arm 2: restricted list lab tests that is generic for all indications | |
| Outcomes | Continuous process measures: number of blood tests ordered per order form per practice  Economic measures: number of blood tests ordered per order form per practice  Follow up period: 12 months | |
| Notes |  | |
| ***Risk of bias*** |  |  |
| **Bias** | **Authors’ judgement** | **Support for judgement** |
| Random sequence generation | Low risk | Random numbers table |
| Allocation concealment | Low risk | Allocation by a blinded researcher |
| Similar outcomes at baseline | High risk | Important baseline difference |
| Similar characteristics at baseline | Unclear risk | There was one baseline characteristic of the participating professionals that was different, it is unclear which impact this has |
| Incomplete outcome data | Low risk | Limited loss to follow-up that is balanced |
| Blinding of outcome assessment | Low risk | Objective outcome |
| Adequate protection against contamination | Low risk | Allocation on practice level |
| Selective reporting | Unclear risk | No protocol identified |
| Other bias | Low risk |  |

van Wyk 2008

| Methods | Cluster RCT, unit of allocation: practice  Summary risk of bias: unclear risk | |
| --- | --- | --- |
| Participants | Patients in the included study arms: patients from 18-75 years with miscellaneous conditions; female: 65,3% (requiring screening) and 59,4% (requiring treatment); mean age: 60 (requiring screening) and 58,4 (requiring treatment); n= 2328 analysed (requiring screening) and n=2187 analysed (requiring treatment)  Professionals: 80 general practitioners from 38 practices received the intervention  Setting: outpatient, the Netherlands | |
| Interventions | Arm 1: CDS provided automatically; directed at healthcare professionals and delivered on screen  Arm 2: CDS provided on demand | |
| Outcomes | Dichotomous process measures: % of due patients screened; % of due patients treated  Follow up period: 12 months (in general) | |
| Notes | Data from control group not considered | |
| ***Risk of bias*** |  |  |
| **Bias** | **Authors’ judgement** | **Support for judgement** |
| Random sequence generation | Low risk | Random number table |
| Allocation concealment | Low risk | Randomisation by an external person who was blinded |
| Similar outcomes at baseline | Unclear risk | Not clear |
| Similar characteristics at baseline | Low risk | No important differences |
| Incomplete outcome data | Unclear risk | Data for 2 practices lost in arm 1 |
| Blinding of outcome assessment | Low risk | Objective outcomes |
| Adequate protection against contamination | Low risk | Randomisation by practice |
| Selective reporting | Low risk | Protocol registered and no selective reporting identified |
| Other bias | Low risk |  |

Vinker 2002

| Methods | Cluster RCT, unit of allocation: providers and patients  Summary risk of bias: unclear risk | |
| --- | --- | --- |
| Participants | Patients in the included study arms: patients from 50-75 years due for colorectal cancer screening; female: 52.2%; mean age: 61.3 (including the control group); n= 1402 analysed  Professionals: 6 general practitioners from 2 practices received the interventions  Setting: outpatient, Israel | |
| Interventions | Arm 1 (two intervention arms combined): CDS directed at patients; delivered by phone or letter  Arm 2: CDS directed at healthcare providers and delivered on screen  Arm 3: CDS directed at healthcare providers and delivered on paper | |
| Outcomes | Dichotomous process measures: % patients with a fecal occult blood test  Follow up period: 12 months | |
| Notes | Data from the control group not considered; Data combined for patient reminder groups; Additional info on the intervention obtained from the author | |
| ***Risk of bias*** |  |  |
| **Bias** | **Authors’ judgement** | **Support for judgement** |
| Random sequence generation | Unclear risk | Not clear |
| Allocation concealment | Unclear risk | Not clear |
| Similar outcomes at baseline | Low risk | No important differences |
| Similar characteristics at baseline | Unclear risk | Not clear |
| Incomplete outcome data | Low risk | No loss to follow-up |
| Blinding of outcome assessment | Low risk | Objective outcomes |
| Adequate protection against contamination | Unclear risk | Healthcare providers collaborated in the same practice, it is unclear if this lead to contamination |
| Selective reporting | Unclear risk | No protocol identified |
| Other bias | Low risk |  |

Willis 2013

| Methods | Cluster RCT, unit of allocation: family  Summary risk of bias: unclear risk | |
| --- | --- | --- |
| Participants | Patients in the included study arms: patients with asthma, diabetes, hypertension, congestive heart failure, ischemic heart disease or stroke; female: 59.2%; mean age: not clear; n= 1480 analysed in the included arms  Professionals: primary healthcare providers, numbers are not clear  Setting: outpatient, United States | |
| Interventions | Arm 1: CDS directed at the healthcare provider and delivered on screen combined with care manager strategy Arm 2: standard CDS | |
| Outcomes | Dichotomous process measures: % patients with medication adherence (primary outcome)  Continuous patient measures: number of outpatient encounters per 100 patients, number of emergency department visits per 100 patients, number of hospitalisations per 100 patients  Economic measures: total medical costs  Follow up period: 18 months | |
| Notes | Data on control group not extracted | |
| ***Risk of bias*** |  |  |
| **Bias** | **Authors’ judgement** | **Support for judgement** |
| Random sequence generation | Low risk | Randomisation by a pseudo random number generator |
| Allocation concealment | Unclear risk | Not clear |
| Similar outcomes at baseline | Unclear risk | Not clear |
| Similar characteristics at baseline | Low risk | No important differences |
| Incomplete outcome data | Low risk | Limited loss to follow up |
| Blinding of outcome assessment | Low risk | Objective outcomes |
| Adequate protection against contamination | Unclear risk | Allocation by family and healthcare providers treated patients from both arms |
| Selective reporting | Low risk | Protocol registered and no selective reporting identified |
| Other bias | Low risk |  |

Ziemer 2006

| Methods | Cluster RCT, unit of allocation: clinic day  Summary risk of bias: high risk | |
| --- | --- | --- |
| Participants | Patients in the included study arms: patients with diabetes; female: 68%; mean age: 58.5; n= 2106 analysed in the included arms  Professionals: 345 residents received the interventions  Setting: outpatient, United States | |
| Interventions | Arm 1: CDS directed at the healthcare professionals and delivered on paper supplemented with performance feedback  Arm 2: standard CDS | |
| Outcomes | Dichotomous process measures: % visits where providers intisified the therapy, % visits where intensification of therapy met recommendations  Continuous patient measures: HbA1c values (primary outcome), systolic blood pressure, LDL cholesterol levels  Follow up period: 36 months | |
| Notes | Data for other trial arms not extracted | |
| ***Risk of bias*** |  |  |
| **Bias** | **Authors’ judgement** | **Support for judgement** |
| Random sequence generation | Unclear risk | Randomisation method not clear |
| Allocation concealment | Unclear risk | Not clear |
| Similar outcomes at baseline | Low risk | No important differences |
| Similar characteristics at baseline | Unclear risk | Difference in amount of consultations which might be important |
| Incomplete outcome data | Unclear risk | Potential loss to follow up for provider behaviour outcome |
| Blinding of outcome assessment | Low risk | Objective outcomes |
| Adequate protection against contamination | High risk | The study reports a high likelihood of contamination with frequent interactions between study groups |
| Selective reporting | High risk | Not all the planned outcomes were reported |
| Other bias | Low risk |  |
