# Supplementary material for: A systematic review of trials evaluating success factors of interventions with computerised clinical decision support
Source: Implement Sci. 2018 Aug 20;13:114. doi: 10.1186/s13012-018-0790-1 (PMC6102833; doi:10.1186/s13012-018-0790-1)
Supplement: Supplementary file 3 — Study results. (DOCX 95 kb) [file 13012_2018_790_MOESM3_ESM.docx]

# Additional file 3

# Detailed study results

Arts 2017

| **Comparison** | **Process measures** | **Patient measures** | **Other measures** |
| --- | --- | --- | --- |
| Arm 1 (CDS + override reason) vs arm 2 (standard CDS) | Dichotomous measure: % adherence to the CDS advice  Post intervention %: 53 vs 55  Difference: -2 (higher is better) | None | None |

Bates 1998

| **Comparison** | **Process measures** | **Patient measures** | **Other measures** |
| --- | --- | --- | --- |
| Arm 1 (CDS + multicomponent team intervention) vs arm 2 (standard CDS) | Continuous measure: rate of nonintercepted serious medication errors/100 patient days  Post intervention rate: 0.60 vs 0.48  Difference: +0.12 (lower is better)  Relative % change: +25  Standardised direction: -25 | None | None |

Becker 1990

| **Comparison** | **Process measures** | **Patient measures** | **Other measures** |
| --- | --- | --- | --- |
| Arm 1 (CDS for patient + provider) vs arm 2 (CDS for provider) | Dichotomous measure: % compliance with preventive care guidelines  Post intervention %: 18.5 vs 12.9  Difference: +5.6 (higher is better) | None | None |

Bloomfield 2005

| **Comparison** | **Process measures** | **Patient measures** | **Other measures** |
| --- | --- | --- | --- |
| Arm 1 (CDS before the visit for healthcare professional delivered as progress notes) vs arm 2 (CDS during visit for healthcare professional delivered as reminders) | Dichotomous measure: % patients with prescription of lipid lowering drugs  Post intervention %: 40.7 vs 39.4  Difference: 1.3 (higher is better) |  |  |

Bosworth 2011

| **Comparison** | **Process measures** | **Patient measures** | **Other measures** |
| --- | --- | --- | --- |
| Arm 1 (CDS + patient health behaviour programme) vs arm 2 (standard CDS) | None | Dichotomous measure: % with blood pressure in control (primary outcome)  (data extracted from a graphic)  Pre intervention %: 65 vs 52  Post intervention %: 70 vs 62  Adjusted difference: -5 (higher is better)  Continuous measure: mean systolic blood pressure  (data extracted from a graphic)  Pre intervention means: 127 vs 132  Post intervention mean: 123.6 vs 126.0  Adjusted difference: +2.6 (lower is better)  Relative % change: +2.1  Standardised direction: -2.1 | None |

Burack 1996a

| **Comparison** | **Process measures** | **Patient measures** | **Other measures** |
| --- | --- | --- | --- |
| Arm 1 (CDS for patient) vs arm 3 (CDS for provider) | Dichotomous measure: % women with mammography (primary outcome)  (data extracted from a graphic)  Pre intervention %: 19.9 vs 24.2  Post intervention %: 32.0 vs 29.1  Difference: +7.2 (higher is better)  Dichotomous measure: % women with visit to a physician  (data extracted from a graphic)  Pre intervention %: 38.9 vs 41.7  Post intervention %: 63.7 vs 64.7  Difference: +1.8 (higher is better)  Continuous measure: time to visit to a physician (Data not reported per group) | None | None |
| Arm 2 (CDS for provider + patient) vs arm 3 (CDS for provider) | Dichotomous measure: % women with mammography (primary outcome)  (data extracted from a graphic)  Pre intervention %: 21.5 vs 24.2  Post intervention %: 26.9 vs 29.1  Difference: +0.5 (higher is better)  Dichotomous measure: % women with visit to a physician  (data extracted from a graphic)  Pre intervention %: 39.5 vs 41.7  Post intervention %: 64.7 vs 64.7  Difference: +2.2 (higher is better)  Continuous measure: time to visit to a physician (Data not reported per group) |  |  |

Burack 1996b

| **Comparison** | **Process measures** | **Patient measures** | **Other measures** |
| --- | --- | --- | --- |
| Arm 1 (CDS for patient) vs arm 3 (CDS for provider) | Dichotomous measure: % women with mammography (primary outcome)  (data extracted from a graphic)  Pre intervention %: 9.3 vs 11.3  Post intervention %: 21.1 vs 36.0  Difference: -12.9 (higher is better)  Dichotomous measure: % women with visit to a physician  (data extracted from a graphic)  Pre intervention %: 46.9 vs 50.9  Post intervention %: 59.5 vs 56.3  Difference: +7.2 (higher is better)  Continuous measure: time to visit to a physician (Data not reported per group) | None | None |
| Arm 2 (CDS for provider + patient) vs Arm 3 (CDS for provider) | Dichotomous measure: % women with mammography (primary outcome)  (data extracted from a graphic)  Pre intervention %: 12.1 vs 11.3  Post intervention %: 36.0 vs 36.0  Difference: -0.8 (higher is better)  Dichotomous measure: % women with visit to a physician  (data extracted from a graphic)  Pre intervention %: 47.9 vs 50.9  Post intervention %: 55.5 vs 56.3  Difference: +2.2 (higher is better)  Continuous measure: time to visit to a physician (Data not reported per group) | None | None |

Burack 1998

| **Comparison** | **Process measures** | **Patient measures** | **Other measures** |
| --- | --- | --- | --- |
| Arm 1 (CDS for patient) vs arm 3 (CDS for provider) | Dichotomous measure: % patients with pap smear test (primary outcome)  Post intervention %: 29 vs 29  Difference: 0 (higher is better)  Dichotomous measure: % patients with visits  Post intervention %: 75 vs 77  Difference: -2 (higher is better) | None | Economic measures  % patients with visits (see left column) |
| Arm 2 (CDS for provider + patient) vs arm 3 (CDS for provider) | Dichotomous measure: % patients with pap smear test (primary outcome)  Post intervention %: 32 vs 29  Difference: +3 (higher is better)  Dichotomous measure: % patients with visits  Post intervention %: 79 vs 77  Difference: +2 (higher is better) | None | Economic measures  % patients with visits (see left column) |

Carroll 2013

| **Comparison** | **Process measures** | **Patient measures** | **Other measures** |
| --- | --- | --- | --- |
| Arm 1 (tailored CDS + patient hand-outs) vs Arm 2 (tailored CDS) | Dichotomous measure: % mothers with suspected maternal depression and therefore referred (primary outcome)  Post intervention %: 2.4 vs 2.4  Difference: 0.0 (higher is better) | Dichotomous measure: % mothers screened positive for depressed mood  Post intervention %: 8.7 vs 8.8  Difference: -0.1 (higher is better)  Dichotomous measure: % mothers screened positive for anhedonia  Post intervention %: 5.2 vs 5.1  Difference: +0.1 (higher is better)  Ranking of effects: -0.1, +0.1  Median effect size value: 0.0 (IQR -0.1 to +0.1) | None |
| Arm 2 (tailored CDS) vs arm 3 (standard CDS) | Dichotomous measure: % mothers with suspected maternal depression and therefore referred (primary outcome)  Post intervention %: 2.4 vs 1.2  Difference: +1.2 (higher is better) | Dichotomous measure: % mothers screened positive for depressed mood  Post intervention %: 8.8 vs 1.2  Difference: +7.6 (higher is better)  Dichotomous measure: % mothers screened positive for anhedonia  Post intervention %: 5.1 vs 0.4  Difference: +4.7 (higher is better)  Ranking of effects: +4.7, +7.6  Median effect size value: +6.2 (IQR +4.7 to +7.6) | None |

Chambers 1991

| **Comparison** | **Process measures** | **Patient measures** | **Other measures** |
| --- | --- | --- | --- |
| Arm 1 (CDS for all patients) vs arm 2 (CDS for half of the patients) | Dichotomous measure: % patients with influenza vaccination  Post intervention %: 50.6 vs 28.8  Difference: +21.8 (higher is better) | None | None |

Christakis 2004

| **Comparison** | **Process measures** | **Patient measures** | **Other measures** |
| --- | --- | --- | --- |
| Arm 1 (CDS for scheduler) vs arm 2 (CDS for provider) | Continuous measure: mean continuity of care score  Pre intervention means: 0.132 vs 0.144  Post intervention mean: 0.151 vs 0.158  Adjusted difference: +0.005 (higher is better)  Relative % change: +3.2 | None | None |
| Arm 1 (CDS for both scheduler and provider) vs arm 3 (CDS for provider) | Continuous measure: mean continuity of care score  Pre intervention means: 0.132 vs 0.144  Post intervention mean: 0.146 vs 0.158  Adjusted difference: 0.0 (higher is better)  Relative % change: 0.0 |  |  |

Del Fiol 2008

| **Comparison** | **Process measures** | **Patient measures** | **Other measures** |
| --- | --- | --- | --- |
| Arm 1 (CDS with specific content links) vs arm 2 (CDS with general overview links) | Dichotomous measure: % of infobutton searches where the needed information was found  Post intervention %: 83.7 vs 89.4  Difference: -5.7 (higher is better)  Continuous measure: mean score for the impact of the information seeking  Post intervention mean: 5.5 vs 5.3  Difference: +0.2 (higher is better)  Relative % change: +3.8  Continuous measure: median number of infobutton searches  Pre intervention median: 12 vs 11.5  Post intervention median: 22 vs 17.5  Continuous measure: amount of time (seconds) spent seeking information  Pre intervention median: 39.5 vs 40.5  Post intervention median: 35.5 vs 43 | None | None |

Denig 2014

| **Comparison** | **Process measures** | **Patient measures** | **Other measures** |
| --- | --- | --- | --- |
| Arm 1 and 2 combined (long CDS) vs arm 3 and 4 combined (short CDS) | Dichotomous measure: % patients with intensified glucose treatment  Odds ratio: 3.5 (95% CI 1.06 – 11.54) (higher is better)  Dichotomous measure: % patients with intensified blood pressure treatment  Odds ratio: 1.66 (95% CI 0.58 – 4.74) (higher is better)  Dichotomous measure: % patients with intensified lipid treatment  Odds ratio: 1.04 (95% CI 0.39 – 2.78) (higher is better)  Dichotomous measure: % patients with RAS inhibitors prescribed  Odds ratio: 1.51 (95% CI 0.48 – 4.73) (higher is better) | Continuous measure: mean score on diabetes empowerment scale (5 point scale, primary outcome)  Adjusted difference: -0.024 (higher is better) | None |
| Arm 1 and 3 combined (CDS on computer) vs arm 2 and 4 combined (CDS on paper) | Dichotomous measure: % patients with intensified glucose treatment  Odds ratio: 0.63 (95% CI 0.19 – 1.96) (higher is better)  Dichotomous measure: % patients with intensified blood pressure treatment  Odds ratio: 0.29 (95% CI 0.09 – 0.95) (higher is better)  Dichotomous measure: % patients with intensified lipid treatment  Odds ratio: 0.34 (95% CI 0.12 – 0.98) (higher is better)  Dichotomous measure: % patients with RAS inhibitors prescribed  Odds ratio: 0.67 (95% CI 0.21 – 2.10) (higher is better) | Continuous measure: mean score on diabetes empowerment scale (primary outcome)  Difference: -0.026 (higher is better) |  |

Derose 2005

| **Comparison** | **Process measures** | **Patient measures** | **Other measures** |
| --- | --- | --- | --- |
| Arm 1 (CDS + new recommend-dations) vs arm 2 (standard CDS) | Dichotomous measure: % with prescriptions of an ACEI or ARB and a statin within two weeks after a visit  Post intervention %: 7.6 vs 6.6  Difference: +1.0 (higher is better) | none | none |

Dexter 2004

| **Comparison** | **Process measures** | **Patient measures** | **Other measures** |
| --- | --- | --- | --- |
| Arm 1 (nurse directed CDS) vs arm 2 (physician directed CDS) | Dichotomous measure: % patients with pneumococcal vaccinations administered  Pre intervention %: 7.6 vs 7.6  Post intervention %: 51.5 vs 31.2  Adjusted difference: +20.3 (higher is better)  Dichotomous measure: % patients with influenza vaccinations administered  Pre intervention %: 19 vs 19  Post intervention %: 42.3 vs 29.6  Adjusted difference: +12.7 (higher is better)  Ranking of effects: +12.7, +20.3  Median effect size value: +16.5 (IQR +12.7 to +20.3 ) | None | None |

Dickinson 1981

| **Comparison** | **Process measures** | **Patient measures** | **Other measures** |
| --- | --- | --- | --- |
| Arm 1 (CDS + physician education) vs arm 2 (standard CDS) | None | Dichotomous measure: % patients with controlled diastolic blood pressure  Pre intervention %: 55 vs 41  Post intervention %: 80 vs 71  Adjusted difference: -5 (higher is better)  Dichotomous measure: % patients with improved diastolic blood pressure  Post intervention %: 57 vs 65  Difference: -8 (higher is better)  Dichotomous measure: % patients with improved systolic blood pressure  Post intervention %: 67 vs 71  Difference: -4 (higher is better)  Ranking of effects: -8, -5, -4  Median effect size value for dichotomous measures: -5 (IQR -8 to -4 )  Continuous measure: mean diastolic blood pressure  Pre intervention means: 88 vs 92  Post intervention mean: 84 vs 86  Adjusted difference: +2 (lower is better)  Relative % change: +2.3.  Standardised direction: -2.3  Continuous measure: mean systolic blood pressure  Pre intervention means: 162 vs 157  Post intervention mean: 149 vs 145  Adjusted difference: -1 (lower is better)  Relative % change: -0.7  Standardised direction: +0.7 | Economic measures  Continuous measure: mean appointments per patient  No data available for this comparison |

Duke 2013

| **Comparison** | **Process measures** | **Patient measures** | **Other measures** |
| --- | --- | --- | --- |
| Arm 1 (CDS + patient specific data) vs arm 2 (standard CDS) | Dichotomous measure: % adherence with CDS messages  Post intervention %: 14.6 vs 18.6  Difference: -4.0 (higher is better) | None | None |

El-Kareh 2011

| **Comparison** | **Process measures** | **Patient measures** | **Other measures** |
| --- | --- | --- | --- |
| Arm 1 (CDS linked to order entry) vs arm 2 (standard CDS) | Dichotomous measure: % of overdue mammographies performed following CDS  Pre intervention %: 7.4 vs 11.5  Post intervention %: 9.3 vs 14.3  Adjusted difference: -0.9 (higher is better)  Dichotomous measure: % of overdue bone-density scans performed following CDS  Pre intervention %: 4.2 vs 5.1  Post intervention %: 6.1 vs 5.8  Adjusted difference: +1.2 (higher is better)  Dichotomous measure: % of overdue HbA1c tests performed following CDS  Pre intervention %: 43.4 vs 54.0  Post intervention %: 48.2 vs 48.7  Adjusted difference: +10.1 (higher is better)  Dichotomous measure: % of overdue Low-density lipoprotein cholesterol tests performed following CDS  Pre intervention %: 33.6 vs 30.3  Post intervention %: 25.7 vs 20.8  Adjusted difference: +1.6 (higher is better)  Ranking of effects: -0.9, +1.2, +1.6, +10.1  Median effect size value: +1.4 (IQR +0.2 to +5.9) | None | None |

Feldman 2005

| **Comparison** | **Process measures** | **Patient measures** | **Other measures** |
| --- | --- | --- | --- |
| Arm 1 (CDS + multicomponent strategy) vs arm 2 (standard CDS) | None | Dichotomous measure: % with any hospitalisation  Post intervention %: 24.2 vs 30.3  Difference: -6.1 (lower is better)  Standardised direction: +6.1  Dichotomous measure: % with any emergency department visits  Post intervention %: 32.1 vs 28.2  Difference: +3.9 (lower is better)  Standardised direction: -3.9  Dichotomous measure: % with any outpatient doctor visit  Post intervention %: 85.1 vs 83.7  Difference: +1.4 (lower is better)  Standardised direction: -1.4  Ranking of effects: -3.9, -1.4, +6.1,  Median effect size value for dichotomous measures: - 1.4 (IQR -3.9 to +6.1)  Continuous measure: number of home care related visits  Post intervention mean: 44.1 vs 43.6  Difference: +0.5 (lower is better)  Relative % change: +1.2  Standardised direction: -1.2  Continuous measure: number of inpatient nights  Post intervention mean: 2.33 vs 1.97  Difference: +0.36 (lower is better)  Relative % change: +18.3  Standardised direction: -18.3  Continuous measure: number of emergency department visits  Post intervention mean: 0.55 vs 0.44  Difference: +0.11 (lower is better)  Relative % change: +25.0  Standardised direction: -25.0  Continuous measure: number of outpatient doctor visits  Post intervention mean: 2.62 vs 2.98  Difference: -0.36 (lower is better)  Relative % change: -12.1  Standardised direction: +12.1  Continuous measure: score on cardiomyopathy scale  Post intervention mean: 45.6 vs 46.6  Difference: -1.0 (higher is better)  Relative % change: -2.2  Continuous measure: score on depression scale  Post intervention mean: 36.9 vs 37.4  Difference: -0.5 (lower is better)  Relative % change: -1.3  Standardised direction: +1.3  Continuous measure: score on health related quality of life scale  Post intervention mean: 40.2 vs 48.9  Difference: -8.7 (higher is better)  Relative % change: -17.8 | **Economic measures**  Overall costs: 6330$ vs 5869$  Home care-related costs: 3425$ vs 3371$  Cost to produce a 5% improvement on cardiomyopathy scale: 235$ vs 183$ (home care related costs) and 513$ vs 246$ (overall costs)  Number of home care related visits, number of emergency department visits, number of outpatient doctor visits: see middle column |

Feldstein 2006a

| **Comparison** | **Process measures** | **Patient measures** | **Other measures** |
| --- | --- | --- | --- |
| Arm 1 (CDS directed at provider and patient) vs arm 2 (CDS directed at provider) | Dichotomous measure: % patients that received a pharmacological treatment or bone mineral density measurement (primary outcome)  Pre intervention %: 0 vs 0  Post intervention %: 33.0 vs 35.7  Adjusted difference: -2.7 (higher is better) | Continuous measure: calcium intake mg/day  Pre intervention means: 1221.5 vs 1116.5  Post intervention mean: 1224.7 vs 1311.4  Adjusted difference: -191.7 (higher is better)  Relative % change: -14.6 | None |

Feldstein 2006b

| **Comparison** | **Process measures** | **Patient measures** | **Other measures** |
| --- | --- | --- | --- |
| Arm 1 (CDS combined with academic detailing) vs arm 2 (standard CDS) | Continuous measure: Number of co-prescriptions of warfarin-interacting medications/10000 warfarin users/ month  Pre intervention rate: 2812 vs 3259  Post intervention rate: Significant difference (p=.002) with better results in arm 2 (no effect sizes reported) | None | None |

Feldstein 2006c

| **Comparison** | **Process measures** | **Patient measures** | **Other measures** |
| --- | --- | --- | --- |
| Arm 1 (CDS directed at patient) vs arm 2 (CDS directed at healthcare professional) | Dichotomous measure: % patients with completed baseline laboratory monitoring (primary outcome)  Pre intervention %: 0 vs 0  Post intervention %: 66.3 vs 48.5  Adjusted difference: +17.8 (higher is better) | Dichotomous measure: % of patients with abnormal test results  Post intervention %: 8.4 vs 5.1  Difference: +3.3 (higher is better) | None |

Folks 2011

| **Comparison** | **Process measures** | **Patient measures** | **Other measures** |
| --- | --- | --- | --- |
| Arm 1 (CDS for physician + academic detailing + CDS for patient) vs arm 2 (CDS for physician + academic detailing) | Dichotomous measure: % of patients without cardiovascular disease that receive aspirin  Pre intervention %: 60.7 vs 67.5  Post intervention %: 59.6 vs 66.4  Adjusted difference: 0.0 (lower is better) | None | None |

Forrest 2013

| **Comparison** | **Process measures** | **Patient measures** | **Other measures** |
| --- | --- | --- | --- |
| Arm 1 (CDS + monthly feedback) vs arm 2 (standard CDS) | Data on comparison arm 1 vs arm 2 is not reported in the paper | None | None |

Fortuna 2009

| **Comparison** | **Process measures** | **Patient measures** | **Other measures** |
| --- | --- | --- | --- |
| Arm 1 (CDS + education) vs arm 2 (standard CDS) | Dichotomous measure: % prescriptions for heavily marketed hypnotics (primary outcome)  Pre intervention %: 14.1 vs 12.7  Post intervention %: 12.1 vs 15.5  Adjusted difference: -4.8 (lower is better)  Standardised direction: +4.8  Dichotomous measure: % prescriptions changed in response to CDS  Data is only available for both groups together | None | None |

Fricton 2011

| **Comparison** | **Process measures** | **Patient measures** | **Other measures** |
| --- | --- | --- | --- |
| Arm 1 (CDS for patient and provider) vs arm 2 (CDS for provider) | Dichotomous measure: % guideline website users  Pre intervention %: 67 vs 71  Post intervention %: 55 vs 65  Adjusted difference: -6 (higher is better)  Dichotomous measure: % guideline website visits for patients with the targeted medical condition  Pre intervention %: 2 vs 0  Post intervention %: 34 vs 62  Adjusted difference: -30 (higher is better)  Continuous measure: mean guideline website visits per provider  Pre intervention means: 1.76 vs 1.71  Post intervention mean: 1.61 vs 2.58  Adjusted difference: -1.02 (higher is better)  Relative % change: -39.5 | None | None |

Hendrix 2015

| **Comparison** | **Process measures** | **Patient measures** | **Other measures** |
| --- | --- | --- | --- |
| CDS highlighted vs CDS not highlighted  (Each arm contained both highlighted and not highlighted CDS in an alternated way) | Dichotomous measure: % responded in any way to CDS (brush teeth for older children)  Post intervention %: 68 vs 65  Difference: + 3 (higher is better with highlighting)  Dichotomous measure: % responded in any way to CDS (brush teeth for younger children)  Post intervention %: 72 vs 58  Difference: +14 (higher is better with highlighting)  Dichotomous measure: % responded to CDS in any way (alcohol high risk)  Post intervention %: 0 vs 33  Difference: -33 (higher is better with highlighting)  Dichotomous measure: % responded to CDS in any way (alcohol low risk)  Post intervention %: 46 vs 50  Difference: -4 (higher is better with highlighting)  Dichotomous measure: % responded to CDS in any way (drugs high risk)  Post intervention %: 75 vs 0  Difference: +75 (higher is better with highlighting)  Dichotomous measure: % responded to CDS in any way (drugs low risk)  Post intervention %: no CDS triggered vs 100%  Difference: not applicable  Dichotomous measure: % responded to CDS in any way (injury burns 6m-6y)  Post intervention %: 44 vs 66  Difference: -22 (higher is better with highlighting)  Dichotomous measure: % responded to CDS in any way (injury burns 6y-12y)  Post intervention %: 56 vs 41  Difference: +15 (higher is better with highlighting)  Ranking of effects: -33,-22, -4, +3, +14, +15, +75  Median effect size value: +3 (IQR -22 to +15) | None | None |

Heiman 2004

| **Comparison** | **Process measures** | **Patient measures** | **Other measures** |
| --- | --- | --- | --- |
| Arm 1 (CDS for provider + patient directed information) vs arm 2 (CDS for provider) | Dichotomous measure: % patients with a completed advance directive  Pre intervention %: 0 vs 0  Post intervention %: 10.6 vs 1.3  Adjusted difference: +9.3 (higher is better) | None | None |

Kenealy 2005

| **Comparison** | **Process measures** | **Patient measures** | **Other measures** |
| --- | --- | --- | --- |
| Arm 1 (CDS for provider + patient completed screening form) vs arm 2 (CDS for provider) | Dichotomous measure: % patients screened for diabetes  Pre intervention %: 0 vs 0  Post intervention %: 23.7 vs 31.8  Adjusted difference: -8.1 (higher is better) | None | None |

Litzelman 1993

| **Comparison** | **Process measures** | **Patient measures** | **Other measures** |
| --- | --- | --- | --- |
| Arm 1 (CDS + override reasons) vs arm 2 (standard CDS) | Dichotomous measure: % compliance with CDS  Post intervention %: 46 vs 38  Difference: +8 (higher is better) | None | None |

Lobach 2013

| **Comparison** | **Process measures** | **Patient measures** | **Other measures** |
| --- | --- | --- | --- |
| Arm 1 (CDS for patient) vs arm 2 and 3 combined (CDS for care manager, CDS for clinic administrator) | Continuous measure: missed appointments/100 patients  Post intervention rate: 41.3 vs 36.1  Difference: +5.2 (lower is better)  Relative % change: +14.4  Standardised direction: -14.4 | Dichotomous measure: % rating excellent or very good on general health status scale (children and adults)  Post intervention %: 46.2 vs 54.3  Difference: -8.1 (higher is better)  Continuous measure: emergency department encounters/100 patients (primary outcome)  Post intervention rate: 33.5 vs 29.2  Difference: +4.3 (lower is better)  Relative % change: +14.7  Standardised direction: -14.7  Continuous measure: outpatient encounters/100 patients  Post intervention rate: 1617 vs 1393  Difference: +224 (lower is better)  Relative % change: +16.1  Standardised direction: -16.1  Continuous measure: hospitalisations/100 patients  Post intervention rate: 3.5 vs 3.4  Difference: +0.1 (higher is better)  Relative % change: +2.9  Continuous measure: general health status in adults (EQ-5D)  Post intervention mean: 59.60 vs 68.5  Difference: -8.9 (higher is better)  Relative % change: -13.0 | **Economic measures**  Total medical costs: 3077$ vs 2452$  Continuous measure: care manager contacts/100 patients  Post intervention rate: 12.9 vs 25.6  Emergency department encounters, outpatient encounters, hospitalisations: see middle column  **Satisfaction measures**  Provider ratings (0-10): 9.57 vs 9.43 among children (difference: +0.14 \| higher is better) and 9.33 vs 9.30 among adults (difference: +0.03 \| higher is better)  Healthcare ratings (0-10): 9.00 vs 9.10 among children (difference: - 0.1 \| higher is better) and 8.77 vs 8.64 among adults (difference + 0.13 \| higher is better) |

Loo 2011

| **Comparison** | **Process measures** | **Patient measures** | **Other measures** |
| --- | --- | --- | --- |
| Arm 1 (CDS + panel manager) vs arm 2 (standard CDS) | Dichotomous measure: % patients with healthcare proxy designation  Pre intervention %: 10.3 vs 11.6  Post intervention %: 27.9 vs 19.4  Adjusted difference: +9.8 (higher is better)  Dichotomous measure: % patients with bone density screening  Pre intervention %: 55.0 vs 47.8  Post intervention %: 68.8 vs 58.0  Adjusted difference: +3.6 (higher is better)  Dichotomous measure: % patients with peumococcal vaccination  Pre intervention %: 28.9 vs 30.0  Post intervention %: 47.1 vs 43.6  Adjusted difference: +4.6 (higher is better)  Dichotomous measure: % patients with influenza vaccination  Pre intervention %: 63.5 vs 63.5  Post intervention %: 59.7 vs 56.5  Adjusted difference: +3.2 (higher is better)  Ranking of effects: +3.2, +3.6, +4.6, +9.8  Median effect size value: +4.1 (IQR +3.4 to +7.2) | None | None |

Manns 2012

| **Comparison** | **Process measures** | **Patient measures** | **Other measures** |
| --- | --- | --- | --- |
| Arm 1 (CDS + specific recom-mendations) vs arm 2 (standard CDS) | Dichotomous measure: % with ACEi or ARB use among elderly chronic kidney disease patients with diabetes or proteinuria (primary outcome)  Pre intervention %: 77.0 vs 76.8  Post intervention %: 76.9 vs 77.1  Adjusted difference: -0.4 (higher is better)  Dichotomous measure: % patients with prescription of cholesterol-lowering medication (overall)  Post intervention %: 49.4 vs 47.7  Difference: +1.7 (higher is better)  Dichotomous measure: % patients with prescription of new antihypertensive medication from another class (overall)  Post intervention %: 26.6 vs 26.8  Difference: -0.2 (higher is better)  Dichotomous measure: % patients with consultation with a nephrologist (overall)  Post intervention %: 2.3 vs 2.4  Difference: -0.1 (higher is better)  Dichotomous measure: % patients with urinary albumin measurement (overall)  Post intervention %: 7.4 vs 6.3  Difference: +1.1 (higher is better)  Dichotomous measure: % patients with lipid measurement (overall)  Post intervention %: 21.7 vs 20.7  Difference: +1.0 (higher is better)  Dichotomous measure: % patients with A1C measurement (overall)  Post intervention %: 35.9 vs 37.9  Difference: -2.0 (higher is better)  Ranking of effects: -2.0,-0.2, -0.1, 1, 1.1, 1.7  Median effect size value: +0.45 (IQR -0.2 to +1.1) | Dichotomous measure: % with a composite clinical outcome (overall)  Post intervention %: 14.0 vs 13.3  Difference: +0.7 (lower is better)  Standardised direction: -0.7 | None |

McAlister 2009

| **Comparison** | **Process measures** | **Patient measures** | **Other measures** |
| --- | --- | --- | --- |
| Arm 1 (CDS signed by opinion leader) vs arm 2 (standard CDS) | Dichotomous measure: % with initiation of statin or increase of dose (primary outcome)  Post intervention %: 60.0 vs 54.0  Difference: +6.2 (higher is better)  Dichotomous measure: % taking a statin  Pre intervention %: 67.3 vs 63.9  Post intervention %: 83.6 vs 88.0  Adjusted difference: -7.8 (higher is better)  Dichotomous measure: % taking a nonstatin lipid-lowering drug  Pre intervention %: 7.3 vs 8.9  Post intervention %: 12.1 vs 13.9  Adjusted difference: -0.2 (higher is better)  Dichotomous measure: % taking any antiplatelet agent  Pre intervention %: 84.9 vs 89.2  Post intervention %: 88.5 vs 87.3  Adjusted difference: +5.5 (higher is better)  Dichotomous measure: % taking ACE inhibitor or ARB  Pre intervention %: 53.9 vs 53.8  Post intervention %: 64.8 vs 59.5  Adjusted difference: +5.2 (higher is better)  Dichotomous measure: % taking a β-blocker  Pre intervention %: 63.6 vs 66.5  Post intervention %: 76.4 vs 79.8  Adjusted difference: -0.5 (higher is better)  Dichotomous measure: % taking triple therapy  Pre intervention %: 33.9 vs 35.4  Post intervention %: 55.2 vs 53.8  Adjusted difference: +2.9 (higher is better)  Dichotomous measure: % received smoking cessation advice or prescribed smoking cessation product  Post intervention %: 44.8 vs 37.9  Difference: +6.9 (higher is better)  Ranking of effects: -7.8, -0.5, -0.2, +2.9, +5.2, +5.5, +6.9  Median effect size value for secondary dichotomous measures: + 2.9 (IQR -0.5 to +5.5)  Continuous measure: standardised mean statin dose  Pre intervention mean: 31 vs 31  Post intervention mean: 43 vs 42  Adjusted difference: +1 (higher is better)  Relative % change: +2.4 | Dichotomous measure: % patients stopped smoking  Post intervention %: 37.9 vs 31.0  Difference: +6.9 (higher is better)  Dichotomous measure: % with fasting LDL ≤ 2.0 mmol/L  Post intervention %: 44.8 vs 34.8  Difference: +10.0 (higher is better)  Dichotomous measure: % with emergency department visits  Post intervention %: 18.8 vs 22.8  Difference: -4.0 (lower is better)  Standardised direction: +4.0  Dichotomous measure: % with hospitalisations  Post intervention %: 32.7 vs 33.5  Difference: -0.8 (lower is better)  Standardised direction: +0.8  Dichotomous measure: % of deaths  Post intervention %: 0.61 vs 0  Difference: +0.61 (lower is better)  Standardised direction: -0.61  Ranking of effects: -0.61, +0.8, +4.0, +6.9, +10.0  Median effect size value for secondary dichotomous measures: +4.0 (IQR +0.1 to +8.5) | **Economic measures**  % with emergency department visits, % with hospitalisations: see middle column |

McDonald 1980

| **Comparison** | **Process measures** | **Patient measures** | **Other measures** |
| --- | --- | --- | --- |
| Arm 1 (CDS + bibliographic citations) vs arm 2 (standard CDS) | Dichotomous measure: % compliance with CDS  Post intervention %: 40.9 vs 35.9  Difference: + 5.0 (higher is better) | None | None |

McDonald 2005

| **Comparison** | **Process measures** | **Patient measures** | **Other measures** |
| --- | --- | --- | --- |
| Arm 1 (CDS + multicomponent intervention) vs arm 2 (standard CDS) | Dichotomous measure: % patients receiving recommended nurse assessments  Differences ( for each assessment type): -5.8, -4.0,-3.8, -1.3, -0.9, +3.0, +4.8, +5.6 (higher is better)  Dichotomous measure: % patients receiving recommended nurse instruction  Differences (combined): -7.6, -2.8, +3.5, +4.9, +11.1 (higher is better)  (for each instruction type)  Dichotomous measure: % use of alternative treatments  Post intervention %: 15.9 vs 22.6  Difference: -6.7 (lower is better)  Standardised direction: +6.7  Ranking of effects: -7.6,-5.8, -4.0,-3.8, -2.8, -1.3, -0.9, +3.0, +3.5, +4.8, +4.9, +5.6, +6.7, +11.1  Median effect size value: +1.1 (IQR -3.8 to +4.9) | Dichotomous measure: % patients with hospitalisation  Post intervention %: 16.6 vs 22.1  Difference: -5.5 (lower is better)  Standardised direction: +5.5  Dichotomous measure: % patients with emergency department visits  Post intervention %: 33.5 vs 37.8  Difference: -4.3 (lower is better)  Standardised direction: +4.3  Dichotomous measure: % patients indicating high quality of life  Post intervention %: 15.2 vs 16.9  Difference: -1.7 (higher is better)  Dichotomous measure: % patients with severe pain  Post intervention %: 25.8 vs 32.0  Difference: -4.2 (lower is better)  Standardised direction: +4.2  Dichotomous measure: % patients with severe insomnia  Post intervention %: 32.8 vs 39.5  Difference: -6.7 (lower is better)  Standardised direction: +6.7  Dichotomous measure: % patients with severe constipation  Post intervention %: 12.0 vs 14.8  Difference: -2.8 (lower is better)  Standardised direction: +2.8  Ranking of effects: -1.7, +2.8, +4.2, +4.3, +5.5, +6.7  Median effect size value: +4.3 (IQR +2.8 to +5.5 )  Continuous measure: level of pain at its worst  Post intervention mean: 3.2 vs 3.6  Difference: -0.4 (lower is better)  Relative % change: -11.1  Standardised direction: +11.1  Continuous measure: level of pain on average  Post intervention mean: 3.1 vs 2.2  Difference: +0.9 (lower is better)  Relative % change: +40.9  Standardised direction: -40.9  Continuous measure: score on pain interference scale  Post intervention mean: 5.2 vs 5.8  Difference: -0.6 (lower is better)  Relative % change: -10.4  Standardised direction: +10.4 | **Economic measures**  Overall costs: 5611$ vs 5966$  Home care related costs: 2903$ vs 2789$  Home care related costs of a 10% reduction in pain and in probability of hospitalisation: no data available for both groups simultaneously  % patients with hospitalisation, % patients with emergency department visits: see middle column |

McPhee 1989

| **Comparison** | **Process measures** | **Patient measures** | **Other measures** |
| --- | --- | --- | --- |
| Arm 1 (CDS directed at provider and patient) vs arm 2 (CDS directed at provider) | Dichotomous measure: % patients with completed mammography  (data extracted from a graphic)  Post intervention %: 74.3 vs 48.6  Difference: +25.7 (higher is better)  Dichotomous measure: % patients with completed breast examination  (data extracted from a graphic)  Post intervention %: 80.2 vs 82.9  Difference: -2.7 (higher is better)  Ranking of effects: -2.7, +25.7  Median effect size value: +14.2 (IQR -2.7 to +25.7) | None | None |

Meeker 2016

| **Comparison** | **Process measures** | **Patient measures** | **Other measures** |
| --- | --- | --- | --- |
| Arm 1 (CDS + override reasons + performance feedback) vs arm 2 (CDS + override reasons) | Dichotomous measure: % of inappropriate antibiotic prescriptions  Pre intervention %: 25.6 vs 35.5  Post intervention %: 10.0 vs 16.0  Adjusted difference: +3.9 (lower is better)  Standardised direction: -3.9 | None | None |
| Arm 2 (CDS + override reasons) vs arm 3 (standard CDS) | Dichotomous measure: % of inappropriate antibiotic prescriptions  Pre intervention %: 35.5 vs 49.6  Post intervention %: 16.0 vs 30.2  Adjusted difference: -0.1 (lower is better)  Standardised direction: +0.1 | None | None |

Murray 2004

| **Comparison** | **Process measures** | **Patient measures** | **Other measures** |
| --- | --- | --- | --- |
| Arm 1 (CDS for physician + pharmacist) vs arm 3 (CDS for physician) | Dichotomous measure: % compliance with CDS advice  Post intervention %: 35 vs 29  Difference: +6 (higher is better) | Continuous measure: generic health-related quality of life (primary outcome)  Difference (for each subscale): -8,-8,-7,-7,-6,-6,-5,-3 (higher is better)  Relative % change (median): -10.9 (IQR -12.7 to -9)  Continuous measure: number of emergency visits  Post intervention mean: 1.01 vs 1.02  Difference: -0.01 (lower is better)  Relative % change: -1  Standardised direction: +1  Continuous measure: number of hospitalisations  Post intervention mean: 0.19 vs 0.25  Difference: -0.06 (lower is better)  Relative % change: -24  Standardised direction:+24  Continuous measure: systolic blood pressure  Pre intervention mean: 143 vs 143  Post intervention mean: 142 vs 144  Adjusted difference: -2 (lower is better)  Relative % change: -1.4  Standardised direction: +1.4  Continuous measure: diastolic blood pressure  Pre intervention mean: 76 vs 75  Post intervention mean: 77 vs 75  Adjusted difference: +1 (lower is better)  Relative % change: +1.3  Standardised direction: -1.3 | **Economic measures**  Total health care charges: 3122$ vs 6200$  Number of emergency visits, number of hospitalisations: see middle column |
| Arm 2 (CDS for pharmacist) vs arm 3 (CDS for physician) | Dichotomous measure: % compliance with CDS advice  Post intervention %: 25 vs 29  Difference: -4 (higher is better) | Continuous measure: generic health-related quality of life (primary outcome)  Difference (for each subscale): -5, -4, -4,-4, -3, -2, -2, +4 (higher is better)  Relative % change (median): -5.0 (IQR -6.7 to -3.9)  Continuous measure: mean number of emergency visits  Post intervention mean: 1.11 vs 1.02  Difference: +0.09 (lower is better)  Relative % change: +9  Standardised direction: -9  Continuous measure: mean number of hospitalisations  Post intervention mean: 0.25 vs 0.25  Difference: 0 (lower is better)  Relative % change: 0  Continuous measure: systolic blood pressure  Post intervention mean: 144 vs 143  Post intervention mean: 144 vs 144  Difference: -1 (lower is better)  Relative % change: -0.69  Standardised direction: +0.69  Continuous measure: diastolic blood pressure  Post intervention mean: 78 vs 75  Post intervention mean: 77 vs 75  Difference: -1 (lower is better)  Relative % change: -1.33  Standardised direction: +1.33 | **Economic measures**  Total health care charges: 5445$ vs 6200$  Number of emergency visits, number of hospitalisations: see middle column |

Nendaz 2010

| **Comparison** | **Process measures** | **Patient measures** | **Other measures** |
| --- | --- | --- | --- |
| Arm 1 (automatic CDS in electronic chart) vs arm 2 (CDS on demand on PDA requiring extra data input) | Dichotomous measure: % patients with adequate prescription decisions  Pre intervention %: 50.5 vs 66.0  Post intervention %: 56.2 vs 64.9  Adjusted difference: +6.8 (higher is better) | None | None |

Ornstein 1991

| **Comparison** | **Process measures** | **Patient measures** | **Other measures** |
| --- | --- | --- | --- |
| Arm 1 (CDS for provider and patient) vs arm 3 (CDS for provider) | Dichotomous measure: % patients with completed mammogram  Pre intervention %: 11.4 vs 20.6  Post intervention %: 27.1 vs 31.3  Adjusted difference: +5.0 (higher is better)  Dichotomous measure: % patients with completed pap smear  Pre intervention %: 40.0 vs 43.8  Post intervention %: 39.2 vs 39.3  Adjusted difference: +3.7 (higher is better)  Dichotomous measure: % patients with completed fecal occult blood test  Pre intervention %: 9.3 vs 18.1  Post intervention %: 27.0 vs 23.2  Adjusted difference: +12.6 (higher is better)  Dichotomous measure: % patients with completed cholesterol test  Pre intervention %: 19.5 vs 22.9  Post intervention %: 38.1 vs 35.2  Adjusted difference: +6.3 (higher is better)  Dichotomous measure: % patients with completed tetanus vaccinations  Pre intervention %: 23.4 vs 23.6  Post intervention %: 35.4 vs 34.2  Adjusted difference: +1.4 (higher is better)  Ranking of effects: +1.4, +3.7, +5.0, +6.3, +12.6  Median effect size value: +5.0 (IQR +2.6 to +9.5) | None | None |
| Arm 2 (CDS for patient) vs arm 3 (CDS for provider) | Dichotomous measure: % patients with completed mammogram  Pre intervention %: 18.2 vs 20.6  Post intervention %: 21.0 vs 31.3  Adjusted difference: -7.9 (higher is better)  Dichotomous measure: % patients with completed pap smear  Pre intervention %: 37.4 vs 43.8  Post intervention %: 35.3 vs 39.3  Adjusted difference: +2.4 (higher is better)  Dichotomous measure: % patients with completed fecal occult blood test  Pre intervention %: 14.7 vs 18.1  Post intervention %: 23.4 vs 23.2  Adjusted difference: +3.6 (higher is better)  Dichotomous measure: % patients with completed cholesterol test  Pre intervention %: 17.5 vs 22.9  Post intervention %: 31.1 vs 35.2  Adjusted difference: +1.3 (higher is better)  Dichotomous measure: % patients with completed tetanus vaccinations  Pre intervention %: 16.1 vs 23.6  Post intervention %: 25.6 vs 34.2  Adjusted difference: -1.1 (higher is better)  Ranking of effects: -7.9, -1.1, +1.3, +2.4, +3.6  Median effect size: +1.3 (IQR -4.5 to +3.0) | None | None |

Persell 2008

| **Comparison** | **Process measures** | **Patient measures** | **Other measures** |
| --- | --- | --- | --- |
| Arm 1 (CDS directed at provider through 2 channels and CDS directed at patients) vs arm 2 (CDS directed at provider through 1 channel) | Dichotomous measure: % patients with regular aspirin use  Pre intervention %: 20.8 vs 19.6  Post intervention %: 46.2 vs 39.3  Adjusted difference: +5.7 (higher is better) | None | None |

Rimer 1999

| **Comparison** | **Process measures** | **Patient measures** | **Other measures** |
| --- | --- | --- | --- |
| Arm 1 (CDS for the provider combined with CDS for the patient and telephone counselling) vs arm 2 (CDS for the provider) | Dichotomous measure: % patients compliant with screening among women with hysterectomies  Post intervention %: 51 vs 47  Difference: +4 (higher is better)  Dichotomous measure: % patients compliant with screening among women without hysterectomies  Post intervention %: 61 vs 52  Difference: +9 (higher is better)  Ranking of effects: +4, +9  Median effect size value: +6.5 (IQR +4 to +9) | None | None |

Robbins 2012

| **Comparison** | **Process measures** | **Patient measures** | **Other measures** |
| --- | --- | --- | --- |
| Arm 1 (interactive CDS with semi-automation and in multiple channels) vs arm 2 (standard CDS) | Continuous measure: event rate of patients without follow-up appointments over 6 months per 100 patient years  Post intervention rate: 20.6 vs 30.1  Difference: -9.5 (lower is better)  Relative % change: -31.6  Standardised direction: +31.6  Continuous measure: time to next scheduled appointment after CDS  Post intervention mean: 1.71 vs 3.48  Difference: -1.77 (lower is better)  Relative % change: -50.9  Standardised direction: +50.9 | Continuous measure: mean CD4 cell count increase (primary outcome)  Pre intervention means: 0.456 x10^9^ vs 0.468 x10^9^  Pre to post intervention change: 0.0053x10^9^ vs 0.0032x10^9^  Adjusted difference: +0.0021x10^9^ (higher is better)  Relative % change: +0.45  Continuous measure: event rate of grade 3 or 4 toxicity per 100 patient years  Post intervention rate: 4.8 vs 4.0  Difference: +0.8 (lower is better)  Relative % change: +20  Standardised direction: -20  Continuous measure: event rate of confirmed virological failure per 100 patient years  Post intervention rate: 7.4 vs 5.2  Difference: +2.2 (lower is better)  Relative % change: +42.3  Standardised direction: - 42.3 | None |

Rosenberg 2008

| **Comparison** | **Process measures** | **Patient measures** | **Other measures** |
| --- | --- | --- | --- |
| Arm 1 (CDS directed at provider and patient) vs arm 2 (CDS directed at provider) | Dichotomous measure: % compliance with the CDS advice  Pre intervention %: 29.0 vs 30.0  Post intervention %: 31.0 vs 28.9  Adjusted difference: +3.1 (higher is better) | None | None |

Rosenbloom 2005

| **Comparison** | **Process measures** | **Patient measures** | **Other measures** |
| --- | --- | --- | --- |
| Arm 1 (CDS automatically available) vs arm 2 (CDS on demand) | Dichotomous measure: access to the decision support adjusted for the number of decision support opportunities  Post intervention %: 0.12 vs 0.01  Difference: +0.11 (higher is better) | none | **Economic measures**  Expenditure per order-entry session: $403.1 vs $408.6 |

Rosser 1991

| **Comparison** | **Process measures** | **Patient measures** | **Other measures** |
| --- | --- | --- | --- |
| Arm 1 (CDS for patient) vs arm 2 (CDS for healthcare professional) | Dichotomous measure: % patients with due preventive procedures performed  Post intervention %: 42.0 vs 33.7  Difference: +8.3 (higher is better) | None | **Economic measures**  cost per blood pressure reading gained: $14.37 to $31.27 (according to salary level and type of patient reminder) vs $ 1.33 to $1.77 (according to varying physician salary)  cost per cervical screening gained: $4.38 to $14.23 (according to salary level and type of patient reminder) vs $ 5.88 to $11.75 (according to varying physician salary) |

Roumie 2006

| **Comparison** | **Process measures** | **Patient measures** | **Other measures** |
| --- | --- | --- | --- |
| Arm 1(CDS for provider + patient directed information) vs arm 2 (CDS for provider only) | Dichotomous measure: % patients with any changes in antihypertensive drugs  Post intervention %: 29.1 vs 28.5  Difference: +0.6 (higher is better) | Dichotomous measure: % patients with systolic blood pressure ≤140 (primary outcome)  Post intervention %: 45.3 vs 27.1  Difference: +18.2 (higher is better)  Dichotomous measure: % patients with diastolic blood pressure < 90  Post intervention %: 68.3 vs 58.7  Difference: +9.6 (higher is better)  Dichotomous measure: % patients with hospitalisations  Post intervention %: 5.3 vs 2.9  Difference: +2.4 (lower is better)  Standardised direction: -2.4  Dichotomous measure: % patients with deaths  Post intervention %: 0.9 vs 0.6  Difference: +0.3 (lower is better)  Standardised direction: -0.3  Ranking of effects: -2.4, -0.3, +9.6  Median effect size value for secondary dichotomous measures: -0.3 (IQR -2.4 to +9.6)  Continuous measure: systolic blood pressure  Pre intervention means: 156.3 vs 158  Post intervention mean: 138 vs 146  Adjusted difference: -6.3 (lower is better)  Relative % change: -4.3  Standardised direction: +4.3  Continuous measure: patient medication adherence  Pre intervention means: 0.83 vs 0.85  Post intervention mean: 0.88 vs 0.89  Adjusted difference: +0.01 (higher is better)  Relative % change: +1.1 | None |

Scheepers-Hoeks 2013

| **Comparison** | **Process measures** | **Patient measures** | **Other measures** |
| --- | --- | --- | --- |
| Arm 1 (CDS automatically provided) vs arm 1 (CDS on demand) | Dichotomous measure: % of CDS followed  Post intervention %: 41.0 vs 18.8  Difference: +22.2 (higher is better) | None | **Satisfaction measures**  Continuous measure: Score on provider satisfaction (5 point scale)  Post intervention mean: 3.7 vs 3  Difference: +0.7 (higher is better) |
| Arm 3 (CDS for pharmacist) vs arm 4 (CDS for physician) | Dichotomous measure: % of CDS followed Post intervention %: 32.8 vs 20.1  Difference: +12.7 (higher is better) | None | **Satisfaction measures**  Continuous measure: Score on provider satisfaction (5 point scale)  Post intervention mean: 4.3 vs 2.7  Difference: +1.6 (higher is better) |

Schwarz 2012

| **Comparison** | **Process measures** | **Patient measures** | **Other measures** |
| --- | --- | --- | --- |
| Arm 1 (tailored CDS + order set) vs arm 2 (standard CDS) | Dichotomous measure: % encounters with documented provision of family planning services when potential teratogens prescribed (primary outcome)  Pre intervention %: 23.3 vs 25.5  Post intervention %: 27.4 vs 30.2  Adjusted difference: -0.6 (higher is better)  Dichotomous measure: % with a potentially teratogenic prescription  Pre intervention %: 14.3 vs 14.2  Post intervention %: 13.5 vs 14.4  Adjusted difference: -1.0 (lower is better)  Standardised direction: +1.0 | None | **Satisfaction measures**  Continuous measure: Score on provider satisfaction  Post intervention median: 5 vs 8 (10 point scale) |

Sequist 2009

| **Comparison** | **Process measures** | **Patient measures** | **Other measures** |
| --- | --- | --- | --- |
| Arm 1 (CDS directed at physician and patient) vs arm 2(CDS directed at physician) | Dichotomous measure: % patients screened (primary outcome)  Pre intervention %: 0 vs 0  Post intervention %: 44.2 vs 39.6  Adjusted difference: +4.6 (higher is better) | Dichotomous measure: % patients with pathological findings  No data reported for this measure | None |

Simon 2000

| **Comparison** | **Process measures** | **Patient measures** | **Other measures** |
| --- | --- | --- | --- |
| Arm 1 (CDS + care managers) vs arm 2 (standard CDS) | Dichotomous measure: % taking antidepressants in low dose in 6 months  Post intervention %: 46.58 vs 43.16  (data extracted from a graphic)  Difference: +3.42 (higher is better)  Dichotomous measure: % taking antidepressants in moderate dose in 6 months  Post intervention %: 30.0 vs 21.58  (data extracted from a graphic)  Difference: + 8.42 (higher is better)  Ranking of effects: +3.42, +8.42  Median effect size value for dichotomous measures: +5.9 (IQR +3.4 to +8.4) | Dichotomous measure: % with a 50% decrease in depression score scale at 6 months  Post intervention %: 55.71 vs 44.17  Difference: + 11.54 (higher is better)  Dichotomous measure: % with a major depression at 6 months  Post intervention %: 8.33 vs 14.52  (data extracted from a graphic)  Difference: - 6.19 (lower is better)  Standardised direction: +6.19  Ranking of effects: +6.19, +11.54  Median effect size value for dichotomous measures: +8.9 (IQR +6.2 to +11.5)  Continuous measure: depression scale score at 6 months  Post intervention mean: 0.83 vs 0.97  (data extracted from a graphic)  Difference: -0.14 (lower is better)  Relative % change: -14.4  Standardised direction: +14.4  Continuous measure: Total number of outpatient visits after antidepressant prescription  Post intervention mean: 4.62 vs 4.40  Difference: +0.22 (lower is better)  Relative % change: +5.0  Standardised direction: -5.0 | **Economic measures**  Total depression treatment costs (mean costs) (primary outcome): 484$ vs 414$  Total health services costs (mean costs): 2327$ vs 1673$  Time in treatment costs (mean costs): 249$ vs 232$  Total number of outpatient visits after antidepressant prescription: see middle column |

Simon 2001a

| **Comparison** | **Process measures** | **Patient measures** | **Other measures** |
| --- | --- | --- | --- |
| Arm 1 (CDS for provider and patients suggesting referral to physician) vs arm 3 (CDS for provider) | Dichotomous measure: % patients with mammogram performed  Post intervention %: 19 vs 17  Difference: +2 (higher is better) | None | None |
| Arm 1 (CDS for provider and patients suggesting referral to direct access) vs arm 3 (CDS for provider) | Dichotomous measure: % patients with mammogram performed  Post intervention %: 20 vs 17  Difference: +3 (higher is better) | None | None |

Simon 2001b

| **Comparison** | **Process measures** | **Patient measures** | **Other measures** |
| --- | --- | --- | --- |
| Arm 1 (CDS for provider and patients suggesting referral to physician) vs arm 3 (CDS for provider) | Dichotomous measure: % patients with mammogram performed  Post intervention %: 11 vs 11  Difference: 0 (higher is better) | None | None |
| Arm 1 (CDS for provider and patients suggesting referral to direct access) vs arm 3 (CDS for provider) | Dichotomous measure: % patients with mammogram performed  Post intervention %: 14 vs 11  Difference: +3 (higher is better) | None | None |

Simon 2006

| **Comparison** | **Process measures** | **Patient measures** | **Other measures** |
| --- | --- | --- | --- |
| Arm 1 (CDS + group academic detailing) vs arm 2 (standard CDS) | Continuous measure: Quarterly rates of use of target medications to avoid/10000 patients  Pre intervention rates: 146.3 vs 150.2  Post intervention rates: 126.6 vs 137.2  Adjsuted difference: -6.7 (lower is better)  Relative % change: -4.9  Standardised direction: +4.9 | None | None |

Skinner 2015

| **Comparison** | **Process measures** | **Patient measures** | **Other measures** |
| --- | --- | --- | --- |
| Arm 1 (tailored CDS for healthcare professional and patient) vs arm 2 (standard CDS for healthcare professional and patient) | Dichotomous measure: % patients with risk-appropriate colorectal cancer testing (primary outcome)  Pre intervention %: 0 vs 0  Post intervention %: 47.9 vs 41.6  Adjusted difference: +6.3 (higher is better)  Dichotomous measure: % with any colorectal cancer testing  Pre intervention %: 0 vs 0  Post intervention %: 49.8 vs 43.8  Adjusted difference: +6.0 (higher is better) | None | None |

Strom 2010

| **Comparison** | **Process measures** | **Patient measures** | **Other measures** |
| --- | --- | --- | --- |
| Arm 1 (CDS with response required) vs arm 2 (standard CDS) | Dichotomous measure: % desired ordering responses after CDS  Post intervention %: 24.6 vs 27.5  Difference: -2.9 (higher is better) | None | None |

Subramanian 2004

| **Comparison** | **Process measures** | **Patient measures** | **Other measures** |
| --- | --- | --- | --- |
| Arm 1 (CDS + collection of extra patient data) vs arm 2 (standard CDS) | Dichotomous measure: % CDS suggestions adhered to  Post intervention %: 33 vs 30  Difference: +3 (higher is better) | Dichotomous measure: % patients with improvement in NYHA class  Post intervention %: 44 vs 36  Difference: +8 (higher is better)  Continuous measure: Mean change in generic health-related quality of life score (physical component)  Mean change: -0.6 vs 1.3  Difference: -1.9 (higher is better)  Relative % change: post intervention mean not known  Continuous measure: Mean change in generic health-related quality of life score (mental component)  Mean change: 3.7 vs 2.1  Difference: +1.6 (higher is better)  Relative % change: post intervention mean not known  Continuous measure: Mean number of all cause hospitalisations  Post intervention mean: 2.3 vs 1.7 Difference: +0.6 (lower is better)  Relative % change: +35.3  Standardised direction:-35.3  Continuous measure: Mean number of outpatient visits  Post intervention mean: 6.7 vs 7.1  Difference: -0.4 (lower is better)  Relative % change: -5.6  Standardised direction: +5.6 | **Economic measures**  All cause hospitalisations, outpatient visits: see middle column  **Satisfaction measures**  Change in mean patient satisfaction with physician: 0.1 vs 0.0  Difference: +0.1 (higher is better)  Change in mean patient satisfaction with most recent visit: 0.0 vs -0.2  Difference: +0.2 (higher is better) |

Tamblyn 2008

| **Comparison** | **Process measures** | **Patient measures** | **Other measures** |
| --- | --- | --- | --- |
| Arm 1 (CDS automatically) vs arm 2 (CDS on demand) | Dichotomous measure: % of prescribing problems seen  Post intervention %: 10.3 vs 0.92  Difference: +9.4 (higher is better)  Dichotomous measure: % of prescribing problems revised  Post intervention %: 1.25 vs 0.70  Difference: +0.55 (higher is better)  Continuous measure: prevalence of prescription problems (primary outcome)  Odds ratio: 1.03 (95% CI 0.80-1.32)  (data per group not available) | None | None |

Tamblyn 2012

| **Comparison** | **Process measures** | **Patient measures** | **Other measures** |
| --- | --- | --- | --- |
| Arm 1 (CDS + risk injury score) vs arm 2 (standard CDS) | Continuous measure: number of psychotropic drugs  Pre intervention mean: 1.25 vs 1.28  Post intervention mean: 0.62 vs 0.67  Adjusted difference: -0.02 (lower is better)  Relative % change: -3.0  Standardised direction: +3.0  Continuous measure: intermediate-acting benzodiazepines drug dose  Pre intervention mean: 1.10 vs 1.20  Post intervention mean: 0.66 vs 0.68  Adjusted difference: 0.08 (lower is better)  Relative % change: +11.8  Standardised direction: -11.8  Continuous measure: long-acting benzodiazepines drug dose  Pre intervention mean: 0.74 vs 0.81  Post intervention mean: 0.70 vs 0.88  Adjusted difference: -0.11 (lower is better)  Relative % change: -12.5  Standardised direction: +12.5  Continuous measure: antidepressants drug dose  Pre intervention mean: 1.06 vs 1.04  Post intervention mean: 1.07 vs 1.04  Adjusted difference: +0.01 (lower is better)  Relative % change: +1.0  Standardised direction: -1.0  Continuous measure: anticonvulsants drug dose  Pre intervention mean: 0.54 vs 0.51  Post intervention mean: 0.59 vs 0.51  Adjusted difference: +0.05 (lower is better)  Relative % change: +9.8  Standardised direction: -9.8  Continuous measure: antipsychotics drug dose  Pre intervention mean: 0.79 vs 0.59  Post intervention mean: 0.54 vs 0.44  Adjusted difference: -0.1 (lower is better)  Relative % change: -22.7  Standardised direction: +22.7  Continuous measure: intermediate potency opiates drug dose  Pre intervention mean: 0.50 vs 0.43  Post intervention mean: 0.56 vs 0.38  Adjusted difference: +0.11 (lower is better)  Relative % change: +29.0  Standardised direction: -29.0  Continuous measure: low potency opiates drug dose  Pre intervention mean: 0.89 vs 0.88  Post intervention mean: 0.49 vs 0.79  Adjusted difference: -0.31 (lower is better)  Relative % change: -39.2  Standardised direction: +39.2 | Continuous measure: Risk of injury score (primary outcome)  Pre intervention mean: 3.85 vs 4.03  Post intervention mean: 3.58 vs 3.77  Difference: -0.01 (lower is better)  Relative % change: -0.3  Standardised direction: +0.3 | None |

Tierney 2005

| **Comparison** | **Process measures** | **Patient measures** | **Other measures** |
| --- | --- | --- | --- |
| Arm 1 (CDS for physician and pharmacist) vs arm 3 (CDS for physician) | Dichotomous measure: % of CDS suggestions adhered to (primary outcome)  Post intervention %: 36.7 vs 32.3  Difference: +4.4 (higher is better) | Dichotomous measure: % patients adherent with medication  Post intervention %: 82 vs 81  Difference: +1 (higher is better)  Continuous measure: quality of life  Relative % change (summary for 8 subscales ): -19.4, -11.6, -9.2, -5.4, -5.3, -2.7, -2.0, 18.8 (higher is better)  Median effect size value: -5.4 (IQR -10.4 to -2.4)  Continuous measure: number of all cause hospitalisations  Post intervention mean: 0.4 vs 0.5  Difference: -0.1 (lower is better)  Relative % change: -20  Standardised direction: +20  Continuous measure: number of emergency department visits  Post intervention mean: 1.4 vs 1.4  Difference: 0 (lower is better)  Relative % change: 0 | **Economic measures**  Direct health care charges 5652$ vs 8006$  Number of all cause hospitalisations, number of emergency department visits: see middle column  **Satisfaction measures**  Patient satisfaction with physician: 2.1 vs 1.9  Difference: +0.2 (higher is better)  Patient satisfaction with pharmacist: 2.0 vs 2.1  Difference: -0.1 (higher is better) |
| Arm 2 (CDS for pharmacist) vs arm 3 (CDS for physician) | Dichotomous measure: % of CDS suggestions adhered to (primary outcome)  Post intervention %: 32.2 vs 32.3  Difference: -0.1 (higher is better) | Dichotomous measure: % patients adherent with medication  Post intervention %: 80 vs 81  Difference: -1 (higher is better)  Continuous measure: quality of life  Relative % change (summary for 8 subscales ): -21.6, -8.7, -7.7, -4.1, 0, 0, 3.1, 5.4 (higher is better)  Median effect size value: -2.1 (IQR -8.2 to +1.6)  Continuous measure: number of all cause hospitalisations  Post intervention mean: 0.5 vs 0.5  Difference: 0.0 (lower is better)  Relative % change: 0.0  Continuous measure: number of emergency department visits  Post intervention mean: 1.5 vs 1.4  Difference: +0.1 (lower is better)  Relative % change: +7.1  Standardised direction: -7.1 | **Economic measures**  Direct health care charges: 5333$ vs 8006$  Number of all cause hospitalisations, number of emergency department visits: see middle column  **Satisfaction measures**  Patient satisfaction with physician: 2.0 vs 1.9  Difference: +0.1 (higher is better)  Patient satisfaction with pharmacist: 2.1 vs 2.1  Difference: 0.0 (higher is better) |

Turner 1989

| **Comparison** | **Process measures** | **Patient measures** | **Other measures** |
| --- | --- | --- | --- |
| Arm 1 (CDS and patient questionnaire and patient information) vs arm 2 (standard CDS) | Dichotomous measure: % patients with completed mammogram  Pre intervention %: 0 vs 14.3  Post intervention %: 4.8 vs 15  Adjusted difference: +4.1 (higher is better)  Dichotomous measure: % patients with completed breast exam  Pre intervention %: 38.3 vs 37.5  Post intervention %: 47.8 vs 53.5  Adjusted difference: -6.5 (higher is better)  Dichotomous measure: % patients with completed pap smear  Pre intervention %: 20.6 vs 20.3  Post intervention %: 40 vs 33.1  Adjusted difference: +6.6 (higher is better)  Dichotomous measure: % patients with guaiac test  Pre intervention %: 29.7 vs 34.1  Post intervention %: 46.1 vs 50  Adjusted difference: +0.5 (higher is better)  Dichotomous measure: % patients with completed rectal exam  Pre intervention %: 39.1 vs 40.4  Post intervention %: 57 vs 52.6  Adjusted difference: +5.7 (higher is better)  Dichotomous measure: % patients with completed tetanus vaccinations  (no specific data provided)  Ranking of effects: -6.5, +0.5, +4.1, +5.7, +6.6  Median effect size value: +4.1 (IQR -3 to 6.2 ) | None | None |

Utidjian 2015

| **Comparison** | **Process measures** | **Patient measures** | **Other measures** |
| --- | --- | --- | --- |
| Arm 1 (nurse directed CDS supplemented with physician directed CDS for multiple clinical targets) vs arm 2 (nurse directed CDS directed for one clinical target) vs | Dichotomous measure: % eligible patients receiving Pavilizumab  Pre intervention %: 62.4 vs 65.5  Post intervention %: 67.9 vs 84.7  Adjusted difference: -13.7 (higher is better) | None | None |

Van Wijk 2001

| **Comparison** | **Process measures** | **Patient measures** | **Other measures** |
| --- | --- | --- | --- |
| Arm 1 (CDS with restricted order list that is specific) vs arm 2 (CDS with restricted order list that is generic) | Continuous measure: number of blood tests ordered per order form per practice  Pre intervention means: 7.2 vs 7.7  Post intervention mean: 5.5 vs 6.9  Adjusted difference: -0.9 (lower is better)  Relative % change: -13.0  Standardised direction: +13.0 | None | **Economic measure**  Number of blood tests ordered per order form per practice: See left column |

Van Wyk 2008

| **Comparison** | **Process measures** | **Patient measures** | **Other measures** |
| --- | --- | --- | --- |
| Arm 1 (CDS provided  automatically) vs  arm 2 (CDS on demand) | Dichotomous measure: % of due patients screened  Post intervention %: 65.0 vs 35.1  Difference: +29.9 (higher is better)  Dichotomous measure: % of due patients treated  Post intervention %: 65.7 vs 39.7  Difference: +26.0 (higher is better)  Ranking of effects: +26.0, +29.9  Median effect size value: +28.0 (IQR +26.0 to +29.9) | None | None |

Vinker 2002

| **Comparison** | **Process measures** | **Patient measures** | **Other measures** |
| --- | --- | --- | --- |
| Arm 1 (CDS directed at patient) vs arm 2 and 3 combined (CDS directed at provider | Dichotomous measure: % patients with a fecal occult blood test  Pre intervention %: 2.2 vs 2.2  Post intervention %: 16.5 vs 11.9  Adjusted difference: +4.6 (higher is better) | None | None |
| Arm 2 (CDS delivered on-screen) vs arm 3 (CDS delivered in print) | Dichotomous measure: % patients with a fecal occult blood test  Pre intervention %: 2.2 vs 2.2  Post intervention %: 25.4 vs 9.8  Adjusted difference: +15.6 (higher is better) | None | None |

Willis 2013

| **Comparison** | **Process measures** | **Patient measures** | **Other measures** |
| --- | --- | --- | --- |
| Arm 1 (CDS + care manager) vs arm 2 (standard CDS) | None | Dichotomous measure: % patients with medication adherence (primary outcome)  Post intervention %: 42.9 vs 41.2  Difference: +1.7 (higher is better)  Continuous measure: number of outpatient encounters per 100 patients  Post intervention rate: 44.5 vs 46.6  Difference: -2.1 (lower is better)  Relative % change: -4.5  Standardised direction: +4.5  Continuous measure: number of emergency department visits per 100 patients  Post intervention rate: 0.89 vs 0.84  Difference: +0.05 (lower is better)  Relative % change: +5.9  Standardised direction: -5.9  Continuous measure: number of hospitalisations per 100 patients  Post intervention rate: 0.21 vs 0.21  Difference: 0.0 (lower is better)  Relative % change: 0.0 | Economic measures  Total medical costs: data is not clear for one of the arms |

Ziemer 2006

| **Comparison** | **Process measures** | **Patient measures** | **Other measures** |
| --- | --- | --- | --- |
| Arm 1 (CDS + performance feedback) vs arm 2 (standard CDS) | Dichotomous measure: % visits where providers intensified the therapy  (data extracted from a graphic)  Pre intervention %: 33.1 vs 33.7  Post intervention %: 51.8 vs 38.6  Adjusted difference: +13.8 (higher is better)  Dichotomous measure: % visits where intensification of therapy met recommendations  (data extracted from a graphic)  Pre intervention %: 40.3 vs 42.5  Post intervention %: 50.5 vs 42.1  Adjusted difference: +8.4 (higher is better)  Ranking of effects: 8.4, 13.2  Median effect size value: +10.8 (IQR +8.4 to +13.2) | Continuous measure: HbA1c values (primary outcome)  Pre intervention means: 8.02 vs 7.99  Post intervention mean: 7.46 vs 7.69  Adjusted difference: -0.26 (lower is better)  Relative % change: -3.38  Standardised direction: +3.38  Continuous measure: systolic blood pressure  Pre intervention means: 138.36 vs 135.73  Post intervention mean: 134.96 vs 136.93  Adjusted difference: -4.6 (lower is better)  Relative % change: -3.36  Standardised direction: +3.36  Continuous measure: LDL cholesterol levels  Pre intervention means: 121.63 vs 119.24  Post intervention mean: 103.43 vs 104.01  Adjusted difference: -2.97 (lower is better)  Relative % change: -2.86  Standardised direction: +2.86 | None |
