# Supplementary material for: A systematic review of trials evaluating success factors of interventions with computerised clinical decision support
Source: Implement Sci. 2018 Aug 20;13:114. doi: 10.1186/s13012-018-0790-1 (PMC6102833; doi:10.1186/s13012-018-0790-1)
Supplement: Supplementary file 4 — Synthesis of results per factor. (DOCX 240 kb) [file 13012_2018_790_MOESM4_ESM.docx]

# Additional file 4

# Synthesis of results per factor and summary of findings-tables

**1. More versus less evidence-based CDS advice**

We identified two studies for this comparison.^1, 2^ McDonald 1980 compared CDS supplemented with bibliographic citations versus CDS advice without citations.^1^ Access to full text was available upon request. Denig 2014 compared CDS with treatment outcome information for one major clinical endpoint versus outcome information for other patient-important endpoints.^2^ The CDS was directed at both healthcare providers and patients to support shared decision-making.

Baseline data were not available so the studies did not qualify for the primary analysis.

*Secondary analysis*

McDonald 1980 found a 5% RD for compliance with recommended practice for miscellaneous conditions when providing bibliographic citations.^1^

Denig 2014 targeted patients with diabetes and found that the odds ratios for compliance ranged from 1.04 to 3.5 (data to present absolute increases were not available) when adding treatment outcome information for additional endpoints. There was little or no change in scores on the diabetes empowerment scale.^2^

*Certainty*

We appraised both studies as high risk of bias. The quality of the evidence according to GRADE was low for both process and patient outcomes.

*Summary of findings table*

| **Patient or population**: any patient **^1^**  **Settings**: any setting **^2^**  **Intervention**: CDS that is made more evidence based**^3^**  **Comparison**: CDS that does not present the additional information | | | |
| --- | --- | --- | --- |
| **Outcomes** | Result | **Nb of participants (studies)** | **Quality of the evidence (GRADE)** |
| *PRIMARY ANALYSIS* |  |  |  |
|  | - |  |  |
| *SECONDARY ANALYSIS* |  |  |  |
| Compliance with desired practice  (follow-up 15 weeks)  Changes in clinical outcomes (follow-up 6 months) | RD 5.0% **^4^**  % patients with intensified glucose treatment: Odds ratio 3.5 (95% CI 1.06 – 11.54), % patients with intensified blood pressure treatment: Odds ratio 1.66 (95% CI 0.58 – 4.74), % patients with intensified lipid treatment: Odds ratio 1.04 (95% CI 0.39 – 2.78), % patients with RAS inhibitors prescribed: Odds ratio 1.51 (95% CI 0.48 – 4.73)  Mean difference -0.024**^9^** | 31 care providers from one clinic (1 study**^6^**)  225 patients  (1 study**^7^**)  225 patients  (1 study**^7^**) | ⊕⊕🌕🌕  **Low^8^**  ⊕⊕🌕🌕  **Low^8^** |
| **^1^** The studies included patients with various conditions  **^2^** The studies were done in an outpatient settings in the US and the Netherlands  **^3^** The study interventions were CDS supplemented with bibliographic citations with the full text available upon request and the provision of additional treatment outcome information to clinicians and patients  **^4^** The outcome was % compliance with CDS  **^5^** The outcomes were % patients with intensified glucose treatment, intensified blood pressure treatment, intensified lipid treatment, % patients with RAS inhibitors prescribed  **^6^** McDonald 1980  **^7^** Denig 2014  **^8^** Downgraded for risk of bias and because the finding is limited to a single study  **^9^** The outcome was score on diabetes empowerment scale, the value is adjusted for baseline differences | | | |

**2. More versus less patient-specific CDS**

Five studies were available for this comparison.^3-7^ Four studies (Simon 2000, Skinner 2015, Subramanian 2004, Carroll 2013) compared CDS that provided more patient-specific advice based on additional patient data versus less patient-specific CDS in the comparison group.^3-5, 7^ van Wijk 2001 ^3, 6^compared indication-specific order sets with generic order sets.

*Primary analysis*

Skinner 2015 found a 6.3% absolute improvement in risk-appropriate colorectal cancer testing with more patient-specific CDS.^4^

*Secondary analysis*

Two studies on treatment of depression (Simon 2000) and heart failure (Subramanian 2004) and one study (Carroll 2013) on screening for postnatal depression found that compliance with recommended practice improved with an unadjusted RD of 3.0% (IQR +1.2 to +4.9).^3, 5, 7^ Van Wijk 2001 found that less blood tests were ordered with more patient specific CDS (13% relative change).^6^

Three studies found small to modest improvements in dichotomous patient outcomes with RD of 8% (IQR 0 to +8.9).^3, 5, 7^ Two studies showed varying results for continuous patient measures with both positive and negative findings in each study.^3, 5^

Simon 2000 found higher treatment costs (484$ vs 414$) and health services costs (2327$ vs 1673$) with the more patient-specific CDS.^3^ The results on resource use were conflicting with findings of both increased and reduced resource use.^3, 5, 6^ Subramanian 2004 measured patient satisfaction and found no meaningful difference.^5^

*Indirect evidence*

Schwarz 2012 also evaluated the effect of CDS that provided more patient-specific information.^8^ We did not include this study in the comparison since the intervention was combined with the provision of computerised order sets. This study found an adjusted RD of -0.6 % for encounters with documented provision of family planning services when potential teratogens were prescribed. Provider satisfaction was lower (median 5 versus 8 on a 10-point scale).

*Certainty*

Four studies had a high risk of bias^3, 4, 6, 7^ and one study (Subramanian 2004) had an unclear risk of bias.^5^ The risk of bias for Schwarz 2012 was unclear. Quality of the evidence according to GRADE was moderate for process outcomes and low for clinical outcomes.

*Summary of findings table*

| **Patient or population**: any patient **^1^**  **Settings**: any setting **^2^**  **Intervention**: CDS that is made more patient specific by using additional patient data**^3^**  **Comparison**: CDS that does not use the additional patient data | | | |
| --- | --- | --- | --- |
| **Outcomes** | Result | **Nb of participants (studies)** | **Quality of the evidence (GRADE)** |
| *PRIMARY ANALYSIS* |  |  |  |
| Compliance with desired practice (follow-up 12 months) | RD 6.2% **^4^** | 651 patients  (1 study**^5^**) | ⊕⊕⊕🌕  **Moderate^6^** |
| *SECONDARY ANALYSIS* |  |  |  |
| Compliance with desired practice  (median follow-up 12 months)  Changes in clinical outcomes (median follow-up 12 months) | RD 3.0% (IQR +1.2 to +4.9) **^7^**  13% relative change **^9^**  RD 8% (IQR 0 to +8.9) **^11^**  % relative change values: -35.3 (all cause hospitalisations), -5.0 (outpatient visits), +5.6 (outpatient visits) +14.4 (depression scale score) *(+ means improvement)* | 3468 patients (3 studies**^8^**)  44 family medicine practices (1 study**^10^**)  3468 patients (3 studies**^8^**)  1115 patients (2 studies**^13)^** | ⊕⊕⊕🌕  **Moderate^6^**  ⊕⊕🌕🌕  **Low^12^** |
| **^1^** The studies included patients potentially eligible for colon cancer screening, patients with a new prescription for antidepressants to treat depression, mothers at risk of depression, patients with heart failure and left ventricular systolic dysfunction, patients requiring laboratory test ordering  **^2^** The studies were done in outpatient settings in the US and the Netherlands  **^3^** The study intervention was CDS tailored to additional patient data  **^4^** The outcome was % patients with risk-appropriate colorectal cancer testing  **^5^** Skinner 2015  **^6^** Downgraded for risk of bias, data from the primary and secondary analysis correspond.  **^7^** The outcomes were % CDS suggestions adhered to, % taking antidepressants, % mothers with suspected maternal depression and therefore referred  **^8^** Simon 2000, Subramanian 2004, Carroll 2013  **^9^**  The outcome was number of blood tests ordered  **^10^** Van Wijk 2001  **^11^** The outcomes were % with a 50% decrease in depression score, % with a major depression, % mothers screened positive for depressed mood; % mothers screened positive for anhedonia, % patients with improvement in NYHA class for heart failure  **^12^** Downgraded for risk of bias, and inconsistency  **^13^** Simon 2000, Subramanian 2004 | | | |

**3. More versus less explicit CDS advice by providing recommendations**

Two studies (Derose 2005, Manns 2012) compared CDS that provided relevant patient data with clear recommendations versus CDS that presented relevant patient data but without explicit recommendations.^9, 10^

*Primary analysis*

Manns 2012 found that more explicit advice did not improve compliance with recommended practice for patients with chronic kidney disease (-0.4 % absolute change).

*Secondary analysis*

The unadjusted RD for compliance with recommended practice for patients with high cardiovascular risk was 1.0% in Derose 2005. Dichotomous patient outcomes were lower (unadjusted RD -0.7%) with more explicit advice in Manns 2012.

*Certainty*

Manns 2012 had a low risk of bias and Derose 2005 had a high risk of bias. Quality of the evidence according to GRADE was moderate for both process and patient outcomes.

*Summary of findings table*

| **Patient or population**: any patient **^1^**  **Settings**: any setting **^2^**  **Intervention**: CDS that is made more explicit by providing recommendations **^3^**  **Comparison**: CDS presenting patient data instead of recommendations | | | |
| --- | --- | --- | --- |
| **Outcomes** | Result | **Nb of participants (studies)** | **Quality of the evidence (GRADE)** |
| *PRIMARY ANALYSIS* |  |  |  |
| Compliance with desired practice (median follow-up 25 months) | RD -0.4% **^4^** | 5444 patients  (1 study**^5^**) | ⊕⊕⊕🌕  **Moderate^6^** |
| *SECONDARY ANALYSIS* |  |  |  |
| Compliance with desired practice  (follow-up 1 1.5 months)  Changes in clinical outcomes (median follow-up 25 months) | RD 1.0% **^7^**  RD -0.7% **^9^** | 8861 patients (1 study**^8^**)    22092 patients (1 study**^5^**) | ⊕⊕⊕🌕  **Moderate^6^**  ⊕⊕⊕🌕  **Moderate^6^** |
| **^1^** The studies included chronic kidney disease patients and patients with a high cardiovascular risk  **^2^** The studies were done in outpatient settings in the US and Canada  **^3^** The study interventions were presenting specific management recommendations  **^4^** The outcome was % with ACEi or ARB use among elderly chronic kidney disease patients with diabetes or proteinuria  **^5^**  Manns 2012  **^6^** Downgraded due to the limited amount of studies.  **^7^** The outcome was % with dispensed prescriptions of an ACEI or ARB and a statin  **^8^** Derose 2005  **^9^**  The outcome was % patients with composite clinical outcome (death, end-stage renal disease, doubling of serum creatinine, or hospitalization for myocardial infarction, heart failure, or stroke) | | | |

**4. More versus less explicit CDS advice by presenting specific patient data**

Two studies (Duke 2013, Tamblyn 2012) compared CDS drug alerts that were supplemented with patient data (relevant laboratory data, patient specific-risk estimates) to drug alerts that were not supplemented with patient specific data.^11, 12^ The studies focussed on drug alerts associated with hyperkalemia or with the prescription of psychotropic drugs.

No studies were included in the primary analysis.

*Secondary analysis*

Duke 2013 found that compliance with the CDS advice decreased (unadjusted RD -4.0 %). Tamblyn 2012 found that the amount of psychotropic drugs slightly improved (3.0% relative change), findings on changes in drug doses were conflicting and clinical outcomes did not change.

*Certainty*

Duke 2013 had a low risk of bias and Tamblyn 2012 had a high risk of bias. Quality of the evidence according to GRADE was low for both process and patient outcomes.

*Summary of findings table*

| **Patient or population**: any patient **^1^**  **Settings**: any setting **^2^**  **Intervention**: CDS that is made more explicit by presenting relevant patient data **^3^**  **Comparison**: CDS providing an alert without presentation of additional patient data | | | |
| --- | --- | --- | --- |
| **Outcomes** | Result | **Nb of participants (studies)** | **Quality of the evidence (GRADE)** |
| *PRIMARY ANALYSIS* |  |  |  |
|  | - |  |  |
| *SECONDARY ANALYSIS* |  |  |  |
| Compliance with desired practice  (follow-up 6 months)  Changes in clinical outcomes (median follow-up 25 months) | RD -4.0% **^4^**  3.0% relative improvement in number of psychotropic drugs  % relative change values for drug doses: -29.0(intermediate potency opiates), (-11.8 (intermediate-acting benzodiazepines), -9.8 (anticonvulsants), -1.0 (antidepressants), +12.5 (long-acting benzodiazepines), +22.7 (antipsychotics), +39.2 (low potency opiates) *(+ means improvement)*  +0.3% relative change in risk of injury score *(+ means improvement)* | 203 physicians (1 study**^5^**)  5628 patients (1 study**^7^**)  5628 patients (1 study**^7^**) | ⊕⊕🌕🌕  **Low^6^**  ⊕⊕🌕🌕  **Low^6^** |
| **^1^** The studies focussed on drug-drug interactions associated with hyperkalemia and drug alerts for patients >65 with a psychotropic drug prescription  **^2^** The studies were done in outpatient settings in the US and Canada  **^3^** The study interventions were supplementing a drug alert with specific patient laboratory data  **^4^** The outcome was % compliance with CDS advice  **^5^**  Duke 2013  **^6^** Downgraded due to risk of bias and the limited amount of studies  **^7^** Tamblyn 2012 | | | |

**5. CDS that does (versus does not) require users to respond to the advice**

Three studies (Meeker 2017, Arts 2017, Litzelman 1993) required the healthcare professional to provide a reason when overriding the CDS advice.^13-15^ In Strom 2010, the clinicians had to confirm that the advice was seen in order to proceed.^16^

*Primary analysis*

Compliance with recommended practice for patients with acute respiratory infections did not improve (0.1% adjusted RD) in Meeker 2017.

*Secondary analysis*

Litzelman 1993 found that compliance with the advice improved for patients with miscellaneous problems, while Arts 2017 and Strom 2010 found that compliance slightly decreased (-2.0% median absolute change (IQR -2.9 to + 8.0). Arts 2017 targeted care for patients with atrial fibrillation and Strom focussed on patients with concurrent orders for warfarin and NSAIDs

*Certainty*

We appraised the risk of bias as high for Meeker 2017 and Strom 2010 and unclear for Arts 2017 and Litzelman 1993. Quality of the evidence according to GRADE was low for process outcomes.

*Summary of findings table*

| **Patient or population**: any patient **^1^**  **Settings**: any setting **^2^**  **Intervention**: CDS that requires a response from the user **^3^**  **Comparison**: CDS that does not require a user response | | | |
| --- | --- | --- | --- |
| **Outcomes** | Result | **Nb of participants (studies)** | **Quality of the evidence (GRADE)** |
| *PRIMARY ANALYSIS* |  |  |  |
| Compliance with desired practice  (follow-up 18 months) | RD 0.1% **^4^** | 4623 patient visits (1 study**^5^**) | ⊕⊕🌕🌕  **Low^6^** |
| *SECONDARY ANALYSIS* |  |  |  |
| Compliance with desired practice  (median follow-up 9 months) | RD -2.0% (IQR -2.9 to + 8.0)**^7^** | 6457 patients (3 studies**^8^**) | ⊕⊕🌕🌕  **Low^6^** |
| **^1^** The studies focussed on patients with acute respiratory infections, atrial fibrillation, patients with concurrent orders for warfarin and NSAIDs and patients with miscellaneous problems  **^2^** Three studies were done in outpatient settings in the US and the Netherlands. One study was done in an inpatient setting in the US.  **^3^** The study interventions were to provide a reason when overriding the CDS advice or to confirm that the advice was seen.  **^4^** The outcome was % of inappropriate antibiotic prescriptions  **^5^**  Meeker 2016  **^6^** Downgraded due to risk of bias and inconsistent results  **^7^**  The outcomes were % compliance with the recommended advice  ^8^ Arts 2017, Litzelman 1993, Strom 2010 | | | |

**6. CDS provided automatically by the system versus on demand by the user**

We included four studies for this comparison.^17-20^

We did not include any studies in the primary analysis, since none of the studies provided baseline outcome data.

*Secondary analysis*

The median absolute improvement in compliance with desired practice increased was 22.2% (IQR +0.55 to +28.0). Both van Wyk 2008 and Scheepers-Hoeks 2013 found large increases in compliance with recommended practice for lipid management or for intensive care unit treatment.

Tamblyn 2008 targeted prescription problems for miscellaneous conditions. They found that the % of prescribing problems seen increased (unadjusted RD 9.4%), but the % of prescribing problems revised did not change (unadjusted RD 0.55%).

Rosenbloom 2005 found that the CDS was accessed more (unadjusted RD +0.11) and that the expenditure per order-entry session (for miscellaneous conditions) was lower ($403.1 vs $408.6). Scheepers-Hoeks 2013 found a 23.3% relative improvement in provider satisfaction.

*Indirect evidence*

Nendaz 2010 also compared CDS that was provided automatically with CDS that was available on demand.^21^ We did not include this study in the comparison since the on demand group also required input of patient data and the CDS used another channel (electronic chart versus personal digital assistant). This study found a higher compliance with recommended practice for acutely ill patients (6.8% adjusted RD).

*Certainty*

We assessed the risk of bias as high in three studies^17-19^ and as unclear in van Wyk 2008^20^. The risk of bias for Nendaz 2010 was also high. Quality of the evidence according to GRADE was low for process outcomes.

*Summary of findings table*

| **Patient or population**: any patient **^1^**  **Settings**: any setting **^2^**  **Intervention**: CDS that is provided automatically by the system  **Comparison**: CDS that is available on demand by the user | | | |
| --- | --- | --- | --- |
| **Outcomes** | Result | **Nb of participants (studies)** | **Quality of the evidence (GRADE)** |
| *PRIMARY ANALYSIS* |  |  |  |
|  | - |  |  |
| *SECONDARY ANALYSIS* |  |  |  |
| Compliance with desired practice  (median follow-up 12 months) | RD 22.2% (IQR +9.4 to +28.0)**^3^**  Prevalence of prescription problems: Odds ratio 1.03 (95% CI 0.80-1.32) | 5795 patients (3 studies**^4^**)  3449 patients (1 study**^6^**) | ⊕⊕🌕🌕  **Low^5^** |
| **^1^** The studies focussed on patients that required intensive care unit treatment, patients with dyslipidemia and patients with miscellaneous conditions.  **^2^** Two studies were done in outpatient settings in Canada and the Netherlands. Two studies were done in inpatient settings in the US and the Netherlands.  **^3^** The outcomes were % of prescribing problems revised, % of compliance with CDS, % of due patients screened/treated  **^4^**  Tamblyn 2008, Scheepers-Hoeks 2013, Van Wyk 2008  **^5^**  Downgraded due to risk of bias and inconsistent results  ^6^ Tamblyn 2008 | | | |

**7. CDS displayed on screen versus delivered on paper**

We identified two studies (Denig 2014, Vinker 2002) for this comparison.^2, 22^ The CDS was directed at the healthcare professionals in Vinker 2002 and at both the clinician and the patient for the purpose of shared decision-making in Denig 2014.

*Primary analysis*

Vinker 2002 found a moderate improvement in screening for colorectal cancer (15.6% adjusted RD).^22^

*Secondary analysis*

Compliance with recommended practice for diabetes was lower in Denig 2014, with odds ratios that ranged from 0.29 to 0.67 (data to present absolute increases were not available). There was no difference in scores on the diabetes empowerment scale.^2^

*Certainty*

The risk of bias was high for Denig 2014 and unclear for Vinker 2002. Quality of the evidence according to GRADE was low for both process and clinical outcomes.

*Summary of findings table*

| **Patient or population**: any patient **^1^**  **Settings**: any setting **^2^**  **Intervention**: CDS that is provided on screen**^3^**  **Comparison**: CDS that is provided on paper | | | |
| --- | --- | --- | --- |
| **Outcomes** | Result | **Nb of participants (studies)** | **Quality of the evidence (GRADE)** |
| *PRIMARY ANALYSIS* |  |  |  |
| Compliance with desired practice  (follow-up 12 months) | RD 15.6% **^4^** | 753 patients (1 study**^5^**) | ⊕⊕🌕🌕  **Low^6^** |
| *SECONDARY ANALYSIS* |  |  |  |
| Compliance with desired practice  (follow-up 15 weeks)  Changes in clinical outcomes (follow-up 6 months) | % patients with intensified glucose treatment, Odds ratio: 0.63 (95% CI 0.19 – 1.96)  % patients with intensified blood pressure treatment, Odds ratio: 0.29 (95% CI 0.09 – 0.95)  % patients with intensified lipid treatment, Odds ratio: 0.34 (95% CI 0.12 – 0.98)  % patients with RAS inhibitors prescribed, Odds ratio: 0.67 (95% CI 0.21 – 2.10)**^7^**  Mean difference -0.026**^8^** | 225 patients  (1 study**^9^**)  225 patients  (1 study**^9^**) | ⊕⊕🌕🌕  **Low^8^**  ⊕⊕🌕🌕  **Low^8^** |
| **^1^** The studies included patients with diabetes and patients due for colorectal cancer screening.  **^2^** The studies were done in outpatient settings in Israel and the Netherlands  **^3^** The study interventions were directed at either the healthcare professional in one study and at both the clinician and the patient to support shared decision making the other study  **^4^** The outcome was % patients with a fecal occult blood test  **^5^** Vinker 2002  **^6^** Downgraded for risk of bias and because the finding is limited to a single study  **^7^** The outcomes were % patients with intensified glucose treatment, intensified blood pressure treatment, intensified lipid treatment, % patients with RAS inhibitors prescribed  **^8^** The outcome was score on diabetes empowerment scale, the value is adjusted for baseline differences  **^9^** Denig 2014 | | | |

**8. CDS provided before versus during the patient visit**

*Indirect evidence*

Bloomfield 2005 compared CDS that was provided some days before the patient visit with CDS provided during the visit.^23^ We did not include this study in table 1 since the trial arms also differed in type of channel to deliver the CDS (message in a notification box versus message on cover page of computerised medical record).

The unadjusted RD in compliance with prescription of lipid lowering drugs disease was 1.3%.

*Certainty*

The risk of bias for this study was unclear. Quality of the evidence according to GRADE was low for process outcomes.

*Summary of findings table*

| **Patient or population**: any patient **^1^**  **Settings**: any setting **^2^**  **Intervention**: CDS that is provided before the patient visit  **Comparison**: CDS that is provided during the patient visit | | | |
| --- | --- | --- | --- |
| **Outcomes** | Result | **Nb of participants (studies)** | **Quality of the evidence (GRADE)** |
| COMPLIANCE  *Primary analysis* | - |  | ⊕⊕🌕🌕  **Low^6^** |
| *Secondary analysis*  (follow-up 12 months) | RD 1.3% **^4^** | 1349 patients (1 study**^7^**) |  |
| **^1^** The study focussed on patients with ischemic heart disease  **^2^** The study was done in an outpatient setting in the US  **^4^** The outcome was % patients with prescription of lipid lowering drugs  **^5^**  Bloomfield 2005  **^6^** Downgraded due to risk of bias and the limited amount of studies | | | |

**9. CDS combined with other professional-oriented strategies versus CDS only**

We identified seven studies that evaluated the role of co-interventions that target healthcare professionals. The co-interventions included education (Dickinson 1981, Feldstein 2006, Fortuna 2009, Simon 2006),^24-27^ performance feedback (Meeker 2017, Ziemer 2006)^15, 28^ and the involvement of opinion leaders (McAlister 2009).^29^ Four studies focussed on conditions including hypertension, diabetes, , and. Three studies focussed on patients with prescriptions for warfarin and for and.

*Primary analysis*

There was a small improvement in compliance with recommended practice (adjusted RD 4.8%, IQR -3.9 to 10.8).^15, 26, 28^ Fortuna 2009 and Ziemer 2006 found that compliance with recommended practice (for prescription of hypnotic medications and treatment of patients with diabetes) was higher. Meeker 2017 found that compliance with recommended practice was lower for acute respiratory infections.

*Secondary analysis*

McAlister 2009 found higher compliance with recommended practice for coronary heart disease (unadjusted RD 6.2%).^29^ Both McAlister 2009 and Simon 2006 (focus on medication prescriptions for elderly patients) found better compliance based on continuous process measures.^27, 29^ Two studies (Dickinson 1981, McAlister 2009) found both positive and negative changes that were small for clinical outcomes.^24, 29^ McAlister 2009 found that utilisation of healthcare services was lower with less hospitalisations (0.8% relative change) and emergency care visits (4.0% relative change).

*Certainty*

The risk of bias was low in Fortuna 2009 and Simon 2006,^26, 27^ unclear in McAlister 2009^29^ and high in the other four studies^15, 24, 25, 28^. Quality of the evidence according to GRADE was low for process outcomes and for patient outcomes.

*Summary of findings table*

| **Patient or population**: any patient **^1^**  **Settings**: any setting **^2^**  **Intervention**: CDS together with a co-intervention**^3^**  **Comparison**: CDS without co-intervention | | | |
| --- | --- | --- | --- |
| **Outcomes** | Result | **Nb of participants (studies)** | **Quality of the evidence (GRADE)** |
| *PRIMARY ANALYSIS* |  |  |  |
| Compliance with desired practice  (median follow-up 18 months) | RD 4.8 % (IQR -3.9 to +10.8) **^4^** | 602 clinicians (3 studies**^5^**) | ⊕⊕🌕🌕  **Low ^6^** |
| *SECONDARY ANALYSIS* |  |  |  |
| Compliance with desired practice  (median follow-up 9 months )  Changes in clinical outcomes (median follow-up 6.5 months) | RD 6.2%  % relative change values: +2.4 (standardised mean statin dose), +4.9 (quarterly rates of use of target medications to avoid/10000 patients) *(+ means improvement)*    RD -0.5% (IQR -5 to +4) **^8^**  % relative change values: -2.3 (mean diastolic blood pressure),  +0.7 (mean systolic blood pressure) *(+ means improvement)* | 354 patients  (1 study**^9^**)  Clinicians from 267 primary care practices and 8 hospital care units  (2 studies**^10^**)    493 patients (2 studies**^11^**) | ⊕⊕🌕🌕  **Low ^6^**  ⊕⊕🌕🌕  **Low^12^** |
| **^1^** The studies included patients with diabetes, coronary heart disease, and acute respiratory infections, patients with prescriptions for warfarin and for hypnotic medications and medication prescriptions for elderly patients.  **^2^** The studies were done in outpatient settings in the US and Canada.  **^3^** The co-interventions included education, performance feedback and the involvement of opinion leaders.  **^4^** The outcomes were % prescriptions for heavily marketed hypnotics, % of inappropriate antibiotic prescriptions, % visits where providers intensified the therapy, % visits where intensification of therapy met recommendations  **^5^** Fortuna 2009, Meeker 2016, Ziemer 2006  **^6^** Downgraded for risk of bias and inconsistencies within the results  **^7^** The primary outcome was not adjusted for baseline differences, but the secondary outcomes were adjusted. The outcomes were % with initiation of statin or increase of dose (primary outcome); % taking a statin, % taking a nonstatin lipid-lowering drug, % taking any antiplatelet agent, % taking an ACE inhibitor or ARB, % taking a β-blocker, % taking triple therapy  **^8^** The outcomes were % patients stopped smoking, % with fasting LDL ≤ 2.0 mmol/L, % with emergency department visits, % with hospitalisations, % of deaths, % patients with controlled diastolic blood pressure, % patients with improved diastolic blood pressure, % patients with improved systolic blood pressure  **^9^** McAlister 2009  **^10^** Simon 2006, McAlister 2009  **^11^** McAlister 2009, Dickinson 1981  **^12^** Downgraded for risk of bias and inconsistencies within the results | | | |

**10. CDS combined with patient-oriented strategies versus CDS only**

Eighteen studies evaluated the effect of patient-directed strategies in combination with CDS for the healthcare providers. The patient strategies included patient information about recommended care^30-44^, patient education or counselling^37, 45^ and forms to be given by the patient to the healthcare provider^42, 46, 47^.

*Primary analysis*

CDS combined with patient-oriented strategies led to a small improvement in compliance with recommended practice (3.1% median absolute improvement, IQR -0.8 to 5.0). ^30, 32, 34, 36, 38, 40, 42, 43, 46, 47^ Bosworth 2011 found that clinical outcomes were worse (-5% adjusted RD).^48^

*Secondary analysis*

The median absolute increase in compliance was small (4.3%, (IQR 2.5 to 6.5).^31, 35, 37, 39, 41, 44^Feldstein 2006a found a negative change for continuous process outcomes.^32^ The results for clinical outcomes were conflicting.^32, 39, 48^

*Subgroup analysis*

We identified fourteen studies for this subgroup analysis. Ten studies focussed on CDS for preventive procedures or screening tests. ^30, 31, 35-37, 40-42, 44, 47^ Four studies focussed on CDS for acute or chronic care.^32, 39, 43, 46^ The RD for CDS on prevention or screening was 3.6% (IQR 1.0 to 5.3) versus an RD of 0.3% (IQR -1.4 to 3.2) for CDS on acute or chronic care (P = 0.38).

*Indirect evidence*

Feldman 2005 and McDonald 2005 evaluated the effect of CDS combined with a strategy that included both professional- and patient-oriented strategies.^49, 50^ McDonald 2005 found an unadjusted RD of 1.1% (IQR -3.8 to +4.9) in compliance with recommended care for cancer pain.^50^ Median absolute change in patient outcomes was 1.5% (-1.4 to +4.3).^49, 50^ The findings for continuous patient outcomes were conflicting. Overall costs (including intervention and utilisation of healthcare services) were higher in Feldman 2005 (6330$ vs 5869$) and lower in McDonald 2005 (5611$ vs 5966$).

*Certainty*

Quality of the evidence according to GRADE was moderate for process outcomes and low for clinical outcomes.

*Summary of findings table*

| **Patient or population**: any patient **^1^**  **Settings**: any setting **^2^**  **Intervention**: CDS combined with patient-oriented strategies^3^  **Comparison**: CDS for healthcare provider only | | | |
| --- | --- | --- | --- |
| **Outcomes** | Result | **Nb of participants (studies)** | **Quality of the evidence (GRADE)** |
| *PRIMARY ANALYSIS* |  |  |  |
| Compliance with desired practice  (median follow-up 6 months)  Clinical outcomes (follow up 18 months) | RD 3.1% (IQR -2.0 to 5.0)**^4^**  RD -5% **^5^** | > 20530 patients (10 studies**^6^**)  296 patients (1 study**^7^**) | ⊕⊕⊕🌕  **Moderate^8^**  ⊕⊕🌕🌕  **Low^9^** |
| *SECONDARY ANALYSIS* |  |  |  |
| Compliance with desired practice  (median follow-up 10.5 months)  Clinical outcomes (median follow up 6 months) | RD 4.3% (IQR 2.5 to 6.5)**^9^**  % relative change values: -14.6 (calcium intake mg/day)  RD +18.2 **^12^**  % relative change values: -2.1 (mean systolic blood pressure), +1.1 (patient medication adherence), +4.3 (systolic blood pressure) *(+ means improvement)* | > 5355 patients (6 studies**^10^**)  311 patients (1 study**^11^**)  720 patients (1 study**^13^**)  1327 patients (3 studies**^14^**) | ⊕⊕⊕🌕  **Moderate^8^**  ⊕⊕🌕🌕  **Low^6^** |
| **^1^** The study focussed on women at risk of having osteoporosis, patients with due preventive procedures, patients eligible for advance directives, patients with diabetes mellitus, congestive heart failure, chronic obstructive pulmonary disease, xerostomia, uncontrolled hypertension, patients with miscellaneous conditions, participants with multiple chronic diseases.  **^2^** The studies were done in outpatient settings in the US and New Zealand.  **^3^** The patient strategies included patient information about recommended care, patient education or counselling and forms to be given by the patient to the healthcare provider.  **^4^** The outcomes were % patients that received a pharmacological treatment or bone mineral density measurement, % patients screened for diabetes, % women with mammography, % patients with a completed advance directive, % patients with completed pap smear, % patients with completed fecal occult blood test, % patients with guaiac test, % patients with completed cholesterol test, % patients with completed tetanus vaccinations, % patients with completed rectal exam, % compliance with the CDS advice, % patients screened, % patients with regular aspirin use, % of patients without cardiovascular disease that receive aspirin  **^5^**  The outcome was % with blood pressure in control  **^6^** Feldstein 2006a, Burack 1996, Heiman 2004, Ornstein 1991, Rosenberg 2008, Sequist 2009, Turner 1989, Persell 2008, Folks 2011, Kenealy 2005  **^7^** Bosworth 2011  **^8^** Downgraded due to risk of bias  ^9^ Downgraded due to risk of bias and inconsistent results  ^10^ Becker 1990, Burack 1998, McPhee 1989, Rimer 1999, Roumie 2006, Simon 2001  ^11^Feldstein 2006a  ^12^ The outcome was % patients with systolic blood pressure ≤140 (primary outcome)  ^13^ Roumie 2006  ^14^ Feldstein 2006a, Roumie 2006, Bosworth 2011 | | | |

**11. CDS aimed at the patient versus CDS aimed at the healthcare provider**

Six studies compared the outcomes of CDS-based patient-directed information versus CDS for the healthcare provider.^30, 31, 36, 51-53^

*Primary analysis*

Data from three studies provide a summary estimate for compliance with recommended practice of 4.3%, IQR -5.8 to +12.5.^30, 36, 51^ Feldstein 2006 found a large increase (17.8%) in compliance for laboratory monitoring after medication prescription. The RD for compliance with preventive procedures was only 1.3% in Ornstein 1991. Burack 1996 found that compliance for breast cancer screening increased (8.9%) in one site and decreased in another (-11.9%).

*Secondary analysis*

Three studies evaluated compliance and found inconsistent results (4.2% median absolute improvement, IQR 0 to +8.3).^31, 52, 53^ Feldstein 2006c and Lobach 2013 found inconsistent results for patient outcomes (-2.4% absolute change, IQR -8.1 to +3.3).^51, 53^ The total medical costs were higher (3077$ vs 2452$) in Lobach 2013 and patient satisfaction measures did not differ. Rosser 1991 found that the costs per blood pressure reading gained and cervical screening gained were lower.

*Certainty*

The risk of bias was low for Feldstein 2006c and high in the five other studies.^30, 31, 36, 52, 53^ Quality of the evidence according to GRADE was low for both process and clinical outcomes.

*Summary of findings table*

| **Patient or population**: any patient **^1^**  **Settings**: any setting **^2^**  **Intervention**: computer generated information that is directed at the patient**^3^**  **Comparison**: CDS for the healthcare provider | | | |
| --- | --- | --- | --- |
| **Outcomes** | Result | **Nb of participants (studies)** | **Quality of the evidence (GRADE)** |
| *PRIMARY ANALYSIS* |  |  |  |
| Compliance with desired practice  (median follow-up 12 months) | RD 5.1% (IQR -5.3 to + 13.4)**^4^** | 4791 patients (3 studies**^5^**) | ⊕⊕🌕🌕  **Low^6^** |
| *SECONDARY ANALYSIS* |  |  |  |
| Compliance with desired practice  (median follow-up 12 months)  Changes in clinical outcomes (median follow-up 5 months) | RD 4.2% (IQR 0 to +8.3)**^7^**  -14.4% relative change (missed appointments/100 patients) *(+ means improvement)*  RD -2.4% IQR (-8.1 to +3.3)**^8^**  -14.7% relative change (emergency department encounters/100 patients) *(+ means improvement)* | 6404 patients (2 studies**^9^**)  10098 (1study)^10^  289 patients (2 studies**^11^**)  10098 patients (1 study)^10^ | ⊕⊕🌕🌕  **Low^6^**  ⊕⊕🌕🌕  **Low^12^** |
| **^1^** The study focussed on patients with due preventive procedures, patients with due laboratory monitoring after medication prescription and patients with miscellaneous conditions.  **^2^** The studies were done in outpatient settings in the US and Canada  **^3^** The information was provided as a computer generated letter in four studies, as an automated telephone message in one study, or partly as a letter or telephone message in another study.  **^4^** The outcomes were % patients with mammography, pap smear, fecal occult blood test, cholesterol test, tetanus vaccinations, % patients with completed baseline laboratory monitoring  **^5^**  Burack 1996a, Burack 1996b, Feldstein 2006c, Ornstein 1991  **^6^** Downgraded due to risk of bias and inconsistent results  **^7^** The outcomes were % patients with due preventive procedures performed, % patients with pap smear test  **^8^** The outcomes were % of patients with abnormal test results, % rating excellent or very good on general health status scale  **^9^**  Burack 1998, Rosser 1991  **^10^** Lobach 2013  **^11^** Feldstein 2006c, Lobach 2013  **^12^** Downgraded due to risk of bias and inconsistent results. | | | |

**12. Comparisons related to CDS and staff-oriented interventions**

Loo 2011 and Willis 2013 compared CDS for clinicians combined with support from a case manager who assisted patients and physicians versus CDS for clinicians only.^54, 55^ The studies focussed on elderly patients with due preventive procedures and on patients with asthma, diabetes, hypertension, congestive heart failure, ischemic heart disease or stroke.

Scheepers-Hoeks 2013, Murray 2004 and Tierney 2005 compared CDS provided to pharmacists (or to both pharmacists and physicians) versus CDS for physicians.^18, 56, 57^ The studies included patients with hypertension, asthma and chronic obstructive pulmonary disease and patients requiring intensive care unit treatment.

Utidjian 2015 compared CDS for nurses and physicians versus CDS for nurses only.^58^ Dexter 2004 compared CDS for nurses versus CDS for physicians.^59^ The studies focussed on preventive treatment for premature infants and patients discharged from the hospital that were eligible for vaccination.

Christakis 2004 compared CDS for patient schedulers (or to both schedulers and physicians) versus CDS for physicians.^60^ The study focussed on pediatric patients for whom continuity of care was suboptimal.

*Primary analysis*

Providing CDS to the physician and an additional staff role modestly improved compliance with preventive procedures for elderly patients (4.1 % adjusted RD, IQR +3.4 to +7.2) in Loo 2011.^54^ Dexter 2004 found that CDS directed at nurses versus physicians improved compliance with administration of vaccinations with 16.5%, (IQR +12.7 to +20.3 ).^59^ Utidjian 2015 found that CDS for nurses and physicians versus nurses only decreased compliance with recommended practice for premature infants (-13.7% adjusted RD).^58^

*Secondary analysis*

The median absolute increase in compliance with desired practice was 5.2% (IQR +4.4 to +6) when CDS was provided to an additional staff role.^56, 57^ Christakis 2004 found no effect on continuous process outcomes.^60^ Three studies found both positive and negative results for patient outcomes. ^55-57^ Both Tierney 2005 and Murray 2004 found that total health care charges were lower (3122$ vs 6200$ and 5652$ vs 8006$). Patient satisfaction did not change in Tierney 2005.

The comparison of CDS for pharmacists versus CDS for physicians provided conflicting results.^18, 56, 57^ Total health care charges were 5445$ vs 6200$ in Murray 2004 and 5333$ vs 8006$ in Tierney 2005.

*Indirect evidence*

Bates 1998 compared the effect of CDS supplemented with a multicomponent strategy that included changes in the role of the pharmacist (together with implementing a pharmacy communication log, standardised labelling of intravenous bags, dilution charts, provision of drip-rate calculators).^61^ This study found that the results for the process outcomes were worse with the multicomponent strategy (25% relative change in rate of non-intercepted serious medication errors/100 patient days).

*Certainty*

The risk of bias was unclear in Willis 2013 and Utidjian 2015 and high in the six other studies. The risk of bias for Bates 1998 was high. Quality of the evidence according to GRADE was moderate for process outcomes and low for patient outcomes.

*Summary of findings table*

| **Patient or population**: any patient **^1^**  **Settings**: any setting **^2^**  **Intervention**: CDS for the physician combined with CDS for an additional staff role**^3^**  **Comparison**: CDS for physician | | | |
| --- | --- | --- | --- |
| **Outcomes** | Result | **Nb of participants (studies)** | **Quality of the evidence (GRADE)** |
| *PRIMARY ANALYSIS* |  |  |  |
| Compliance with desired practice  (follow-up 12 months) | RD 4.1 % (IQR +3.4 to +7.2)**^4^** | 2730 patients (1 study**^5^**) | ⊕⊕⊕🌕  **Moderate ^6^** |
| *SECONDARY ANALYSIS* |  |  |  |
| Compliance with desired practice  (median follow-up 12 months)  Changes in clinical outcomes (median follow-up 12 months) | 5.2% RD (IQR +4.4 to +6)**^7^**  0% relative change^8^  RD 1.35% (IQR +1 to +1.7)**^9^**  % relative change values: -10.9 (health-related quality of life), -5.4 (generic health-related quality of life), 0 (emergency department visits), +20 (all cause hospitalisations) *(+ means improvement)* | 584 patients ( 2 studies^10^)  220 patients ( 1 study**^11^**)  1733 patients (2 studies**^12^**)  2217 Patients (3 studies^13^) | ⊕⊕⊕🌕  **Moderate ^6^**  ⊕⊕🌕🌕  **Low^14^** |
| **^1^** The study focussed on elderly patients with due preventive procedures and on patients due for vaccinations and patients with asthma, diabetes, hypertension, congestive heart failure, ischemic heart disease, stroke, chronic obstructive pulmonary disease and patients requiring intensive care unit treatment. One study focussed on pediatric patients for whom continuity of care was suboptimal.  **^2^** Five studies were done in an outpatient setting in the US. Two studies were done in inpatient settings in the US and the Netherlands.  **^3^** The additional staff roles included patient schedulers, case managers and pharmacists  **^4^** The outcomes were % patients with healthcare proxy designation, % patients with bone density screening, % patients with peumococcal vaccination, % patients with influenza vaccination  **^5^**  Loo 2011  **^6^** Downgraded due to risk of bias  **^7^** The outcomes were % compliance with CDS  **^8^** The outcome was continuity of care score  **^9^** The outcomes were % patients with medication adherence  **^10^**  Tierney 2005, Murray 2004  **^11^** Christakis 2004  **^12^** Willis 2013, Tierney 2005  ^13^ Willis 2013, Tierney 2005, Murray 2004  **^14^** Downgraded due to risk of bias and inconsistent results. | | | |

**13. Comparisons in relation to the usability of the CDS system**

El-Kareh 2011 compared CDS that was linked to an order entry system to facilitate ordering of recommended laboratory tests and radiology studies versus CDS that was not linked to an order entry system.^62^ The study focussed on patients with due preventive procedures. The adjusted RD for compliance with the recommended practice was 1.4% (IQR +0.2 to +5.9). Robbins 2012 also compared CDS that facilitated provider orders.^63^ Here the comparison groups also differed in the delivery channels for the CDS. This study found that process outcomes improved. Patient outcomes changed both in positive and negative directions.

Del Fiol 2008 compared infobuttons with links that pointed to specific questions or content versus infobuttons that provided less specific links.^64^ The study measured dichotomous and continuous process measures. No baseline outcome data were available for the dichotomous measure. The number of infobutton searches was higher (median of 22 versus 17.5) and less time was spent to seek information (median time spent seeking information: 35.5 vs 43 seconds). The % of searches where the needed information was found was lower with the specific links (-5.7% absolute change). The relative % improvement for impact of the search on medical decisions was 3.8.

Hendrix 2015 study compared colour-highlighted CDS advice with not highlighted advice in one set of prompts.^65^ Patients had miscellaneous paediatric conditions. The unadjusted RD for compliance varied with a median of 3% (IQR -22 to +15).

*Certainty*

We evaluated the risk of bias for as unclear for Del Fiol 2008 and Hendrix 2015 and as high risk for El Kareh 2011 and Robbins 2012. Quality of the evidence according to GRADE was low for process outcomes.

*Summary of findings table*

| **Patient or population**: any patient **^1^**  **Settings**: any setting **^2^**  **Intervention**: CDS with extra features to increase the usability  **Comparison**: CDS without the extra features | | | |
| --- | --- | --- | --- |
| **Outcomes** | Result | **Nb of participants (studies)** | **Quality of the evidence (GRADE)** |
| COMPLIANCE  *Primary analysis* | RD 1.4% (IQR +0.2 to +5.9) | 8 practices (1 study^4^) | ⊕⊕🌕🌕  **Low^6^** |
| (follow-up 6 months)  *Secondary analysis*  (follow-up 3 months) | RD 3% (IQR -22 to +15) | Nb unclear (1 study^5^) |  |
| **^1^** The study focussed on patients with ischemic heart disease, miscellaneous paediatric conditions  **^2^** The studies were done in outpatient settings in the US  **^4^** El-Kareh 2011  **^5^**  Hendrix 2015  **^6^** Downgraded due to risk of bias and the limited amount of studies | | | |

**14. Comparisons in relation to the amount of CDS**

Chambers 1991 assessed the effect of providing CDS in all the eligible situations versus limiting the CDS to half of the relevant situations.^66^ This study focussed on influenza vaccinations for elderly patients or patients at risk.

*Secondary analysis*

Compliance was 51% when CDS was always provided and 28% when CDS was sometimes provided (unadjusted RD of 21.8%).

Within the group where CDS was sometimes provided, compliance was 38% when the reminder displayed and 20% when the reminder did not display. Compliance for this subgroup of patients where the healthcare provider was not reminded for some patients was actually lower than compliance in a control group where providers never received CDS (30%).

*Certainty*

The risk of bias for this study was high. Quality of the evidence according to GRADE was low for process outcomes.

*Summary of findings table*

| **Patient or population**: any patient **^1^**  **Settings**: any setting **^2^**  **Intervention**: CDS that is always displayed  **Comparison**: CDS that is displayed for a random selection of patients | | | |
| --- | --- | --- | --- |
| **Outcomes** | Result | **Nb of participants (studies)** | **Quality of the evidence (GRADE)** |
| *PRIMARY ANALYSIS* |  |  |  |
|  | - |  |  |
| *SECONDARY ANALYSIS* |  |  |  |
| Compliance with desired practice  (follow-up 6 months) | RD 21.8% **^4^** | 447 patients (1 study**^7^**) | ⊕⊕🌕🌕  **Low^6^** |
| **^1^** The study focussed on influenza vaccinations for elderly patients or patients at risk  **^2^** The study was done in an outpatient setting in the US  **^4^** The outcome was % compliance with CDS advice  **^5^**  Chalmers 1991  **^6^** Downgraded due to risk of bias and the limited amount of studies | | | |

References

1. McDonald CJ, Wilson GA, McCabe GP, Jr. Physician response to computer reminders. JAMA. 1980;244(14):1579-81.

2. Denig P, Schuling J, Haaijer-Ruskamp F, Voorham J. Effects of a patient oriented decision aid for prioritising treatment goals in diabetes: pragmatic randomised controlled trial. BMJ. 2014;349:g5651.

3. Simon GE, VonKorff M, Rutter C, Wagner E. Randomised trial of monitoring, feedback, and management of care by telephone to improve treatment of depression in primary care. BMJ (Clinical research ed). 2000;320(7234):550-4.

4. Skinner CS, Halm EA, Bishop WP, Ahn C, Gupta S, Farrell D, et al. Impact of Risk Assessment and Tailored versus Nontailored Risk Information on Colorectal Cancer Testing in Primary Care: A Randomized Controlled Trial. Cancer epidemiology, biomarkers & prevention : a publication of the American Association for Cancer Research, cosponsored by the American Society of Preventive Oncology [Internet]. 2015; 24(10):[1523-30 pp.]. Available from: <http://onlinelibrary.wiley.com/o/cochrane/clcentral/articles/365/CN-01170365/frame.html>.

5. Subramanian U, Fihn SD, Weinberger M, Plue L, Smith FE, Udris EM, et al. A controlled trial of including symptom data in computer-based care suggestions for managing patients with chronic heart failure. The American journal of medicine. 2004;116(6):375-84.

6. van Wijk MA, van der Lei J, Mosseveld M, Bohnen AM, van Bemmel JH. Assessment of decision support for blood test ordering in primary care. a randomized trial. Annals of internal medicine. 2001;134(4):274-81.

7. Carroll AE, Biondich P, Anand V, Dugan TM, Downs SM. A randomized controlled trial of screening for maternal depression with a clinical decision support system. Journal of the American Medical Informatics Association : JAMIA. 2013;20(2):311-6.

8. Schwarz EB, Parisi SM, Handler SM, Koren G, Cohen ED, Shevchik GJ, et al. Clinical decision support to promote safe prescribing to women of reproductive age: a cluster-randomized trial. Journal of general internal medicine. 2012;27(7):831-8.

9. Derose SF, Dudl JR, Benson VM, Contreras R, Nakahiro RK, Ziel FH. Point-of-Service reminders for prescribing cardiovascular medications. The American journal of managed care. 2005;11(5):298-304.

10. Manns B, Tonelli M, Culleton B, Faris P, McLaughlin K, Chin R, et al. A cluster randomized trial of an enhanced eGFR prompt in chronic kidney disease. Clinical journal of the American Society of Nephrology [Internet]. 2012; 7(4):[565-72 pp.]. Available from: <http://onlinelibrary.wiley.com/o/cochrane/clcentral/articles/679/CN-00848679/frame.html>.

11. Duke JD, Li X, Dexter P. Adherence to drug-drug interaction alerts in high-risk patients: a trial of context-enhanced alerting. Journal of the American Medical Informatics Association : JAMIA. 2013;20(3):494-8.

12. Tamblyn R, Eguale T, Buckeridge DL, Huang A, Hanley J, Reidel K, et al. The effectiveness of a new generation of computerized drug alerts in reducing the risk of injury from drug side effects: a cluster randomized trial. Journal of the American Medical Informatics Association : JAMIA. 2012;19(4):635-43.

13. Arts DL, Abu-Hanna A, Medlock SK, van Weert HC. Effectiveness and usage of a decision support system to improve stroke prevention in general practice: A cluster randomized controlled trial. PloS one. 2017;12(2):e0170974.

14. Litzelman DK, Dittus RS, Miller ME, Tierney WM. Requiring physicians to respond to computerized reminders improves their compliance with preventive care protocols. Journal of general internal medicine. 1993;8(6):311-7.

15. Meeker D, Linder JA, Fox CR, Friedberg MW, Persell SD, Goldstein NJ, et al. Effect of Behavioral Interventions on Inappropriate Antibiotic Prescribing Among Primary Care Practices: A Randomized Clinical Trial. Jama [Internet]. 2016; 315(6):[562-70 pp.]. Available from: <http://onlinelibrary.wiley.com/o/cochrane/clcentral/articles/111/CN-01131111/frame.html>.

16. Strom BL, Schinnar R, Bilker W, Hennessy S, Leonard CE, Pifer E. Randomized clinical trial of a customized electronic alert requiring an affirmative response compared to a control group receiving a commercial passive CPOE alert: NSAID--warfarin co-prescribing as a test case. Journal of the American Medical Informatics Association : JAMIA [Internet]. 2010; 17(4):[411-5 pp.]. Available from: <http://onlinelibrary.wiley.com/o/cochrane/clcentral/articles/551/CN-00761551/frame.html>.

17. Rosenbloom ST, Geissbuhler AJ, Dupont WD, Giuse DA, Talbert DA, Tierney WM, et al. Effect of CPOE user interface design on user-initiated access to educational and patient information during clinical care. Journal of the American Medical Informatics Association : JAMIA. 2005;12(4):458-73.

18. Scheepers-Hoeks A-MJ, Grouls RJ, Neef C, Ackerman EW, Korsten EH. Physicians' responses to clinical decision support on an intensive care unit--comparison of four different alerting methods. Artificial intelligence in medicine. 2013;59(1):33-8.

19. Tamblyn R, Huang A, Taylor L, Kawasumi Y, Bartlett G, Grad R, et al. A randomized trial of the effectiveness of on-demand versus computer-triggered drug decision support in primary care. Journal of the American Medical Informatics Association : JAMIA. 2008;15(4):430-8.

20. van Wyk JT, van Wijk MAM, Sturkenboom MCJM, Mosseveld M, Moorman PW, van der Lei J. Electronic alerts versus on-demand decision support to improve dyslipidemia treatment: a cluster randomized controlled trial. Circulation. 2008;117(3):371-8.

21. Nendaz MR, Chopard P, Lovis C, Kucher N, Asmis LM, Dorffler J, et al. Adequacy of venous thromboprophylaxis in acutely ill medical patients (IMPART): multisite comparison of different clinical decision support systems. Journal of thrombosis and haemostasis : JTH. 2010;8(6):1230-4.

22. Vinker S, Nakar S, Rosenberg E, Kitai E. The role of family physicians in increasing annual fecal occult blood test screening coverage: a prospective intervention study. The Israel Medical Association journal : IMAJ. 2002;4(6):424-5.

23. Bloomfield HE, Nelson DB, van Ryn M, Neil BJ, Koets NJ, Basile JN, et al. A trial of education, prompts, and opinion leaders to improve prescription of lipid modifying therapy by primary care physicians for patients with ischemic heart disease. Quality & safety in health care. 2005;14(4):258-63.

24. Dickinson JC, Warshaw GA, Gehlbach SH, Bobula JA, Muhlbaier LH, Parkerson GR, Jr. Improving hypertension control: impact of computer feedback and physician education. Medical care. 1981;19(8):843-54.

25. Feldstein AC, Smith DH, Perrin N, Yang X, Simon SR, Krall M, et al. Reducing warfarin medication interactions: an interrupted time series evaluation. Archives of internal medicine. 2006;166(9):1009-15.

26. Fortuna RJ, Zhang F, Ross-Degnan D, Campion FX, Finkelstein JA, Kotch JB, et al. Reducing the prescribing of heavily marketed medications: a randomized controlled trial. Journal of general internal medicine. 2009;24(8):897-903.

27. Simon SR, Smith DH, Feldstein AC, Perrin N, Yang X, Zhou Y, et al. Computerized prescribing alerts and group academic detailing to reduce the use of potentially inappropriate medications in older people. Journal of the American Geriatrics Society. 2006;54(6):963-8.

28. Ziemer DC, Doyle JP, Barnes CS, Branch WT, Jr., Cook CB, El-Kebbi IM, et al. An intervention to overcome clinical inertia and improve diabetes mellitus control in a primary care setting: Improving Primary Care of African Americans with Diabetes (IPCAAD) 8. Archives of internal medicine. 2006;166(5):507-13.

29. McAlister FA, Fradette M, Majumdar SR, Williams R, Graham M, McMeekin J, et al. The Enhancing Secondary Prevention in Coronary Artery Disease trial. CMAJ : Canadian Medical Association journal = journal de l'Association medicale canadienne. 2009;181(12):897-904.

30. Burack RC, Gimotty PA, George J, Simon MS, Dews P, Moncrease A. The effect of patient and physician reminders on use of screening mammography in a health maintenance organization. Results of a randomized controlled trial. Cancer. 1996;78(8):1708-21.

31. Burack RC, Gimotty PA, George J, McBride S, Moncrease A, Simon MS, et al. How reminders given to patients and physicians affected Pap smear use in a health maintenance organization: Results of a randomized controlled trial. Cancer. 1998;82(12):2391-400.

32. Feldstein A, Elmer PJ, Smith DH, Herson M, Orwoll E, Chen C, et al. Electronic medical record reminder improves osteoporosis management after a fracture: a randomized, controlled trial. Journal of the American Geriatrics Society. 2006;54(3):450-7.

33. Fricton J, Rindal DB, Rush W, Flottemesch T, Vazquez G, Thoele MJ, et al. The effect of electronic health records on the use of clinical care guidelines for patients with medically complex conditions. Journal of the American Dental Association (1939). 2011;142(10):1133-42.

34. Heiman H, Bates DW, Fairchild D, Shaykevich S, Lehmann LS. Improving completion of advance directives in the primary care setting: a randomized controlled trial. The American journal of medicine. 2004;117(5):318-24.

35. McPhee SJ, Bird JA, Jenkins CN, Fordham D. Promoting cancer screening. A randomized, controlled trial of three interventions. Archives of internal medicine. 1989;149(8):1866-72.

36. Ornstein SM, Garr DR, Jenkins RG, Rust PF, Arnon A. Computer-generated physician and patient reminders. Tools to improve population adherence to selected preventive services. The Journal of family practice. 1991;32(1):82-90.

37. Rimer BK, Conaway M, Lyna P, Glassman B, Yarnall KS, Lipkus I, et al. The impact of tailored interventions on a community health center population. Patient education and counseling. 1999;37(2):125-40.

38. Rosenberg SN, Shnaiden TL, Wegh AA, Juster IA. Supporting the patient's role in guideline compliance: a controlled study. American journal of managed care [Internet]. 2008; 14(11):[737-44 pp.]. Available from: <http://onlinelibrary.wiley.com/o/cochrane/clcentral/articles/821/CN-00738821/frame.html>.

39. Roumie CL, Elasy TA, Greevy R, Griffin MR, Liu X, Stone WJ, et al. Improving blood pressure control through provider education, provider alerts, and patient education: a cluster randomized trial. Annals of internal medicine. 2006;145(3):165-75.

40. Sequist TD, Zaslavsky AM, Marshall R, Fletcher RH, Ayanian JZ. Patient and physician reminders to promote colorectal cancer screening: a randomized controlled trial. Archives of internal medicine. 2009;169(4):364-71.

41. Simon MS, Gimotty PA, Moncrease A, Dews P, Burack RC. The effect of patient reminders on the use of screening mammography in an urban health department primary care setting. Breast cancer research and treatment. 2001;65(1):63-70.

42. Turner BJ, Day SC, Borenstein B. A controlled trial to improve delivery of preventive care: physician or patient reminders? Journal of general internal medicine. 1989;4(5):403-9.

43. Persell SD, Denecke-Dattalo TA, Dunham DP, Baker DW. Patient-directed intervention versus clinician reminders alone to improve aspirin use in diabetes: a cluster randomized trial. Joint Commission journal on quality and patient safety / Joint Commission Resources. 2008;34(2):98-105.

44. Becker DM, Gomez EB, Kaiser DL, Yoshihasi A, Hodge RH. Improving preventive care at a medical clinic: how can the patient help? American journal of preventive medicine. 1990;5(6):353-9.

45. Bosworth H, Olsen M, McCant F, Grubber J, Gentry P, Rose C, et al. Hypertension intervention nurse telemedicine study (HINTS): Testing a multifactorial tailored behavioral/educational and a medication management intervention for blood pressure control. Journal of General Internal Medicine. 2010;25:S298-S9.

46. Folks B, Leblanc WG, Staton EW, Pace WD. Reconsidering low-dose aspirin therapy for cardiovascular disease: a study protocol for physician and patient behavioral change. Implementation science : IS. 2011;6:65.

47. Kenealy T, Arroll B, Petrie KJ. Patients and computers as reminders to screen for diabetes in family practice. Randomized-controlled trial. Journal of general internal medicine. 2005;20(10):916-21.

48. Bosworth HB, Powers BJ, Olsen MK, McCant F, Grubber J, Smith V, et al. Home blood pressure management and improved blood pressure control: results from a randomized controlled trial. Arch Intern Med. 2011;171(13):1173-80.

49. Feldman PH, Murtaugh CM, Pezzin LE, McDonald MV, Peng TR. Just-in-time evidence-based e-mail "reminders" in home health care: impact on patient outcomes. Health services research. 2005;40(3):865-85.

50. McDonald MV, Pezzin LE, Feldman PH, Murtaugh CM, Peng TR. Can just-in-time, evidence-based "reminders" improve pain management among home health care nurses and their patients? Journal of pain and symptom management. 2005;29(5):474-88.

51. Feldstein AC, Smith DH, Perrin N, Yang X, Rix M, Raebel MA, et al. Improved therapeutic monitoring with several interventions: a randomized trial. Archives of internal medicine. 2006;166(17):1848-54.

52. Rosser WW, McDowell I, Newell C. Use of reminders for preventive procedures in family medicine. CMAJ : Canadian Medical Association journal = journal de l'Association medicale canadienne. 1991;145(7):807-14.

53. Lobach DF, Kawamoto K, Anstrom KJ, Silvey GM, Willis JM, Johnson FS, et al. A randomized trial of population-based clinical decision support to manage health and resource use for Medicaid beneficiaries. Journal of medical systems. 2013;37(1):9922.

54. Loo TS, Davis RB, Lipsitz LA, Irish J, Bates CK, Agarwal K, et al. Electronic medical record reminders and panel management to improve primary care of elderly patients. Archives of internal medicine. 2011;171(17):1552-8.

55. Willis JM, Edwards R, Anstrom KJ, Johnson FS, Del Fiol G, Kawamoto K, et al. Decision support for evidence-based pharmacotherapy detects adherence problems but does not impact medication use. Studies in health technology and informatics. 2013;183:116-25.

56. Murray MD, Harris LE, Overhage JM, Zhou X-H, Eckert GJ, Smith FE, et al. Failure of computerized treatment suggestions to improve health outcomes of outpatients with uncomplicated hypertension: results of a randomized controlled trial. Pharmacotherapy. 2004;24(3):324-37.

57. Tierney WM, Overhage JM, Murray MD, Harris LE, Zhou XH, Eckert GJ, et al. Can computer-generated evidence-based care suggestions enhance evidence-based management of asthma and chronic obstructive pulmonary disease? A randomized, controlled trial. Health services research [Internet]. 2005; 40(2):[477-97 pp.]. Available from: <http://onlinelibrary.wiley.com/o/cochrane/clcentral/articles/838/CN-00512838/frame.html>.

58. Utidjian LH, Hogan A, Michel J, Localio AR, Karavite D, Song L, et al. Clinical Decision Support and Palivizumab: A Means to Protect from Respiratory Syncytial Virus. Applied clinical informatics. 2015;6(4):769-84.

59. Dexter PR, Perkins SM, Maharry KS, Jones K, McDonald CJ. Inpatient computer-based standing orders vs physician reminders to increase influenza and pneumococcal vaccination rates: a randomized trial. JAMA. 2004;292(19):2366-71.

60. Christakis DA, Wright JA. Can continuity of care be improved? Results from a randomized pilot study. Ambulatory pediatrics : the official journal of the Ambulatory Pediatric Association. 2004;4(4):336-9.

61. Bates DW, Leape LL, Cullen DJ, Laird N, Petersen LA, Teich JM, et al. Effect of computerized physician order entry and a team intervention on prevention of serious medication errors. JAMA. 1998;280(15):1311-6.

62. El-Kareh RE, Gandhi TK, Poon EG, Newmark LP, Ungar J, Orav EJ, et al. Actionable reminders did not improve performance over passive reminders for overdue tests in the primary care setting. Journal of the American Medical Informatics Association : JAMIA. 2011;18(2):160-3.

63. Robbins GK, Lester W, Johnson KL, Chang Y, Estey G, Surrao D, et al. Efficacy of a clinical decision-support system in an HIV practice: a randomized trial. Annals of internal medicine. 2012;157(11):757-66.

64. Del Fiol G, Haug PJ, Cimino JJ, Narus SP, Norlin C, Mitchell JA. Effectiveness of topic-specific infobuttons: a randomized controlled trial. Journal of the American Medical Informatics Association : JAMIA. 2008;15(6):752-9.

65. Hendrix KS, Downs SM, Carroll AE. Pediatricians' responses to printed clinical reminders: Does highlighting prompts improve responsiveness? Academic Pediatrics. 2015;15(2):158-64.

66. Chambers CV, Balaban DJ, Carlson BL, Grasberger DM. The effect of microcomputer-generated reminders on influenza vaccination rates in a university-based family practice center. The Journal of the American Board of Family Practice / American Board of Family Practice. 1991;4(1):19-26.
